# Identification of broad-spectrum M^pro^ inhibitors: a focus on high-risk coronaviruses and conserved interactions

**DOI:** 10.1080/14756366.2025.2503961

**Published:** 2025-05-21

**Authors:** Man Liu, Li Zhao, Xupeng Huang, Zhenhao Tang, Yihang Zhong, Mengrong Yan, Shun Liu, Shunjing Wang, Zeyun Sun, Zixuan Rao, Linyi Yu, Yuying Fang, Wei Zhang, Hongbo Zhang, Wei Peng

**Affiliations:** a Department of Infectious Diseases, The Key Laboratory of Advanced Interdisciplinary Studies, The First Affiliated Hospital, Guangzhou Medical University, State Key Laboratory of Respiratory Disease, Guangzhou, China; b Innovative Center for Pathogen Research, Guangzhou National Laboratory, Guangzhou, China, China; c Guangzhou Medical University, Guangzhou, China; d University of South China, Hengyang, China; e Beijing StoneWise Technology Co. Ltd, Beijing, China; f Lead contact

**Keywords:** Broad-spectrum, conserved interactions, M^pro^ inhibitors

## Abstract

The COVID-19 pandemic underscores the urgent need to develop broad-spectrum antivirals against coronaviruses (CoVs) to prepare for future outbreaks. In this study, we presented a systematic approach to developing broad-spectrum M^pro^ inhibitors, with a focus on high-risk CoVs. We optimised **S-217622** as a lead compound, with the goal of enhancing conserved interactions within the S1, S2, and S3/S4 pockets of M^pro^, leading to significantly improved inhibitory potency against representative CoVs. Compound **25** exhibited submicromolar activity across all ten CoVs, with IC_50_ values below 0.1 μM for six of them. The X-ray co-crystal structure of SARS-CoV-2 M^pro^ in complex with compound **25** revealed the structural basis of conserved interactions contributing to its broad-spectrum activity. This study demonstrates the feasibility of reinforcing conserved interactions to develop M^pro^ inhibitors with broad-spectrum activity and provides valuable strategies for combating future pandemics caused by unknown CoVs.

## Graphical Abstract

**Figure:**
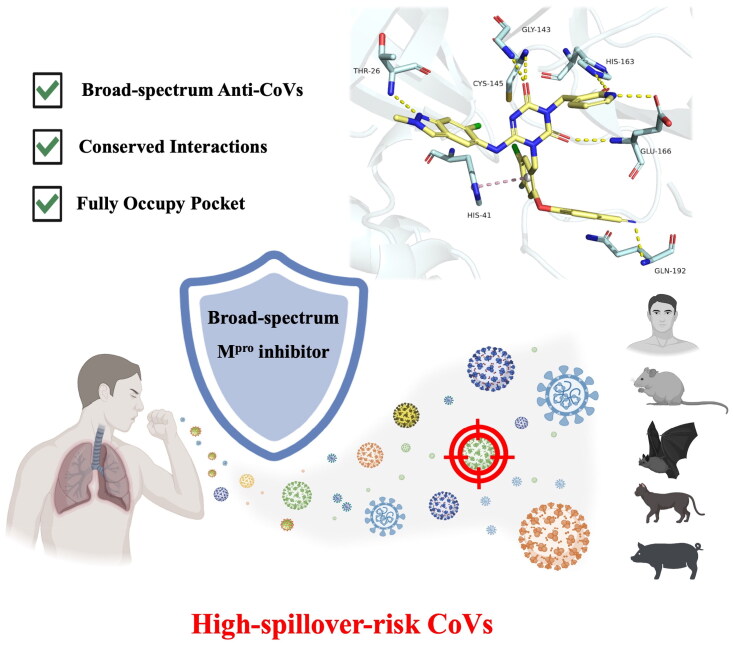

## Introduction

The emergence of Severe Acute Respiratory Syndrome Coronavirus 2 (SARS-CoV-2)^1^^,^^2^ represents a global crisis caused by a third novel coronavirus (CoVs), following the outbreaks of SARS-CoV-1^3^^,^^4^ and Middle East Respiratory Syndrome Coronavirus (MERS-CoV)^5^^,^^6^. Although the COVID-19 pandemic has been controlled, the risk of novel CoVs emerging remains substantial^7^, driven by factors such as zoonotic spill-over events^8^^,^^9^ and viral evolution^10^, potentially resulting in severe global health and economic burdens worldwide. To mitigate these risks, it is imperative to adopt proactive strategies, including the development and strategic stockpiling of broad-spectrum antiviral inhibitors targeting CoVs^11^. These inhibitors, specifically designed to target CoVs with a high potential for zoonotic spill-over and significant pathogenicity, represent a critical inhibitor reserve for combating future CoVs outbreaks. By taking these measures in advance, we can significantly enhance our preparedness and response capacity, thereby reducing the potential threats posed by emerging CoVs.

**Figure 1.**
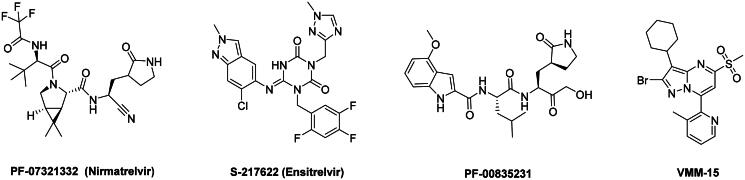
Structures of broad-spectrum anti-CoVs M^pro^ inhibitors.

The main protease (M^pro^), also referred to as non-structural protein 5 (nsp5), has emerged as a prominent drug target for CoVs^12–14^. M^pro^ is responsible for mediating the proteolytic cleavage of viral polyproteins pp1a and pp1ab into various functional proteins essential for viral replication^2^^,^^15^. Additionally, M^pro^ exhibits high conservation within its active site across multiple CoVs^16–18^, positioning it as a promising target for broad-spectrum antiviral drug development. M^pro^ also features unique substrate specificity distinct from human proteases^10^, ensuring that M^pro^ inhibitors do not lead to non-specific inhibition of host proteases. Notably, several M^pro^ inhibitors^19^^,^^20^, such as **PF-07321332** (**Nirmatrelvir**)^21^ and **S-217622** (**Ensitrelvir**)^22^ (Figure 1), have already been clinically approved. These inhibitors highlight the established precedence and abundant knowledge available for targeting M^pro^, underscoring its therapeutic potential.

Recent investigations have increasingly prioritised the development of broad-spectrum M^pro^ inhibitors^23–30^. For example, **PF-07321332** (**Nirmatrelvir**) has demonstrated potent M^pro^ inhibitory activity against seven human infection CoVs (HCoVs), establishing its efficacy as a broad-spectrum anti-CoV agent. Similarly, **S-217622** (**Ensitrelvir**)^27^ has exhibited broad-spectrum M^pro^ inhibitory activity against five HCoVs. Furthermore, **PF-00835231**^31^ and **VMM-15**^32^ have exhibited significant inhibitory activity against a range of CoVs from different species, emphasising their potential on targeting a broad spectrum across diverse CoVs (Figure 1; Table 1). These studies have primarily focused on the diversity and breadth of these inhibitors against currently identified CoVs. However, the prioritisation of high-risk coronaviruses within this spectrum, which are more likely to evolve into serious human health threats due to zoonotic spill-over, viral evolution, and host adaptation, has received insufficient attention. Proactively addressing high-risk CoVs and overcoming the limitations of current broad-spectrum M^pro^ inhibitors represent a promising strategy to effectively combat emerging unknown CoVs outbreak.

Recently, a comprehensive assessment of 40 *alpha*- and *beta*-coronavirus (CoV) species systematically evaluated their zoonotic potential based on population, genetic diversity, receptors, and host species. Among them, 20 species were classified as high-risk: 6 have already crossed into humans, 3 show evidence of spill-over without confirmed human infection, and 11 currently lack spill-over evidence^33^. Broad-spectrum inhibitors designed specifically to target these high-risk CoVs—rather than all known CoVs or human-infecting CoVs (HCoVs)—offer a more feasible and effective therapeutic strategy. This focused approach concentrates on viruses with the greatest likelihood of zoonotic emergence and higher pathogenic potential, thereby improving the chances of successfully mitigating future outbreaks. Such inhibitors serve as a strategically valuable molecular reserve for pre-emptive defense against novel CoVs with pandemic potential.

The goal of this work was to develop broad-spectrum M^pro^ inhibitors targeting the high-risk CoVs, including *beta*-CoVs and *alpha*-CoVs, with a particular emphasis on enhancing broad-spectrum activity through the optimisation of conserved interactions. We selected a known SARS-CoV-2 M^pro^ inhibitor **S-217622** (**Ensitrelvir**) as a scaffold to investigate the impact of occupying different binding pockets on broad-spectrum M^pro^ inhibitory activity. Our design strategy was based on the sequence homology of viral M^pro^, prioritising *beta*-CoVs with high similarity to SARS-CoV-2, followed by *alpha-*CoVs with lower homology. Comparative sequence analysis across different CoVs provided additional insights into the structural determinants of its broad-spectrum efficacy, highlighting the promise of this approach for combating future CoV threats. This sequential approach facilitated the identification of optimal fragments for each binding pocket, yielding compound **25** with outstanding broad-spectrum M^pro^ inhibitory activity, exemplified by an IC_50_ below 0.6 μM against ten high-risk CoVs. Through structure–activity relationship (SAR) studies, we validated that strengthening conserved interactions is a feasible strategy for improving broad-spectrum efficacy. For instance, incorporating tailored fragments into the S3/S4 pocket significantly improved M^pro^ inhibition across diverse CoVs. Moreover, we resolved the X-ray crystal structure of the most potent inhibitor in complex with SARS-CoV-2 M^pro^, revealing critical conserved interactions responsible for its broad-spectrum activity.

## Results and discussion

### Rational design of broad-spectrum inhibitor targeting CoV M^pro^

**Figure 2.**
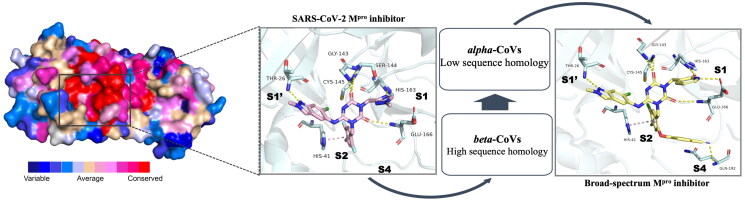
Design strategy for the development of broad-spectrum M^pro^ inhibitors.

Conservation analysis of M^pro^, as revealed through sequence alignment, demonstrates the varying degrees of conservation across the protease (as shown in Figure 2; Figure S1). Regions with lower conservation are marked in blue, while highly conserved regions are highlighted in red. Notably, the active site of M^pro^ exhibits a high degree of conservation, featuring well-defined binding pockets (S1′, S1, S2 and S3/S4). This conservation underscores M^pro^ as a promising target for the development of broad-spectrum anti-CoVs agents. Here, we introduce the concept of conserved interactions as the key noncovalent contacts between ligands and the protein backbone or conserved residues that contribute to binding stability across coronavirus M^pro^s. Our strategy for identifying broad-spectrum M^pro^ inhibitors focuses on exploring fragments capable of forming conserved interactions with M^pro^ across different CoVs. In this study, we selected **S-217622**, a potent SARS-CoV-2 M^pro^ inhibitor that interacts with the S1′, S1 and S2 pockets of the active site, as a lead compound for structural optimisation. The P1 and P2 units of **S-217622** were optimised to strengthen conserved interactions with M^pro^, aiming to enhance inhibitory potency against diverse CoV M^pro^s. The S4 pocket, characterised by its openness and inherent flexibility, presents an attractive site for optimisation. This structural feature suggests that suitable fragment act as P4 unit could be accommodated across the M^pro^ active sites of diverse CoVs. Guided by this design strategy, we introduced a series of structurally diverse fragments to occupy the S4 pocket, with the goal of improving broad-spectrum inhibitory activity by achieving compatibility with a wide range of M^pro^s.

Regarding the selection of CoVs for M^pro^ inhibitory evaluation, we prioritised those within the *beta*-CoVs genus that pose serious threats to human health and share high sequence homology with SARS-CoV-2. Subsequent evaluations were extended to *alpha-*CoVs, which exhibit lower sequence homology with SARS-CoV-2 (Figure 2).

### Chemistry

**Scheme 1.**
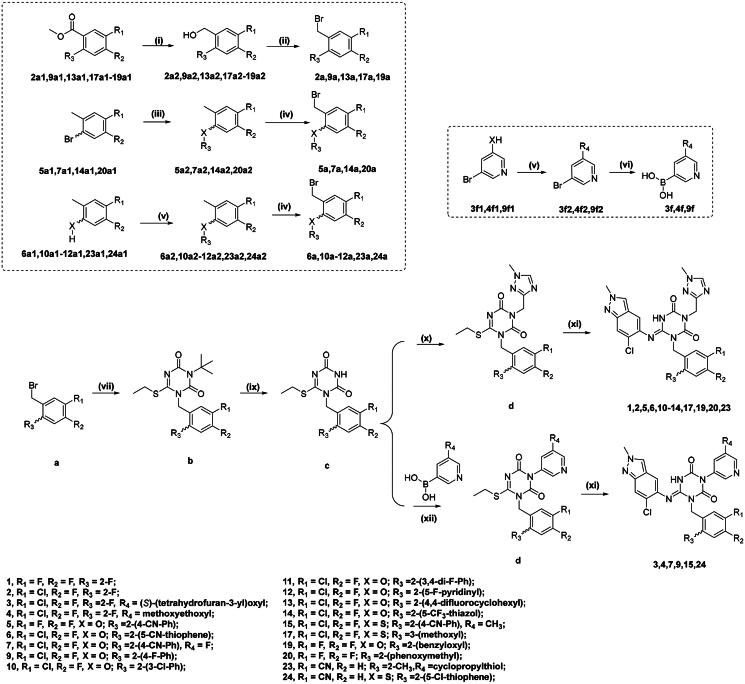
Synthetic routes of the compounds1–7, 9–15, 17,19–20, 23 and 24*^a^. ^a^*Reagents and conditions: (i) LiBH_4_, THF, 16 h; (ii) CBr_4_, PPh_3_, DCM, 0 °C-rt, 4 h; (iii) CuCl, Cs_2_CO_3_, TMHD,NMP, 120 °C, 16 h; (iv) NBS, AIBN, ACN, 80 °C, 2 h; (v) DIAD, PPh_3_, DCM, 0 °C-rt, 16 h or Cs_2_CO_3_, DMF, rt, 16 h; (vi) B_2_Pin_2_, KOAc, Pd(dppf)Cl_2_, 1,4-Dioxane, 80 °C, 16 h;(vii) K_2_CO_3_, ACN, reflux, 16 h; (ix)TFA/THF(1:1), rt, 6 h; (x) K_2_CO_3_, DMF, 60 °C, 16 h; (xi) LHMDS, 6-chloro-2-methyl-2*H*-indazol-5-amine, THF, 0 °C, 2 h. (xii) Cu(OAc)_2_, DMAP, Pyridine, 1,4-dioxane, 100 °C, 16 h.

The detailed synthetic routes of compounds **1–7**, **9–15**, **17**,**19–20**, **23** and **24** are depicted in Scheme 1. Commercially available substituted benzoates were reduced to the corresponding benzyl alcohol intermediates, which were subsequently brominated using CBr_4_ and PPh_3_ to yield the substituted benzyl bromides (**2a, 9a, 13a, 17a and 19a**). Ullmann coupling of substituted bromobenzene with various phenols or thiols afforded intermediates **5a2, 7a2, 14a2 and 20a2**. These intermediates were then treated with NBS to yield benzyl bromide derivatives. Substituted phenols or thiols underwent Mitsunobu or substitution reactions to give intermediates **6a2, 10a2–12a2, 23a2 and 24a2**. Intermediates **3f, 4f and 9f** were synthesised via the Mitsunobu and Suzuki coupling reactions. Substitution reaction and acid-mediated deprotection reactions provided intermediate **c**, which was further subjected to Chan-lam coupling with pyridine boric acid to produce compounds **3d, 4d, 7d, 9d, 15d and 24d**. The final target products were obtained by reacting these intermediates with 6-chloro-2-methyl-2*H*-indazol-5-amine.

**Scheme 2.**
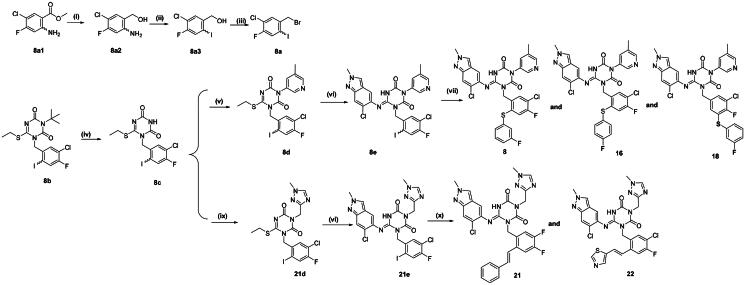
Synthetic routes of the compounds 8, 16, 18, 21 **and** 22*^a^. ^a^*Reagents and conditions: (i) LiBH_4_, THF, 16 h; (ii) concentrated HCl, NaNO_2_, acetone, 0 °C-rt, 16 h; (iii) CBr_4_, PPh_3_, DCM, 0 °C-rt, 4 h;(iv) TFA/THF(1:1), rt, 6 h; (v) Cu(OAc)_2_, DMAP, Pyridine, 1,4-dioxane, 100 °C, 16 h; (vi) LHMDS, 6-chloro-2-methyl-2*H*-indazol-5-amine, THF, 0 °C, 2 h; (vii) Substituted thiophenol, K_2_CO_3_, Cu(OAc)_2_, *o*-phenanthroline,1,4-dioxane, 110 °C, 12 h; (ix) K_2_CO_3_, DMF, 60 °C, 16 h; (x) Pd(OAc)_2_, PPh_3_, NEt_3,_ DMF, 90 °C, 16 h.

The detailed synthetic routes of compounds **8**, **16, 18, 21** and **22** are displayed in Scheme 2. Intermediate **8a2** was synthesised via reduction reaction, while compound **8a3** was prepared by Sandmeyer reaction of corresponding precursors with NaNO_2_ in acetone. Intermediates **8e** and **21e** were prepared from compound **8a** according to the procedures described in Scheme 1. Intermediate **8e** was coupled with various thiophenols via Ullmann coupling reaction under copper catalysis to afford the target compounds **8, 16 and 18.** Compounds **21** and **22** were obtained by Heck coupling reactions of **21e** with corresponding alkene derivatives.

**Scheme 3.**
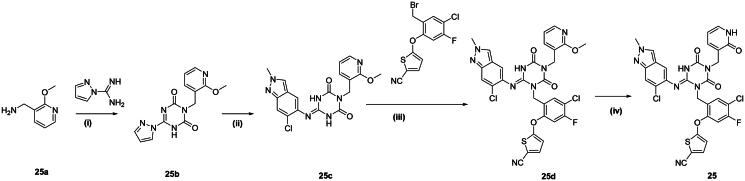
Synthetic routes of the compounds 25*^a^. ^a^*Reagents and conditions: (i) CDI, 1*H*-pyrazole-1-carboximidamide, DBU, *N*, *N*-Dimethylacetamide, 0 °C-rt, 24 h; (ii) 6-chloro-2-methyl-2*H*-indazol-5-amine, PTSA, NMP, 80 °C, 1 h; (iii) DIPEA, *N*, *N*-Dimethylacetamide, 60 °C, 3 h; (iv) NaI, TMSCl, ACN, 65 °C, 1 h.

The synthetic route for compound **25** is shown in Scheme 3. Cyclisation of **25a** with 1*H*-pyrazole-1-carboximidamide, followed by a substitution reaction, provided intermediate **25c**. Subsequent reaction with **6a** afforded compound **25d**, which was demethylated using NaI and TMSC to yield the final product, compound **25.**

### High-risk beta-CoVs m^pro^ inhibitory activities evaluations and structure–activity relationship

Based on rational design analysis, we first optimised P1, P2 and P1’ units, followed by introduction of fragments extend from P2 unit into the S4 pocket, forming P4 unit aimed at enhancing broad-spectrum activity. At the time this study was conducted, no comprehensive catalogue of high-risk coronaviruses had been established. Therefore, we selected six CoVs closely related to SARS-CoV-2 and MERS-CoV as representative CoV M^pro^ (Figure S2) for our investigation. The inhibitory activities of all the synthesised compounds were evaluated by determining their IC_50_ values based on a FRET-based assay according to a previously established method^34^. The M^pro^ inhibitory activities of compounds **1–8** against six *beta*-CoVs were presented in Table 2.

**Table 1.** M^pro^ inhibitory activities of reported compounds against CoVs.

PF-07321332 (Nirmatrelvir)/IC_50_ (μM)		S-217622 (Ensitrelvir)/IC_50_ (μM)	
**SARS-CoV-2**	0.016	**SARS-CoV-2**	0.017
**SARS-CoV-1**	0.01	**SARS-CoV-1**	0.015
**HCoV-OC43**	0.07	**HCoV-OC43**	0.269
**MERS-CoV**	0.048	**MERS-CoV**	0.527
**HCoV-HKU1**	0.009	**HCoV-HKU1**	0.010
**HCoV-229E**	0.235	**HCoV-229E**	>10
**HCoV-NL63**	0.234	**HCoV-NL63**	>10
PF-00835231 /Ki (nM)		VMM-15 /IC_50_ (μM)	
**SARS-CoV-2**	0.27	**SARS-CoV-2**	0.09
**HCoV-OC43**	0.51	**SARS-CoV-1**	0.33
**HCoV-HKU1**	0.85	**HCoV-OC43**	0.34
**HCoV-229E**	1.5	**HCoV-HKU1**	0.22
**HCoV-NL63**	0.77	**HCoV-229E**	0.06
**PEDV**	0.3	**HCoV-NL63**	0.44
**FIPV**	0.12	**PEDV**	0.05
**HKU4-CoV**	0.034	**HKU4-CoV**	0.07
**HKU5-CoV**	0.033	**WS-CoV**	0.04
**HKU9-CoV**	0.74	**LR-CoV**	28
**MHV-CoV**	1.2	**UU23**	0.66
**IBV-CoV**	4.0	–	–

**Table 2.** M^pro^ inhibitory activities of compounds 1–8 against six *beta*-CoVs^a^.

Compound	Structure	M^pro^ IC_50_ (μM)*^a^*					
		SARS CoV-2	RS4231	RS4081	RATG13	MjHKU4r-CoV-1	BANAL-236
**PF-07321332**		0.023	0.023	0.010	0.031	0.031	0.046
**S-217622** **(1)**	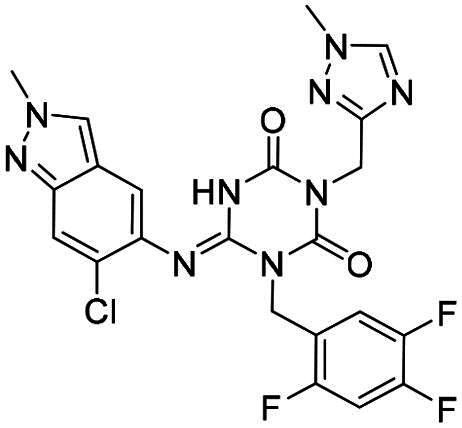	0.048	0.067	0.172	0.026	0.172	0.086
**2**	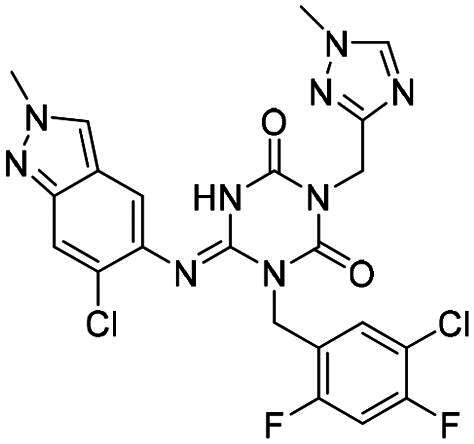	0.058	0.082	0.064	0.161	0.161	0.123
**3**	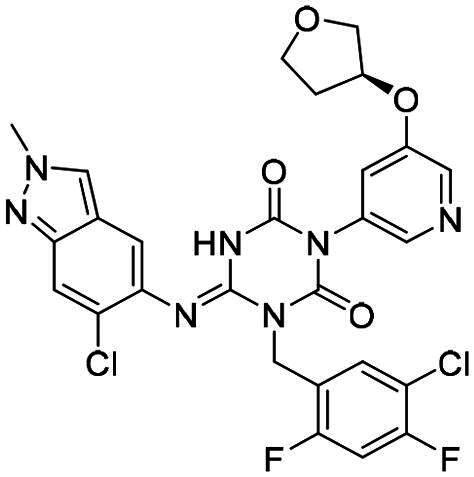	0.026	0.030	0.046	0.030	1.211	0.072
**4**	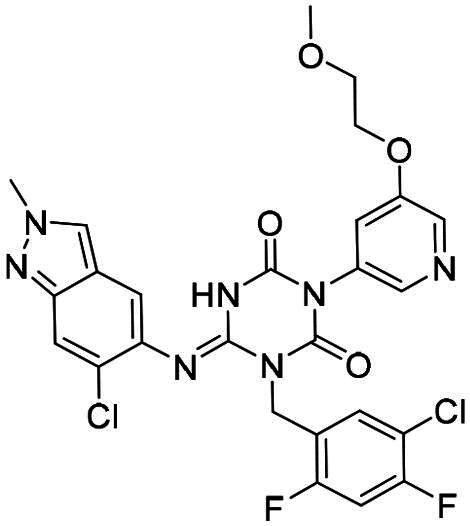	0.021	0.039	0.023	0.123	1.018	0.073
**5**	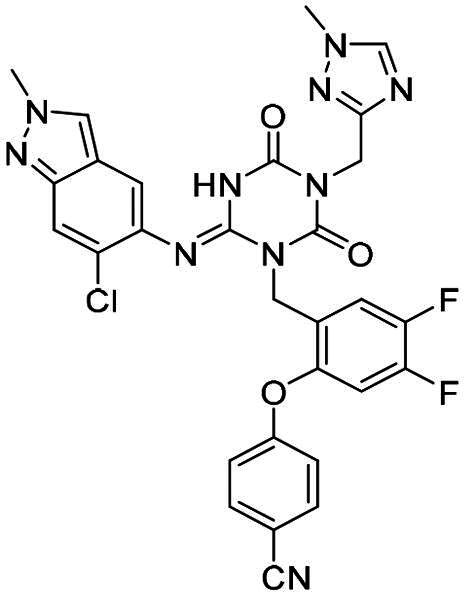	0.012	0.078	0.088	0.106	0.326	0.115
**6**	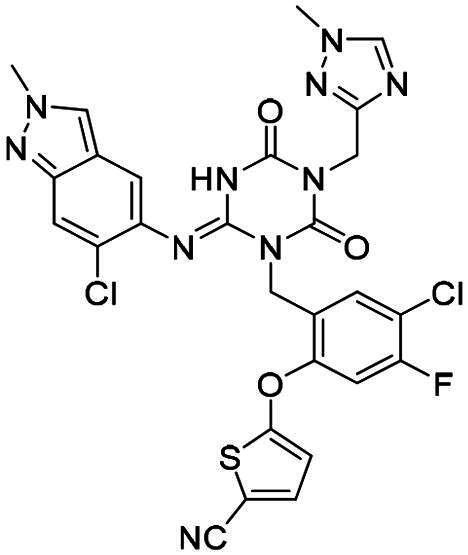	0.016	0.020	0.050	0.060	0.039	0.013
**7**	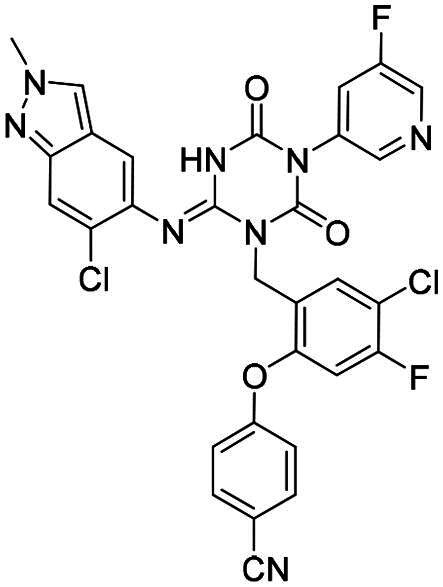	0.051	0.092	0.076	0.122	1.180	0.135
**8**	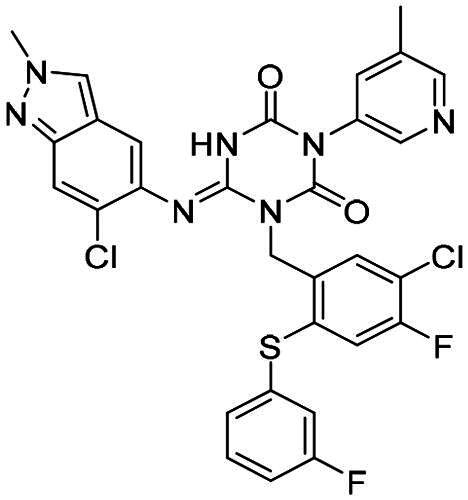	0.010	0.028	0.020	0.054	0.034	0.027

^a^All values are expressed as the mean from three experiments.

As shown as Table 2, **S-217622** exhibited potent broad-spectrum M^pro^ inhibitory activity against the *beta*-CoVs listed above, demonstrating similar potency against evaluated CoV M^pro^s as that against SARS-CoV-2 M^pro^, although with a slightly reduction in potency against RS4081 and MjHKU4r-CoV-1.

In the S2 pocket, trifluorobenzylic moiety of P2 unit occupied the hydrophobic S2 pocket, interacting with the side chain of His41. To enhance the hydrophobic interactions, we replaced the fluorine atom with chlorine, thereby increasing the lipophilicity of the P2 unit to obtain compound **2**. While compound **2** exhibited an approximately 8-fold and 2-fold decrease in potency against RATG13 and BANAL-236, respectively, it showed a 3-fold improvement against RS4231 and maintained activity against SARS-CoV-2 and MjHKU4r-CoV-1 relative to **S-217622**.

In the S1 pocket, hydrogen bonding between the amide-containing P1 unit of **S-217622** and His163 was identified as critical for potency. To optimise this interaction, we replaced the triazole group with a pyridine moiety to maintain hydrogen bonding while reducing the flexibility of the P1 unit. Compounds **3** and **4**, resulting from this modification, exhibited improved M^pro^ inhibitory activity against all tested CoVs except MjHKU4r-CoV-1. Compound **3** demonstrated IC_50_ values below 0.1 μM for all CoVs except MjHKU4r-CoV-1.

The S4 pocket, characterised by its flexibility and openness, offers a promising opportunity to establish favourable van der Waals or hydrophobic interactions across diverse CoVs by introducing fragments capable of accommodating the S4 pocket of M^pro^. The P2 unit is stabilised by hydrophobic interactions within the S2 pocket, where the substituted phenyl ring engages in π–π interactions with His41. This structural feature positions it as an appropriate scaffold for extension into the P4 unit, facilitating effective occupation of the S4 pocket. To explore this strategy, aromatic ether fragments were introduced at the 2-position of the P2 unit, yielding compounds **5** and **6**. Both compounds displayed potent M^pro^ inhibitory activity against six CoVs, with compound **6** demonstrating IC_50_ values below 0.1 μM across all six CoVs. These findings demonstrated the feasibility of targeting the S3/S4 pocket to achieve broad-spectrum M^pro^ inhibition.

Building on the success of P4 optimisation, we conducted a combinatorial optimisation of both P1 and P4 units to obtain compounds **7** and **8**. Compound **7** exhibited reduced activity against MjHKU4r-CoV-1 compared to compound **5**. In contrast, compound **8**, featuring a 3-fluorophenyl ring linked to a thioether, demonstrated potent M^pro^ inhibitory activity against all six CoVs, with IC_50_ values below 0.06 μM. These findings highlight the critical role of optimising both the S1 and S4 pockets to achieving broad-spectrum M^pro^ inhibitory activity against CoVs. Incorporating suitable fragments into the S4 pocket significantly enhanced the conserved interactions of compounds with M^pro^ across diverse *beta*-CoVs.

### High-risk alpha-CoVs M^pro^ inhibitory activities evaluations and structure–activity relationship

**Figure 3.**
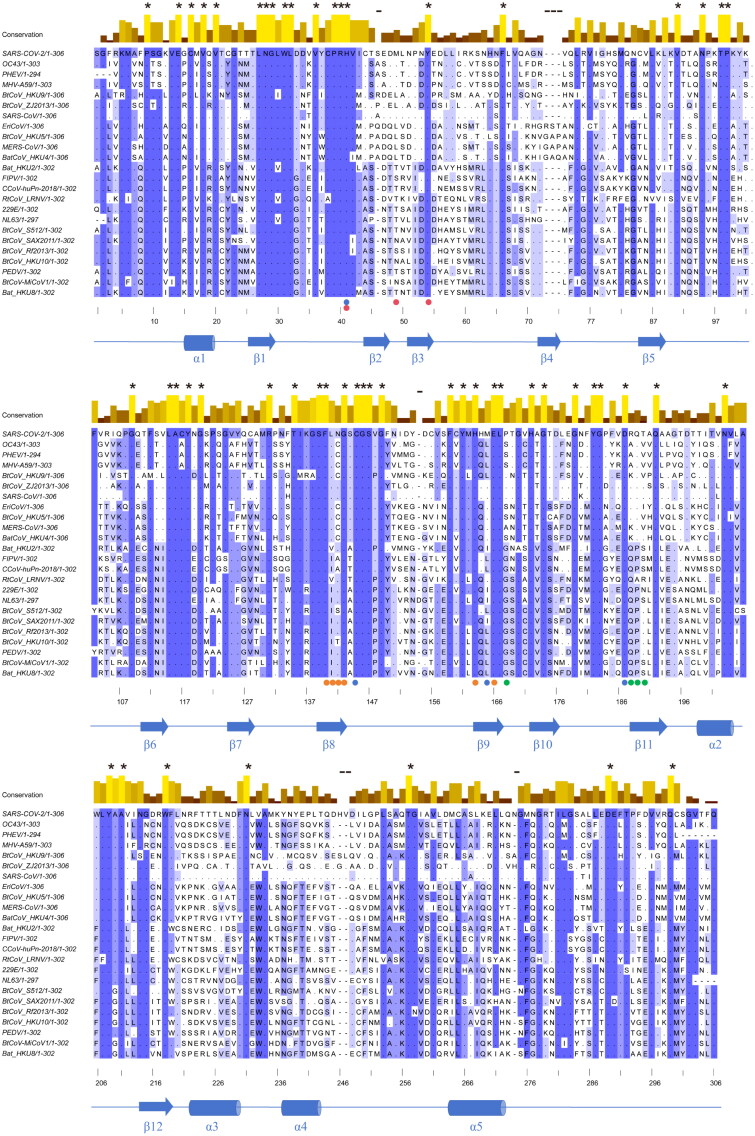
Amino acid homologous sequence alignment for 24 reported high-risk coronaviruses the main protease (M^pro^). Using Jalview software, select the ClustalW algorithm to align the 24 selected sequences, and then use the Shannon Entropy algorithm to score the conservation. Key amino acids in the binding pockets was indicated by dots (orange: S1 pocket; blue: S1’ pocket; red: S2 pocket; green: S3/S4 pocket).

Based on the evaluation of compounds against the M^pro^ of the above six *beta*-CoVs, our focus expanded to *alpha*-CoVs. Around this time, virologists proposed a list of 20 high-risk CoVs, which provided invaluable guidance for our research. Inspired by this concept, we recognised that developing broad-spectrum M^pro^ inhibitors against high-risk CoVs represents a strategic approach to prepare for future CoV pandemics. Accordingly, we incorporated these 20 high-risk CoVs into our study, along with additional CoVs including PHEV, FIPV, and PEDV, which are associated with severe infections in felidae and suidae, as well as SARS-CoV-2. We performed amino acid homologous sequence alignment of M^pro^ from 24 reported high-risk CoVs (Table S1). The ClustalW algorithm in Jalview software was used to align the selected sequences, and the Shannon Entropy algorithm was subsequently applied to evaluate the conservation, as shown in Figure 3. This analysis provided valuable insights into the sequence homology among these CoVs and served as the basis for selecting representative CoVs to assess broad-spectrum activity.

Based on the above analysis, the 13 high-risk *alpha-*CoVs were primarily classified into four categories (Figure S1). We proposed selecting representative *alpha-*CoVs based on sequence homology to evaluate the broad-spectrum M^pro^ inhibitory activity of the compounds that had demonstrated effectiveness against the M^pro^ of *beta*-CoVs. Accordingly, HKU2, LRNV, SAX2011, and PEDV (Figure S2) were selected as representative *alpha-*CoVs for evaluation. We first assessed the M^pro^ inhibitory activity of **S-217622** and previously optimised compounds targeting the S1 and S4 pockets against these four *alpha-*CoVs. This evaluation aimed to determine the cross-reactive potential of these compounds in inhibiting M^pro^ activityacross both *beta* and *alpha-*CoVs.

As depicted in Table 3, the results demonstrated that **S-217622** exhibited significantly reduced M^pro^ inhibitory activity against all four *alpha-*CoVs, with an IC_50_ of 2.20 μM for PEDV and values exceeding 10 μM for the remaining three *alpha-*CoVs, demonstrating markedly weaker activity compared to its performance against *beta*-CoVs. Compound **3** showed modest improvement in M^pro^ inhibitory activity against LRNV and PEDV compared to **S-217622**. Compound **6**, which exhibited promising M^pro^ inhibitory activity against *beta*-CoVs, established potent M^pro^ inhibitory against four *alpha-*CoVs but displayed variable activity.

**Table 3.** M^pro^ inhibitory activities of compounds 1, 3, 6 against four representative *alpha*-CoVs^a^.

Compound	Structure	M^pro^ IC_50_ (μM)*^a^*			
		HKU2	LRNV	SAX2011	PEDV
**PF-07321332**		0.58	0.43	0.38	0.50
**S-217622** **(1)**	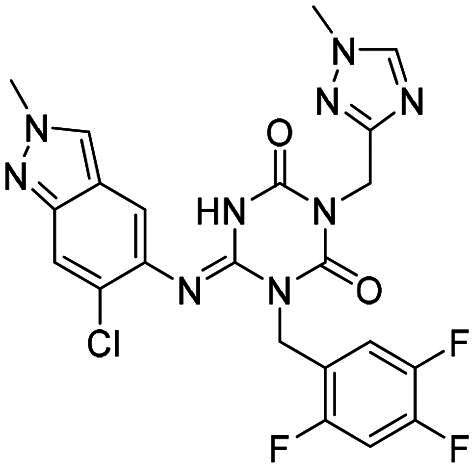	>10	>10	>10	2.20
**3**	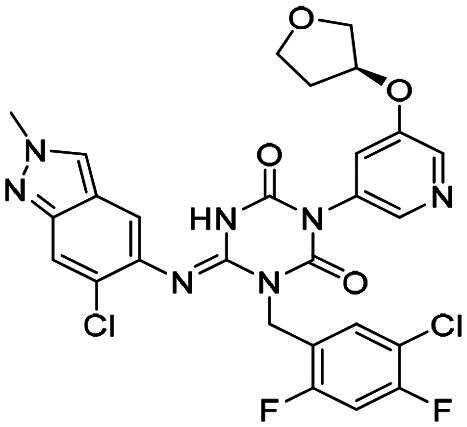	>10	3.1	>10	0.040
**6**	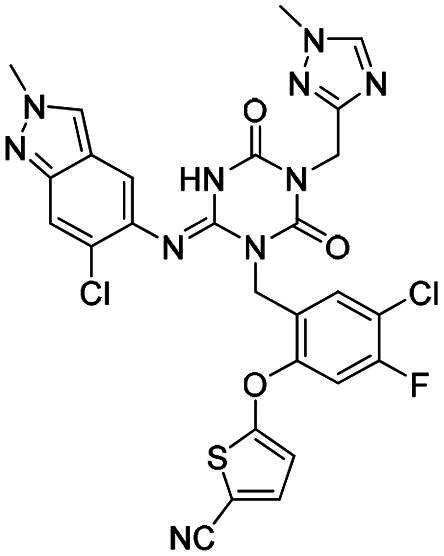	3.65	1.47	0.05	0.19

^a^All values are expressed as the mean from three experiments.

These results preliminarily suggest that occupying the S4 pocket is beneficial for the broad-spectrum M^pro^ inhibitory activity. To further confirm this hypothesis, we performed a range of structural modifications targeting the S4 pocket across different CoV M^pro^s, including introducing straight-chain fragments, aliphatic rings, five-membered aromatic rings, and six-membered aromatic rings. Accordingly, a series of compounds **9–22** were designed and synthesised.

As presented in Table 4, compounds **9–11,** bearing substituted phenyl groups in the P4 units linked at 2-position of phenyl ring in the P2 unit via an ether bond, exhibited comparable broad-spectrum M^pro^ inhibitory activity, with the micromolar activity against three CoVs (HKU2, LRNV and SAX2011) and submicromolar inhibition against PEDV. Compound **12**, bearing a pyridine ring in the P4 unit, demonstrated M^pro^ inhibitory activity comparable to compound **9**. In contrast, compound **13**, which incorporates an alicyclic ring instead of an aromatic ring, displayed reduced broad-spectrum M^pro^ inhibitory activity, with extremely weak activity against HKU2 and LRNV. The loss of activity possibly attributed to the inability of the alicyclic ring to fit into the S4 pocket of diverse M^pro^s due to its steric constraints, in contrast to aromatic fragments such as the five-membered aromatic ring (compound **6**) and six-membered aromatic rings (compounds **9–12**). To confirm this speculation, compound **14**, incorporating a smaller alicyclic ring, was synthesised. It exhibited superior broad-spectrum M^pro^ inhibitory activity compared to compounds **6** and **9–12**, with IC_50_ values below 0.3 μM across the selected *alpha-*CoVs. These results further highlight the critical role of occupying S3/S4 pocket in enhancing broad-spectrum M^pro^ inhibitory activity. Compounds **15**–**16**, derived from compound **8**, exhibited enhanced M^pro^ inhibitory activity against *alpha*-CoVs compared to compounds **9–12**, indicating that the P1 unit of this scaffold plays a crucial role in broad-spectrum inhibitory activity. Compound **17** and **18**, with P4 units attached to the 3-position of the phenyl ring in the P2 unit, exhibited significantly reduced M^pro^ inhibitory activity against HKU2, LRNV and SAX2011, while maintained activity compared to **S-217622** against SARS-CoV-2 and PEDV. These findings suggest that the 3-position linkage of the phenyl ring is suboptimal for effective P4 unit extend into the S3/S4 pocket, underscoring the importance of the spatial orientation and position of the phenyl linkage for broad-spectrum M^pro^ inhibitory activity.

**Table 4.** M^pro^ inhibitory activities of compounds 9–22 against SARS-CoV-2 and four representative *alpha-*CoVs^a^.

Compound	Structure	M^pro^ IC_50_ (μM)*^a^*				
		SARS-CoV-2	HKU2	LRNV	SAX2011	PEDV
**9**	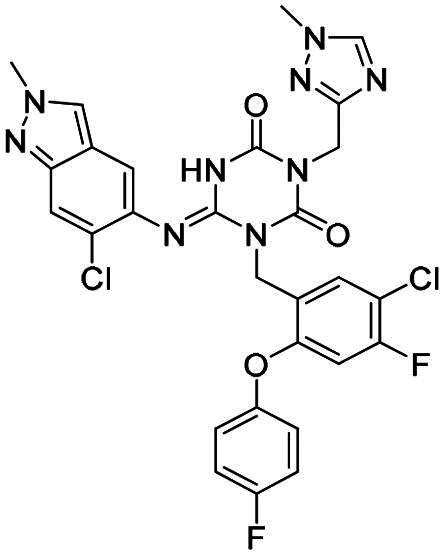	0.016	6.43	5.94	8.90	0.80
**10**	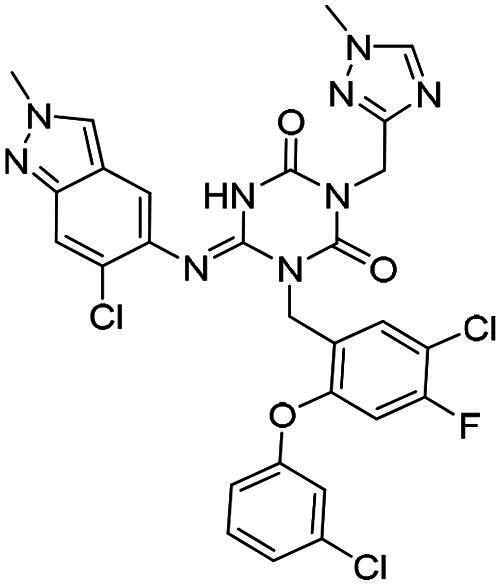	0.012	6.45	3.72	3.97	0.46
**11**	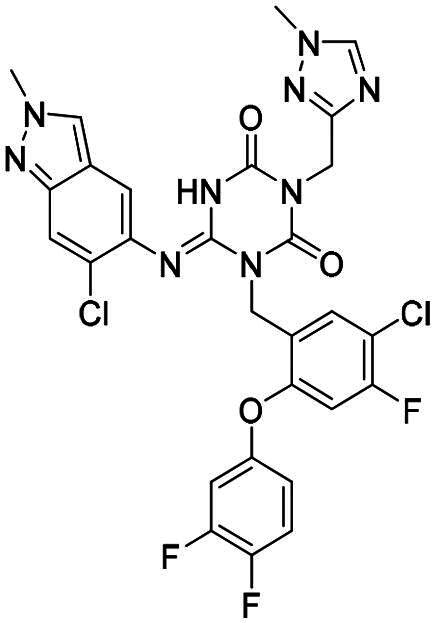	0.010	3.89	2.79	3.79	0.34
**12**	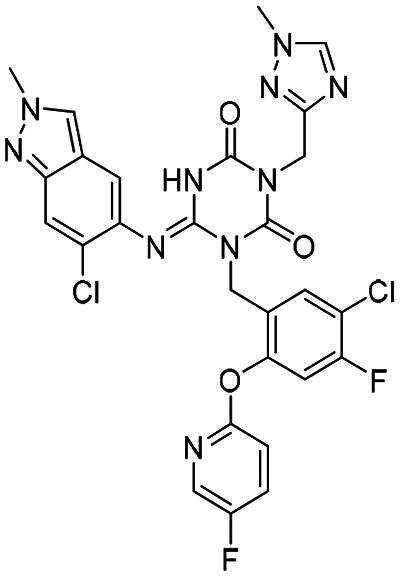	0.018	7.30	4.49	5.92	0.41
**13**	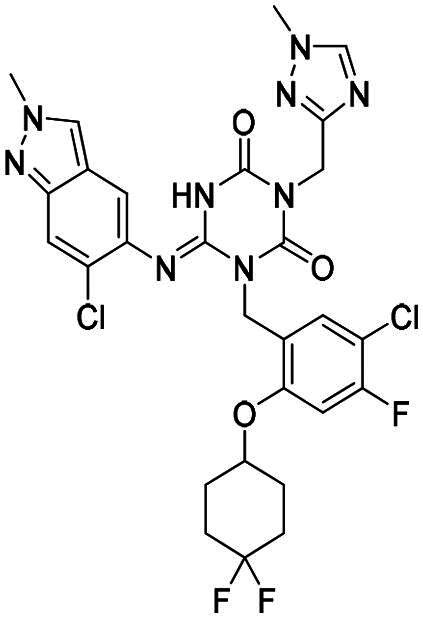	0.016	>10	>10	3.97	3.20
**14**	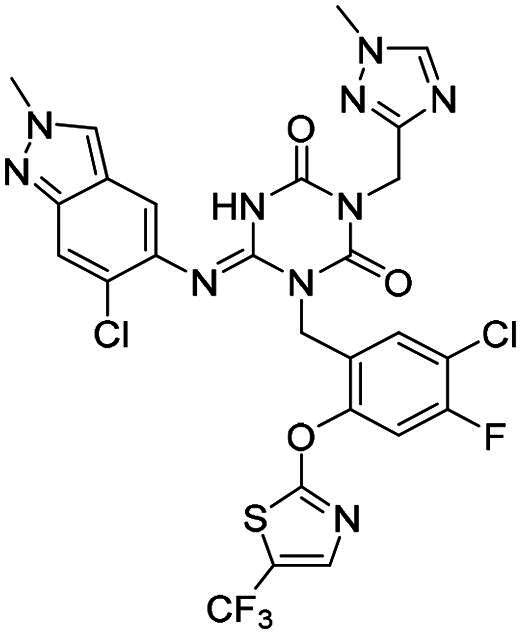	0.012	0.27	0.12	0.16	0.12
**15**	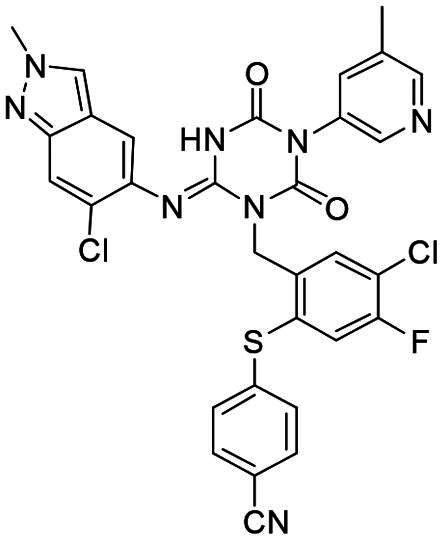	0.042	0.93	0.43	1.45	0.11
**16**	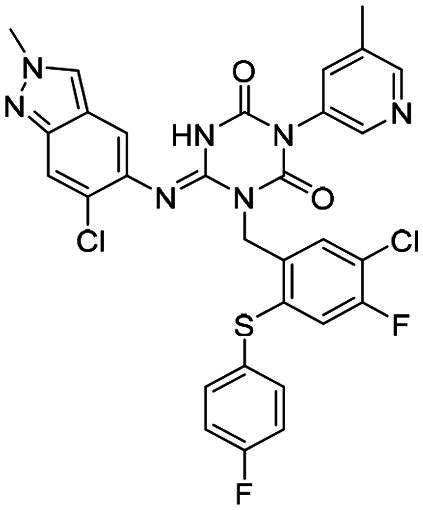	0.043	4.13	1.48	>10	0.62
**17**	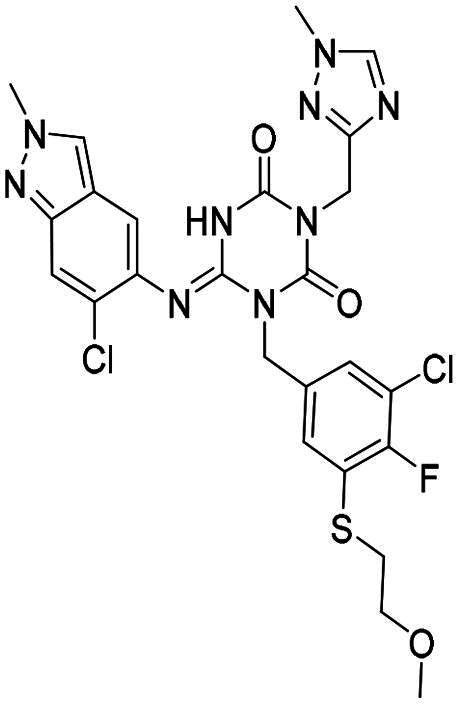	0.025	>10	>10	>10	2.49
**18**	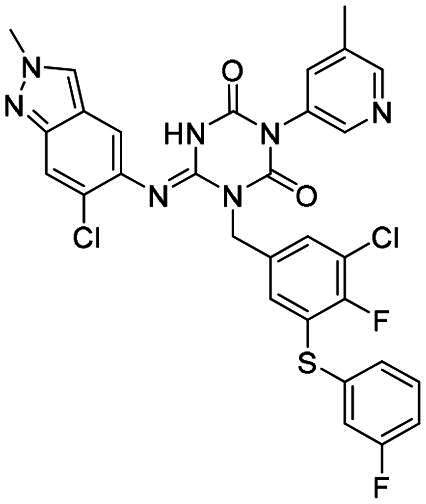	0.011	>10	>10	>10	2.44
**19**	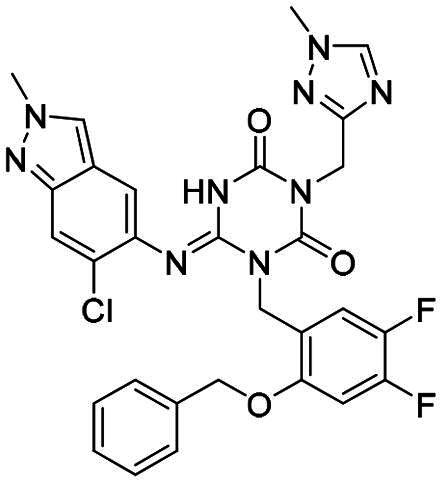	0.046	8.33	>10	>10	>10
**20**	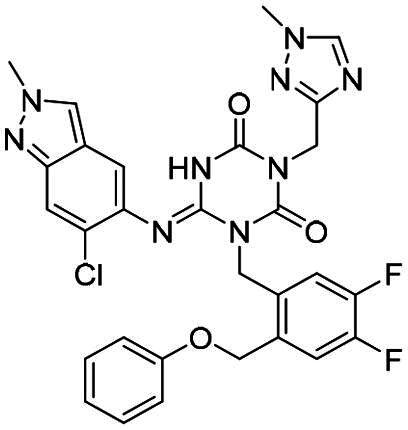	0.075	>10	>10	>10	4.41
**21**	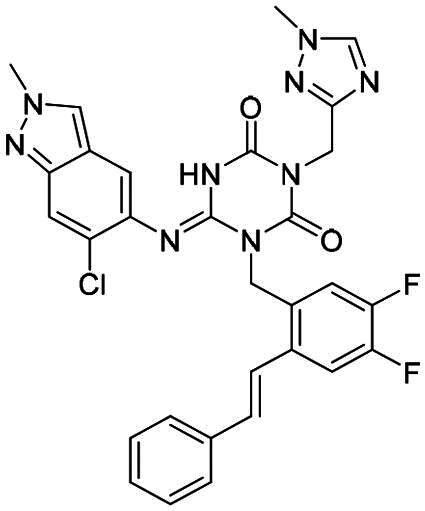	0.049	>10	4.27	>10	1.99
**22**	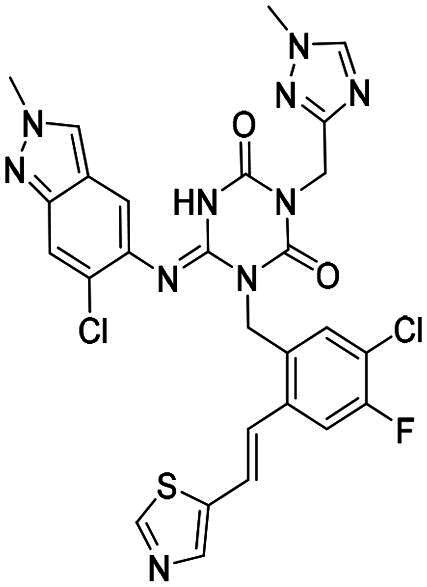	0.030	9.06	3.21	4.34	0.21

^a^All values are expressed as the mean from three experiments.

Efforts to further optimisation involved exploring linkages between the P2 and P4 units using two-atom connectors, such as –OCH_2_–, –CH_2_O– and –CH_2_=CH_2_– linkers. However, these linkages did not lead to improved activity, with compounds **19**–**22** showing significant reductions in M^pro^ inhibitory activity, except for SARS-CoV-2. Specifically, the **–**OCH_2_– and –CH_2_O– linkers resulted in complete loss of M^pro^ inhibitory activity across most *alpha-*CoVs. Compound **22**, incorporating a five-membered aromatic ring, demonstrated superior M^pro^ inhibitory activity compared to compound **21**, suggesting that a five-membered aromatic structure is more appropriate for occupying the S4 pocket compared to six-membered aromatic counterparts. These findings underscore the critical role of achieving effective occupancy of the S4 pocket.

Consistent with our previous conjecture, the results of optimisation showed improved M^pro^ activity of four *alpha*-CoVs, further verifying the importance of occupying the S1, S2, and S4 pocket fragments, leading to the identification of three promising compounds (Table 4). Compound **23**, with a modified P2 unit, demonstrated potent M^pro^ inhibitory activity against representative *alpha-*CoVs, indicating that appropriately designed modifications in the P2 unit could significantly improve broad-spectrum activity. Compound **24**, a derivative of compound **23**, exhibited better broad-spectrum M^pro^ inhibitory effects, with IC_50_ values below 0.3 μM against all four *alpha-*CoVs. This observation further confirmed the critical role of S4 pocket occupancy in enhancing broad-spectrum M^pro^ inhibitory activity. Notably, compound **25** exhibited potent M^pro^ inhibitory across four *alpha*-CoVs M^pro^, with IC_50_ values below 0.6 μM, including remarkable activity against PEDV at 0.07 μM (Table 4). The finding revealed that the interaction with S1 pocket was also important for broad-spectrum M^pro^ inhibitory activity.

### Further evaluations of broad-spectrum inhibitory activities

To further evaluate the broad-spectrum M^pro^ inhibitory activities of our compounds, we selected four structurally diverse compounds (**6**, **12**, **15**, and **25**) with robust broad-spectrum M^pro^ inhibitory activity across diverse structural types for further evaluation against additional CoVs. Based on clustering diagram shown in Figure 3, the 24 high-risk CoVs were primarily classified into eight categories. The selection of representative CoVs from each category for activity evaluation was guided by criteria that favoured assessing broad-spectrum inhibitory activity. As a result, we chose SARS-CoV-1, Eri, FIPV, HKU10, and HKU8 (Figure S2) for further evaluation (Tables 5 and 6).

**Table 5.** M^pro^ inhibitory activities of compounds 23–25 against SARS-CoV-2 and four representative *alpha-*CoVs^a^.

Compound	Structure	M^pro^ IC_50_ (μM)*^a^*				
		SARS-CoV-2	HKU2	LRNV	SAX2011	PEDV
**23**	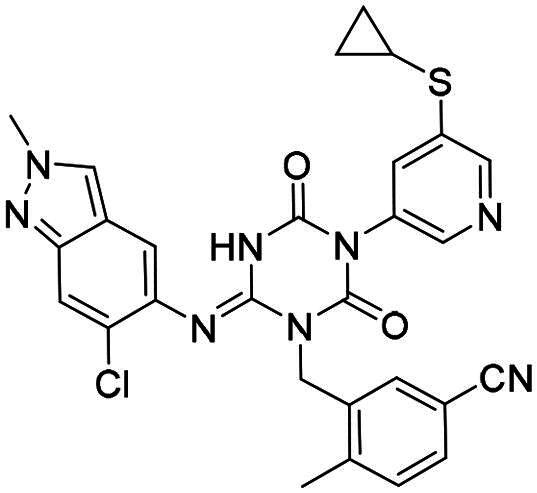	0.010	1.90	0.13	0.43	0.17
**24**	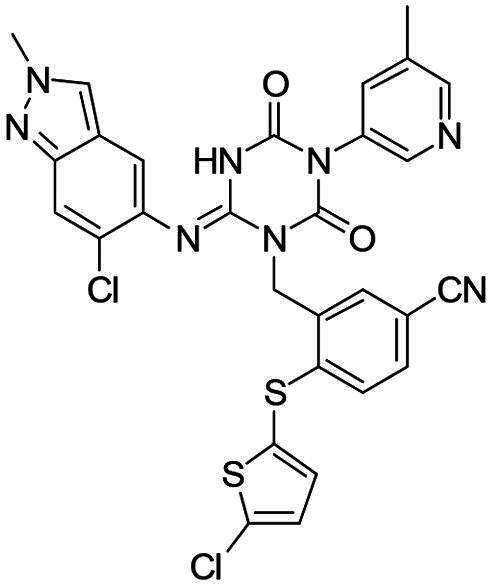	0.012	0.27	0.12	0.16	0.12
**25**	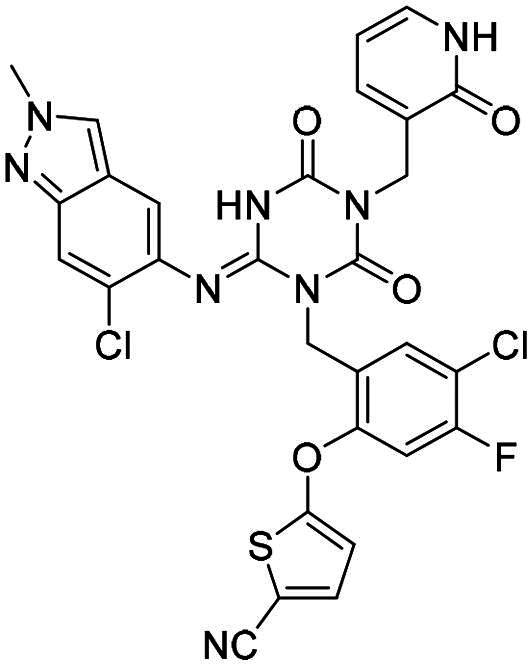	0.013	0.56	0.45	0.34	0.07

^a^All values are expressed as the mean from three experiments.

**Table 6.** Further evaluation of broad-spectrum M^pro^ inhibitory activity^a^.

Compound	Structure	M^pro^ IC_50_ (μM)*^a^*				
		SARS-CoV-1	Eri	FIPV	HKU10	HKU8
**PF-07321332**		0.022	0.013	0.322	0.086	0.35
**S-217622** **(1)**	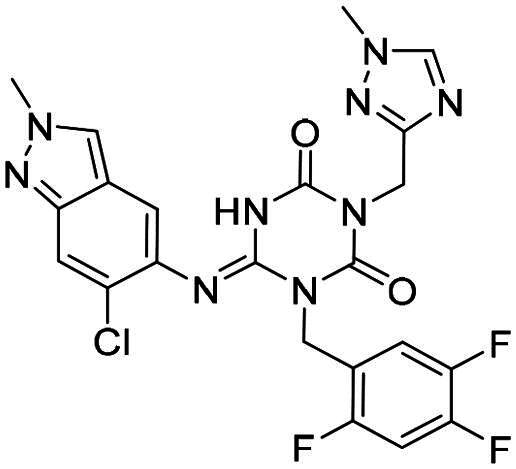	0.045	0.018	9.37	1.22	2.07
**6**	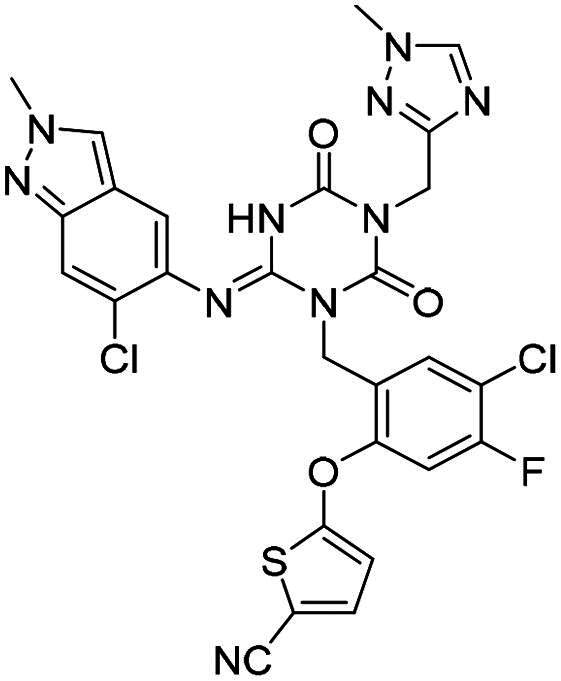	0.021	0.011	1.01	0.026	0.06
**12**	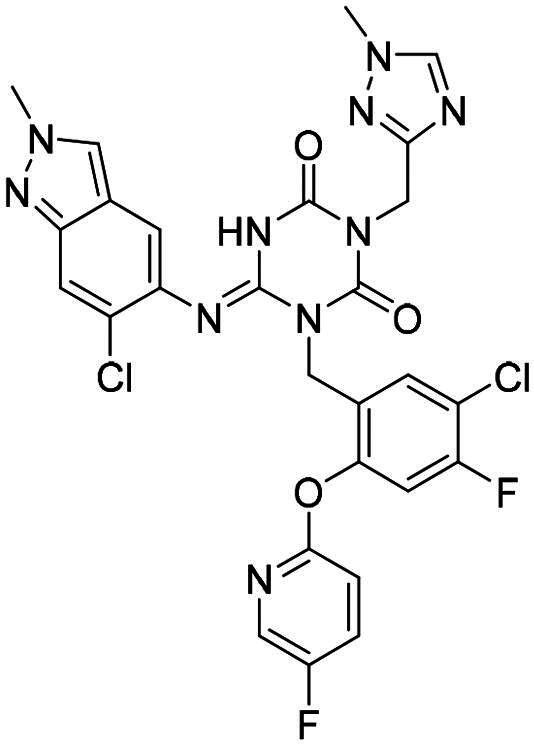	0.064	0.051	5.54	0.15	0.18
**15**	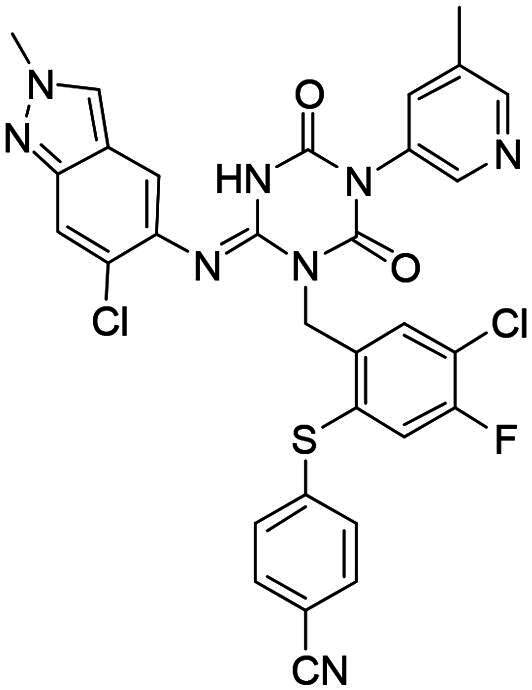	0.072	0.068	1.44	0.042	0.024
**25**	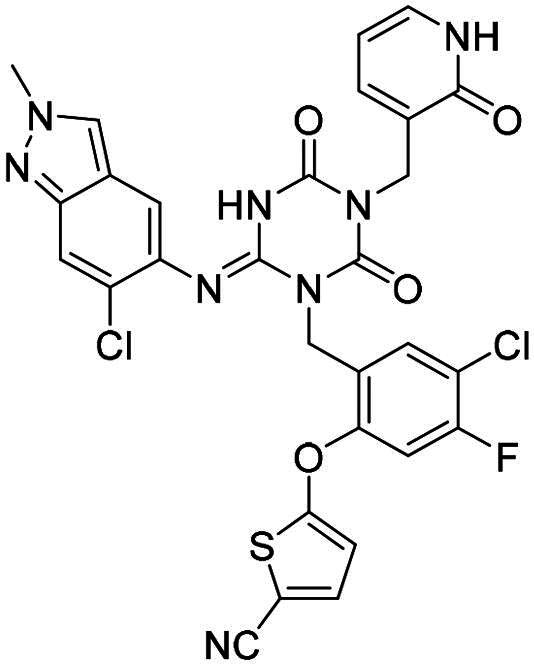	0.025	0.011	0.216	0.016	0.018

^a^All values are expressed as the mean from three experiments.

Compounds **6** and **12** demonstrated superior broad-spectrum M^pro^ inhibitory activity against ten CoVs compared to **S-217622**, providing further validation of the design strategy focused on targeting the S4 pocket. Compound **6**, featuring a five-membered aromatic ring in the P4 unit, exhibited stronger M^pro^ inhibitory activity against three *alpha-*CoVs and two *beta*-CoVs compared to compound **12**, which contains a six-membered aromatic ring in the P4 unit. This trend is consistent with the M^pro^ inhibitory activity observed against the previously tested four *alpha-*CoVs, reinforcing the conclusion that a five-membered aromatic ring is better suited for occupying the S4 pocket. Among these, Compound **25**, which initially exhibited the most promising performance against the ten tested CoV M^pro^s, continued to demonstrate remarkable broad-spectrum inhibitory activity.

In summary, substitution of the methyl-pyrazole moiety with a 3-substituted pyridine was found to be favourable for occupying the S1 pocket, as this modification not only preserved inhibitory activity against M^pro^ but also improved molecular rigidity and facilitated hydrogen bond formation with His163. For the S2 pocket, the introduction of a hydrophobic group was required to ensure proper occupancy, with halogen atoms being particularly advantageous; additionally, potential π–π stacking interaction with His41 was considered. Moreover, extending substituents from the appropriate position of the S2-occupying moiety efficiently reached the S3/S4 pockets. Compared to six-membered rings, five-membered rings exhibited a more favourable spatial fit within the S3/S4 pockets and offered the potential to establish hydrogen bonds with Gln192, which was beneficial for enhancing broad-spectrum inhibitory activity.

### *The crystal structure of compound 25 in complex with SARS*–*CoV-2 M^pro^*

**Figure 4.**
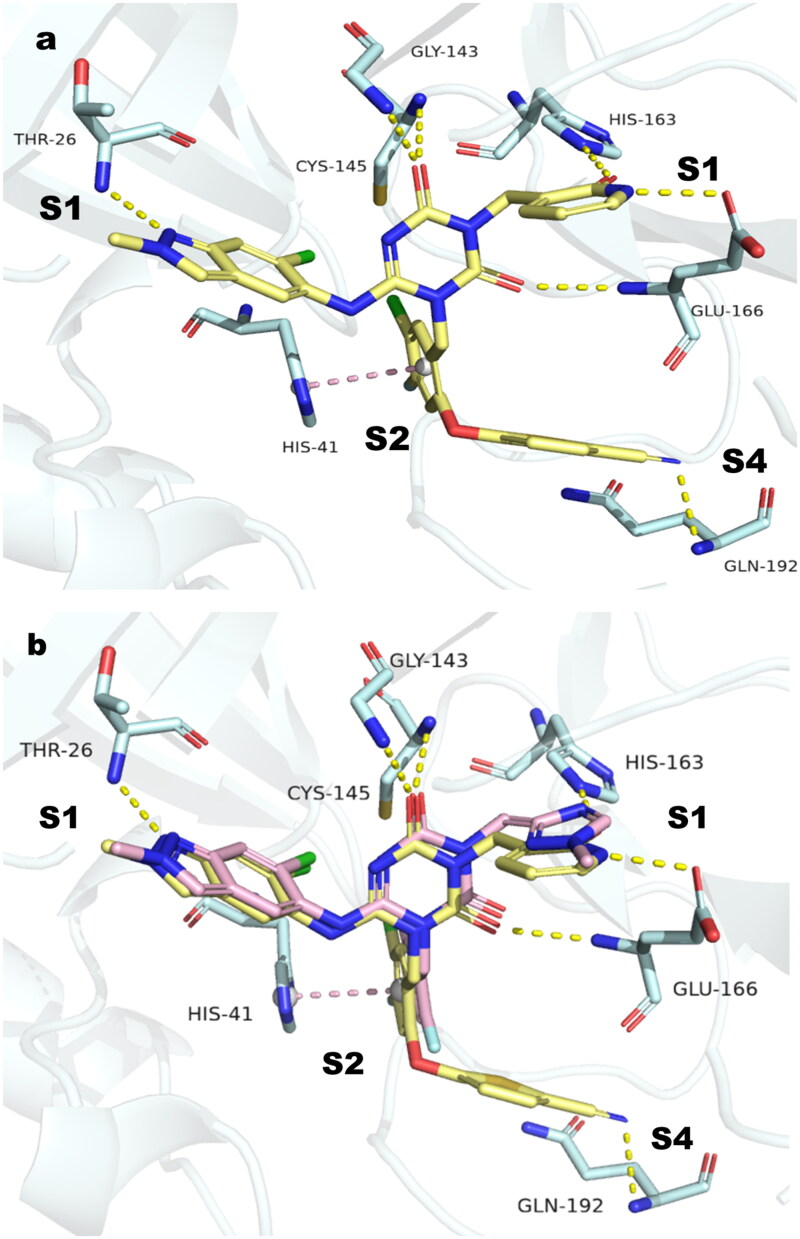
(a) The X-ray crystal structure of SARS–CoV-2 M^pro^ in complex with compound **25** (PDB: 9LLL); (b) Overlay of compound **25** with **S-217622** (pink color). Hydrogen bonds are depicted with yellow dashed lines, while π–π interactions are shown with pink dashed lines.

We resolved the X-ray cocrystal structure of compound **25** bound to SARS–CoV-2 M^pro^ and the binding mode was shown in Figure 4a. Data collection and refinement statistics for the inhibitor-M^pro^ crystal structures was summarised in Table 7. In the S2 pocket, the substituted phenyl ring of compound **25** well occupies the hydrophobic cavity, engaging in a π–π stacking interaction with His41. In the S1′ pocket, indazole group forms a hydrogen bond with the main-chain NH of Thr26. The central scaffold of compound **25** is also important for the interaction, with the 2-carbonyl oxygen forming a hydrogen bond with the main-chain NH of Glu166 and the 4-carbonyl oxygen establishing hydrogen bonds with the NH groups of Gly143 and Cys145. These interactions are similar to those observed with **S-217622**. It is worth noting that, compound **25** also possesses distinct and enhanced binding features. In the S1 pocket, the six-membered ring establishes hydrogen bonds with the side chain of His163 and the main-chain NH of Glu166. The cyano-thiophene fragment of compound **25** fits well in the S4 pocket, a region not occupied by **S-217622** (Figure 4b). These distinguishing binding characteristics of compound **25**, particularly the additional hydrogen bonding interactions and sufficient S4 pocket occupation, underscore its enhanced inhibitory activity.

**Table 7.** Data collection and refinement statistics for the inhibitor-M^pro^ crystal structures.

	SARS–CoV-2 M^pro^-**25**
**PDB code**	9LLL
**Data collection**	
Space group	I 1 2 1
Cell dimensions	
*a, b, c* (Å)	51.67, 84.07, 88.50
α, β, γ (°)	90.00, 96.01, 90.00
Wavelength (Å)	1.54184
Resolution(Å)	18.96 − 2.40
Completeness	99.9% (18.96–2.40)
*R*_merge_ (%)^a,b^	
*I*/σ^a^	2.22
**Refinement**	
Resolution (Å)	18.96–2.40
No. of reflections	49950
*R*_work_/*R*_free_	0.235/0.282
**B factor (Å^2^)**	
Protein	25.72
Water	21.71
**RMSD**	
Bond length (Å)	0.009
Bond angles (°)	1.032
**Ramachandran plot (%)**	
Favoured	97
Allowed	3
Outliers	0

## Conclusion

In this study, we presented a systematic approach for the development of broad-spectrum inhibitors targeting high-risk CoVs, with a focus on conserved interactions within the M^pro^s active site. Our findings underscored the potential of utilising the conserved interactions to enhance the broad-spectrum inhibition. Originally, we proposed a list of 24 high-risk CoVs, clustered based on sequence homology. Our strategy prioritised the optimisation of M^pro^ inhibitors against *beta*-CoVs closely related to SARS-CoV-2, followed by the exploration of more distantly related with *alpha-*CoVs. By focusing on the high-risk CoVs, we aimed to identify M^pro^ inhibitors with broad applicability against potential future emerging CoVs. Notably, our broad-spectrum M^pro^ inhibitors were designed with consideration of the CoVs that pose the greatest threat to human health, making them highly likely to be effective against novel or future CoVs.

Furthermore, we demonstrated the feasibility of designing broad-spectrum inhibitors by targeting the highly conserved M^pro^ active site. Specifically, we confirmed that enhancing conserved interactions across CoV M^pro^s significantly improves the M^pro^ inhibitory potency against a broad spectrum of CoVs. To optimise broad-spectrum efficacy, we proposed strategies such as introducing conserved ionic bonds, hydrogen bonds, and π–π stacking interactions to enhance binding affinity against M^pro^s. These interactions should be made with conserved units, such as the protein backbone or conserved amino acid side chains. Additionally, optimal occupation of binding pockets by appropriately shaped and sized molecular groups is crucial, with particular attention to the orientation of the molecular scaffold and linker length.

In our study, we optimised the lead compound **S-217622** by introducing a hydrogen bond with Gln166, van der Waals and hydrophobic interactions with the S3/4 pocket, and enhancing hydrophobic interactions with the S2 pocket. These conserved interactions, which were validated by structure–activity relationship (SAR) studies, confer broad-spectrum M^pro^ inhibitory against various CoVs. Notably, the incorporation of a five-membered aromatic ring to occupy the S3/4 pocket led to improve interactions across ten CoV M^pro^s evaluated in this study.

In summary, our study provides a novel approach to designing inhibitors effective against ten representative high-risk CoVs, with M^pro^ inhibition below 0.6 μM for all CoVs tested, and sub-0.1 μM inhibition for six of them. This research not only offers a new perspective for developing broad-spectrum M^pro^ inhibitors against multiple CoVs, but also demonstrates that strengthening conserved interactions is an effective strategy for improving inhibitory potency. Our findings contribute to the broader effort of developing antiviral agents that can combat future viral threats, providing a foundation for future research in the design of broad-spectrum CoV inhibitors.

## Experimental section

*Chemistry: General Information.* All solvents used were purchased from Energy chemical, Macklin, Adamas-beta; they were ACS grade and used without further purification. All of the chemicals were purchased from Bidepharm, Energy chemical, Macklin, Adamas-beta, 3 A and Aladdin and used without further purification (Table S2). All reactions were monitored by thin-layer chromatography (TLC), and visualisation was achieved using ultraviolet light (254 nm) or display by an iodine reagent.^1^H NMR spectra were recorded using tetramethylsilane (TMS) as an internal standard. Chemical shifts were given in ppm (parts per million). The purity was determined by the SHIMADZU LC-2030c system with the use of XBRIDGE-C18 3.5um 2.1*50mm and analysed using H_2_O (0.05%TFA)-ACN (0.05%TFA) (10 min, flow rate: 0.8 ml/min). The purity of each compound was determined to be over 95% by reversed-phase HPLC analysis.

### General synthetic procedures

Compounds **1, 2, 5, 6, 10–14, 17, 19, 20** and **23** were obtained by an approach similar to the synthesis of compound **1**.

#### Procedure for the synthesis of intermediate 1b

A mixture of 3-(tert-butyl)-6-(ethylthio)-1,3,5-triazine-2,4(1*H*,3*H*)-dione (5750 mg, 25.0 mmol), potassium carbonate (6932 mg, 50.0 mmol), and **1a** (8464 mg, 37.6 mmol) in MeCN (30 ml) was stirred at 85 °C for 16 h. The reaction mixture was cooled to room temperature and then diluted with water and EtOAc. The aqueous layer was extracted with EtOAc. The organic layer was washed with water and brine, dried over Na_2_SO_4_, and concentrated under reduced pressure. The residue was purified by silica gel column chromatography (PE: EtOAc = 3:1) to afford **1b** (8600 mg, 89%) as a white solid. MS (ESI): 318.0 [M + H-56]^+^.

#### Procedure for the synthesis of intermediate 1c

A mixture of **1b** (6700 mg, 17.9 mmol) in THF (30 ml) and TFA (30 ml) was stirred at room temperature for 6 h. Concentration under reduced pressure afforded the residue, crude **1c** (7800 mg), as a white solid. MS (ESI): 318.0 [M + H]^+^.

#### Procedure for the synthesis of intermediate 1d

A mixture of **1c** (7800 mg, 24.6 mmol), potassium carbonate (10.2 g, 73.8 mmol), and 3-(chloromethyl)-1-methyl-1*H*-1,2,4-triazole hydrochloride (3882 mg, 29.5 mmol) in DMF (60 ml) was stirred at 60 °C for 16 h. The reaction mixture was cooled to room temperature and then diluted with water and EtOAc. The aqueous layer was extracted with EtOAc. The organic layer was washed with water and brine, dried over Na_2_SO_4_, and concentrated under reduced pressure. The residue was purified by silica gel column chromatography (DCM: MeoH = 20:1) to afford **1d** (5200 mg, 50%) as a white solid. MS (ESI): 413.0 [M + H]^+^.

#### Procedure for the synthesis of compound 1

LHMDS (0.73 ml, 0.73 mmol; 1.0 M in THF) was added dropwise to a solution of **1d** (150 mg, 0.37 mmol) and 6-chloro-2-methyl-2*H*-indazol-5-amine (80 mg, 0.43 mmol) in THF (20 ml) at 0 °C. The reaction mixture was stirred at 0 °C for 2 h. The reaction was quenched with aqueous NH_4_Cl, and the aqueous layer was extracted with EtOAc. The organic layer was washed with brine, dried over Na_2_SO_4_, and concentrated under reduced pressure. The residue was purified by silica gel column chromatography (DCM: MeoH = 20:1) to afford **1** (37.1 mg, 19%) as a white solid.

Compounds **3, 4, 7, 9, 15** and **24** were obtained by an approach similar to the synthesis of compound **3**.

#### Procedure for the synthesis of intermediate 3f2

To a solution of **3f1** (5.0 g, 28.7 mmol), 3-Hydroxytetrahydrofuran (2.53 g, 28.7 mmol), and Triphenylphosphine (11.3 g, 43.1 mmol) in THF (100 ml) was added diisopropyl azodicarboxylate (8.71 g, 43.1 mmol) at 0 °C. The reaction mixture was stirred at room temperature for 16 h. After concentration under reduced pressure, the residue was purified by silica gel column chromatography (PE: EtOAc = 3:1) to afford **3f2** (7.7 g, 92%) as yellow oil. MS (ESI) *m*/*z*: 244.0 [M + H]^+^.

#### Procedure for the synthesis of intermediate 3f

To a solution of **3f2** (6.4 g, 26.2 mmol), bis(pinacolato)diboron (9.98 g, 39.3 mmol), potassium acetate(7.71 g, 78.6 mmol)and 1,1′-bis(diphenylphosphino)ferrocene]-dichloropalladium(II)(1.92 g, 2.62 mmol) in 1,4-Dioxane (100 ml) was stirred under an nitrogen atmosphere at 80 °C for 16 h. The reaction mixture was cooled to room temperature and stirred another 1h in the presence of PE (500 ml), then filter through celite, wash the filter cake with PE, after concentration under reduced pressure, the residue was purified by C18 reversed-phase column chromatography (MeCN/0.5% Formic acid water gradient, 0 − 15% MeCN) to afford **3f** (8.0 g, 69%) as yellow solid. MS (ESI): 210.1 [M + H]^+^.

#### Procedure for the synthesis of intermediate 3d

A mixture of **3e** (2.0 g, 6.0 mmol), **3f** (3.76 g, 18.0 mmol), cupric acetate (92.18 g, 12.0 mmol), triethylamine (3.04 g, 30.0 mmol) and 4 A molecular sieve (13.2 g, 30.0 mmol) in DMF (50 ml) was stirred under an oxygen atmosphere at 60 °C for 16 h. The reaction mixture was cooled to room temperature and then diluted with water and EtOAc. The aqueous layer was extracted with EtOAc. The organic layer was washed with water and brine, dried over Na_2_SO_4_, and concentrated under reduced pressure. The residue was purified by silica gel column chromatography (DCM:MeOH = 10:1) to afford **3d** (7.2 g, 79%) as a pale-yellow solid. MS (ESI) *m*/*z*: 497.0 [M + H]^+^.

#### Procedure for the synthesis of compound 3

**3** was prepared from **3d** by an approach similar to the synthesis of compound **1**.

Compounds **8, 16, 18, 21** and **22** were obtained by an approach similar to the synthesis of compound **8**.

#### Procedure for the synthesis of intermediate 8a2

To a solution of **8a1** (1.8 g, 10.3 mmol) in THF (10 ml) was added lithium borohydride (1.12 g, 51.5 mmol) at room temperature. The reaction mixture was stirred at room temperature for 12 h, and then diluted with water. The aqueous layer was extracted with EtOAc. The organic layer was dried over Na_2_SO_4_ and concentrated under reduced pressure to afford **8a2** (1.3 g, 85%) as white solid. MS (ESI): 176.1 [M + H]^+^.

#### Procedure for the synthesis of intermediate 8a3

To a solution of **8a2** (6.7 g, 370 mmol) in acetone (100 ml) was added concentrated hydrochloric acid (15 ml) at 0 °C. The reaction mixture was stirred at 0 °C for 1 h, and then the sodium nitrite solution (3.32 g, 48 mmol) was added. The reaction mixture was stirred at 0 °C for 2 h, and then the sodium iodide solution (16.65 g, 111 mmol) was added. The reaction mixture was stirred at room temperature for 16 h, and then diluted with water. The aqueous layer was extracted with EtOAc. The organic layer was dried over Na_2_SO_4_ and concentrated under reduced pressure. The residue was purified by silica gel column chromatography (PE: EtOAc = 10:1) to afford **8a3** (4.1 g, 35%) as a pale-yellow solid. MS (ESI): 285.1 [M–H]^+^.

#### Procedure for the synthesis of intermediate 8e

**8e** was prepared from **8a** by an approach similar to the synthesis of **3**.

#### Procedure for the synthesis of compound 8

To a solution of **8e** (135 mg, 0.21 mmol) in 1,4-dioxane (10 ml) was added 3-fluorothiophenol (29 mg, 0.23 mmol), potassium carbonate (86 mg, 0.62 mmol), cupric acetate (4 mg, 0.02 mmol) and *o*-phenanthroline (3.7 mg, 0.02 mmol) at room temperature. The reaction mixture was stirred under a nitrogen atmosphere at 110 °C for 12 h. After concentration under reduced pressure, the residue was purified by silica gel column chromatography (DCM: MeOH = 20:1) and preparative HPLC to afford **8** (66.3 mg, 48%) as white solid.

#### Procedure for the synthesis of compound 25b

To a solution of **25a** (2.0 g, 14.5 mmol) and 1,1′-Carbonyldiimidazole (2.59 g, 16.0 mmol) in *N*, *N*-Dimethylacetamide (10 ml) was stirred at 0 °C, the reaction mixture was warm to room temperature for 1 h. Then 1*H*-pyrazole-1-carboximidamide (1.60 g, 14.5 mmol) and DBU (2.43 g, 16.0 mmol) was added at 0 °C, the reaction mixture was warm to room temperature for 17 h. Then 1*H*-pyrazole-1-carboximidamide (3.53 g, 21.8 mmol) and DBU (3.31 g, 21.8 mmol) was added at 0 °C, the reaction mixture was warm to room temperature for 2 h. Then 1*H*-pyrazole-1-carboximidamide (2.35 g, 14.5 mmol) and DBU (2.21 g, 14.5 mmol) was added at 0 °C, the reaction mixture was warm to room temperature for 2 h. After quenched with 2 N HCl, the reaction mixture was stirred another 1h, while the white solid was precipitated, then filtered and washed the filter cake with H_2_O to afford **25b** (2.41 g, 55%) as white solid. MS (ESI): 210.1 [M + H]^+^.

#### Procedure for the synthesis of compound 25c

To a solution of **25b** (300 mg, 0.999 mmol) and 6-chloro-2-methyl-2*H*-indazol-5-amine (181 mg, 0.999 mmol) in NMP (5 ml) was added *p*-toluenesulfonic acid (596.0 mg, 0.999 mmol) at room temperature. The reaction mixture was stirred at 80 °C for 2 h. The reaction was cooled to room temperature and then quenched with ice water, the solid was precipitated, then filtered and washed the filter cake with H_2_O to afford **25c** (299 mg, 72%) as brown solid. MS (ESI) *m*/*z*: 414.1 [M + H]^+^.

#### Procedure for the synthesis of compound 25d

A mixture of **25c** (267 mg, 0.645 mmol), **6a** (224 mg, 0.645 mmol), and DIPEA (167 mg, 1.29 mmol) in *N*, *N*-Dimethylacetamide (5 ml) was stirred at 60 °C for 3 h. The reaction mixture was diluted with water and EtOAc, and the aqueous layer was extracted with EtOAc. The organic layer was washed with water and brine, dried over Na_2_SO_4_, and concentrated under reduced pressure. The residue was purified by silica gel column chromatography (DCM: MeOH = 10:1) to afford **25d** (95 mg, 21%) as yellow oil. MS (ESI) *m*/*z*: 679.1 [M + H]^+^.

#### Procedure for the synthesis of compound 25

A mixture of **25d** (95 mg, 0.14 mmol), sodium iodide (41.9 mg, 0.28 mmol), and chlorotrimethylsilane (30.4 mg, 0.280 mmol) in Acetonitrile (5 ml) was stirred at 65 °C for 1 h. The reaction mixture was diluted with water and EtOAc, and the aqueous layer was extracted with EtOAc. The organic layer was washed with water and brine, dried over Na_2_SO_4_, and concentrated under reduced pressure. The residue was purified by silica gel column chromatography (DCM: MeOH = 20:1) and preparative HPLC to afford **25** (60 mg, 64%) as pale-yellow solid. MS (ESI) *m*/*z*: 665.1 [M + H]^+^.^1^H NMR (400 MHz, CD_3_OD) *δ* 8.15 (s, 1H), 7.72–7.67 (m, 2H), 7.44 (d, *J* = 4.2 Hz, 1H), 7.38–7.29 (m, 3H), 7.14 (d, *J* = 9.6 Hz, 1H), 6.61 (d, *J* = 4.2 Hz, 1H), 6.34 (t, *J* = 6.7 Hz, 1H), 5.32 (s, 2H), 4.86 (s, 2H), 4.17 (s, 3H).

*(E)-6-((6-chloro-2-methyl-2H-indazol-5-yl)imino)-3-(pyridin-3-yl)-1–(2,4,5-trifluorobenzyl)-1,3,5-triazinane-2,4-dione* ***(1).*** white solid. MS (ESI): 532.0 [M + H]^+^. ^1^H NMR (400 MHz, DMSO-*d*_6_, DCl in D_2_O) δ (ppm): 9.54 (s, 1H), 8.48 (s, 1H), 7.78 (s, 1H), 7.69–7.58 (m, 2H), 7.49 (s, 1H), 5.29 (s, 2H), 5.09 (s, 2H), 4.20 (s, 3H), 3.96 (s, 3H).

(E)-1–(5-chloro-2,4-difluorobenzyl)-6-((6-chloro-2-methyl-2H-indazol-5-yl)imino)-3-((1-methyl-1H-1,2,4-triazol-3-yl)methyl)-1,3,5-triazinane-2,4-dione **(2)**. White solid. MS (ESI) *m*/*z*:547.7 [M + H]^+^. ^1^H NMR (400 MHz, MeOD) δ (ppm): 8.36 (s, 1H), 8.19 (s, 1H), 7.75–7.69 (m, 2H), 7.41 (s, 1H), 7.20 (t, *J* = 9.5 Hz, 1H), 5.36 (s, 2H), 5.12 (s, 2H), 4.21 (s, 3H), 3.91 (s, 3H).

(S,E)-1–(5-Chloro-2,4-difluorobenzyl)-6-((6-chloro-2-methyl-2H-indazol-5-yl)imino)-3–(5-((tetrahydrofuran-3-yl)oxy)pyridin-3-yl)-1,3,5-triazinane-2,4-dione **(3)**. White solid. MS (ESI): 615.9 [M + H]^+^. ^1^H NMR (400 MHz, CD_3_OD) δ (ppm): 8.33 (s, 1H), 8.25 (s, 1H), 8.18 (s, 1H), 7.81 (t, *J* = 7.8 Hz, 1H), 7.71 (s, 1H), 7.69–7.67 (m, 1H), 7.45 (br, 1H), 7.18 (t, *J* = 9.5 Hz, 1H), 5.35 (s, 2H), 5.11 (d, *J* = 1.4 Hz, 1H), 4.18 (s, 3H), 3.95 (d, *J* = 3.1 Hz, 3H), 3.87 − 3.83 (m, 1H), 2.32–2.23 (m, 1H), 2.16–2.09 (m, 1H).

(E)-1–(5-chloro-2,4-difluorobenzyl)-6-((6-chloro-2-methyl-2H-indazol-5-yl)imino)-3–(5-(2-methoxyethoxy)pyridin-3-yl)-1,3,5-triazinane-2,4-dione **(4)**. White solid. MS (ESI) *m*/*z*:604.0 [M + H]^+^. ^1^HNMR (400 MHz, CD_3_OD) δ (ppm): 8.26 (d, *J* = 2.6 Hz, 1H), 8.18–8.10 (m, 2H), 7.94–7.63 (m, 3H), 7.52–7.50 (m, 1H), 7.16 (s, 1H), 5.33 (s, 2H), 4.17–4.15 (m, 2H), 4.14 (s, 3H), 3.72–3.70 (m, 2H), 3.37 (s, 3H).

(E)-4–(2-((6-((6-chloro-2-methyl-2H-indazol-5-yl)imino)-3-((1-methyl-1H-1,2,4-triazol-3-yl)methyl)-2,4-dioxo-1,3,5-triazinan-1-yl)methyl)-4,5-difluorophenoxy)-benzonitrile **(5)**. Yellow solid. MS (ESI) *m*/*z*: 631.0 [M + H]^+^. ^1^H NMR (400 MHz, CD_3_OD) δ (ppm): 8.33 (s, 1H), 8.11 (s, 1H), 7.68 (s, 1H), 7.60–7.49 (m, 3H), 7.09–7.04 (m, 4H), 5.25 (s, 2H), 5.04–5.02 (m, 2H), 4.19 (s, 3H), 3.89 (s, 3H).

(E)-5–(4-chloro-2-((6-((6-chloro-2-methyl-2H-indazol-5-yl)imino)-3-((1-methyl-1H-1,2,4-triazol-3-yl)methyl)-2,4-dioxo-1,3,5-triazinan-1-yl)methyl)-5-fluorophenoxy)-thiophene-2-carbonitrile **(6)**. Yellow solid. MS (ESI) *m*/*z*: 653.1 [M + H]^+^. ^1^H NMR (400 MHz, DMSO-d_6_) δ (ppm): 11.04 (br, 1H), 8.43–8.21 (m, 2H), 7.85–7.61 (m, 3H), 7.49 (d, *J* = 9.8 Hz, 1H), 7.40–7.10 (m, 1H), 6.83 (d, *J* = 3.9 Hz, 1H), 5.24 (s, 2H), 4.91 (s, 2H), 4.15 (s, 3H), 3.81 (s, 3H).

(E)-4–(4-chloro-2-((6-((6-chloro-2-methyl-2H-indazol-5-yl)imino)-3–(5-fluoro-pyridin-3-yl)-2,4-dioxo-1,3,5-triazinan-1-yl)methyl)-5-fluorophenoxy)-benzonitrile **(7)**. Yellow solid. MS (ESI) *m*/*z*: 647.1 [M + H]^+^. ^1^H NMR (400 MHz, DMSO-d_6_) δ (ppm): 8.66 (s, 1H), 8.41 (d, *J* = 41.1 Hz, 2H), 8.09–7.71 (m, 5H), 7.35–7.11 (m, 3H), 5.16 (s, 2H), 4.16 (s, 3H).

(E)-6-((6-Chloro-2-methyl-2H-indazol-5-yl)imino)-1–(5-chloro-4-fluoro-2-((3-fluorophenyl)thio)benzyl)-3–(5-methylpyridin-3-yl)-1,3,5-triazinane-2,4-dione **(8).** White solid. MS (ESI): 652.2 [M + H]^+^. ^1^H NMR (400 MHz, CD_3_OD) δ (ppm): 8.76–8.41 (m, 2H), 8.16 (s, 1H), 8.03 (s, 1H), 7.80 (d, *J* = 7.3 Hz, 1H), 7.70 (s, 1H), 7.41 (s, 1H), 7.30–7.19 (m, 2H), 7.06 (d, *J* = 7.9 Hz, 1H), 7.01–6.96 (m, 1H), 6.91 (t, *J* = 8.5 Hz, 1H), 5.39 (s, 2H), 4.17 (s,3H), 2.46 (s, 3H).

(E)-6-((6-chloro-2-methyl-2H-indazol-5-yl)imino)-1–(5-chloro-4-fluoro-2–(4-fluorophenoxy)benzyl)-3-((1-methyl-1H-1,2,4-triazol-3-yl)methyl)-1,3,5-triazinane-2,4-dione **(9)**. White solid. MS (ESI) *m*/*z*:640.1 [M + H]^+^. ^1^H NMR (400 MHz, CD_3_OD) δ (ppm): 8.35 (s, 1H), 8.19 (s, 1H), 7.73 (s, 1H), 7.65 (d, *J* = 8.1 Hz, 1H), 7.43 (s, 1H), 7.12 (d, *J* = 6.0 Hz, 4H), 6.69 (d, *J* = 10.2 Hz, 1H), 5.39 (s, 2H), 5.10 (s, 2H), 4.21 (s, 3H), 3.91 (s, 3H).

(E)-1–(5-chloro-2–(3-chlorophenoxy)-4-fluorobenzyl)-6-((6-chloro-2-methyl-2H-indazol-5-yl)imino)-3-((1-methyl-1H-1,2,4-triazol-3-yl)methyl)-1,3,5-triazinane-2,4-dione **(10).** White solid. MS (ESI) *m*/*z*: 656.1 [M + H]^+^. ^1^H NMR (400 MHz, DMSO-d6) δ 8.31 (dd, *J* = 57.0, 37.1 Hz, 2H), 7.83–7.36 (m, 3H), 7.33–7.20 (m, 1H), 7.16–6.98 (m, 4H), 5.17 (d, *J* = 47.7 Hz,2H), 4.84 (d, *J* = 12.0 Hz, 2H), 4.12 (d, *J* = 22.2 Hz, 3H), 3.76 (d, *J* = 13.2 Hz, 3H).

(E)-1-(5-chloro-2–(3,4-difluorophenoxy)-4-fluorobenzyl)-6-((6-chloro-2-methyl-2H-indazol-5-yl)imino)-3-((1-methyl-1H-1,2,4-triazol-3-yl)methyl)-1,3,5-triazinane-2,4-dione **(11)**. White solid. MS (ESI) *m*/*z*:658.1 [M + H]^+^. ^1^H NMR (400 MHz, CD_3_OD) δ (ppm): 8.34 (s, 1H), 8.13 (s, 1H), 7.78–7.62 (m, 2H), 7.09 (d, *J* = 45.0 Hz, 3H), 6.83 (d, *J* = 9.9 Hz, 2H), 5.35 (s, 2H), 5.10 (s, 2H), 4.21 (s, 3H), 3.91 (s, 3H).

(E)-6-((6-chloro-2-methyl-2H-indazol-5-yl)imino)-1–(5-chloro-4-fluoro-2-((5-fluoropyridin-2-yl)oxy)benzyl)-3-((1-methyl-1H-1,2,4-triazol-3-yl)methyl)-1,3,5-triazinane-2,4-dione **(12)**. White solid. MS (ESI) *m*/*z*:641.0 [M + H]^+^. ^1^H NMR (400 MHz, Chloroform-d) δ (ppm): 8.04 (s, 1H), 7.76 (s, 1H), 7.60 (d, *J* = 7.9 Hz, 1H), 7.42 (s, 1H), 5.31 (s, 1H), 5.19 (s, 1H), 4.21 (s, 2H), 3.89 (s, 1H).

(E)-1–(5-chloro-2-((4,4-difluorocyclohexyl)oxy)-4-fluorobenzyl)-6-((6-chloro-2-methyl-2H-indazol-5-yl)imino)-3-((1-methyl-1H-1,2,4-triazol-3-yl)methyl)-1,3,5-triazinane-2,4-dione **(13)**. White solid. MS (ESI) *m*/*z*: 664.1 [M + H]^+^. ^1^H NMR (400 MHz, CD_3_OD) δ (ppm): 8.32–8.08 (m, 2H), 7.80–7.63 (m, 1H), 7.45 (d, *J* = 8.0 Hz, 1H), 7.13–6.98 (m, 2H), 5.26 (s, 2H), 5.11 (s, 2H), 4.66 (d, *J* = 17.2 Hz, 1H), 4.18 (d, *J* = 14.0 Hz, 3H), 3.88 (d, *J* = 9.6 Hz, 3H), 2.23–2.12 (m, 2H), 2.10–1.98 (m, 6H).

(E)-6-((6-chloro-2-methyl-2H-indazol-5-yl)imino)-1–(5-chloro-4-fluoro-2-((5-(trifluoromethyl)thiazol-2-yl)oxy)benzyl)-3-((1-methyl-1H-1,2,4-triazol-3-yl)methyl)-1,3,5-triazinane-2,4-dione **(14)**. Pale-yellow solid. MS (ESI) *m*/*z*: 697.2 [M + H]^+^. ^1^H-NMR (400 MHz, DMSO-d6) δ 8.42 (d, *J* = 27.4 Hz, 1H), 8.21 (s, 1H), 7.96–7.90 (m, 1H), 7.82 (d, *J* = 5.4 Hz, 1H), 7.66 (d, *J* = 12.8 Hz, 1H), 7.10 (t, *J* = 51.2 Hz, 3H), 5.31 (s, 3H), 4.90 (d, *J* = 6.8 Hz, 2H), 4.15 (d, *J* = 23.3 Hz, 3H), 3.83–3.76 (m, 3H).

(E)-4-((4-chloro-2-((6-((6-chloro-2-methyl-2H-indazol-5-yl)imino)-3–(5-methylpyridin-3-yl)-2,4-dioxo-1,3,5-triazinan-1-yl)methyl)-5-fluoro-phenyl)thio)benzonitrile **(15)**. White solid. MS (ESI) *m*/*z*:659.2 [M + H]^+^. ^1^H NMR (400 MHz, CD_3_OD) δ (ppm): 8.50 (s, 1H), 8.13 (s, 1H), 7.94–7.84 (m, 3H), 7.69 (s, 1H), 7.49 (d, *J* = 8.7 Hz, 1H), 7.36 (d, *J* = 10.7 Hz, 2H), 7.21 (d, *J* = 8.2 Hz, 3H), 5.37 (s, 2H), 4.17 (s, 3H), 2.44 (s, 3H).

(E)-6-((6-chloro-2-methyl-2H-indazol-5-yl)imino)-1–(5-chloro-4-fluoro-2-((4-fluorophenyl)thio)benzyl)-3–(5-methylpyridin-3-yl)-1,3,5-triazinane-2,4-dione **(16)**. White solid. MS (ESI) *m*/*z*:652.2 [M + H]^+^. ^1^H NMR (400 MHz, CD_3_OD) δ (ppm): 8.61 (s, 1H), 8.23–8.10 (m, 2H), 7.82 (d, *J* = 8.4 Hz, 1H), 7.71 (s, 1H), 7.65 (d, *J* = 7.3 Hz, 1H), 7.51 (s, 1H), 5.22 (s, 2H), 4.18 (s,3H), 2.49 (d, *J* = 2.6 Hz, 3H).

(E)-6-((6-chloro-2-methyl-2H-indazol-5-yl)imino)-1–(3-chloro-4-fluoro-5-((2-methoxyethyl)thio)benzyl)-3-((1-methyl-1H-1,2,4-triazol-3-yl)methyl)-1,3,5-triazinane-2,4-dione **(17)**. White solid. MS (ESI) *m*/*z*: 620.2 [M + H]^+^. ^1^H NMR (400 MHz, DMSO-d6) δ (ppm): 11.05 (s, 1H), 9.55 (s, 1H), 8.34 (m, 2H), 7.73 (s, 1H), 7.31 (d, *J* = 144.5 Hz, 3H), 5.19 (t, *J* = 31.5 Hz, 2H), 4.92 (s, 2H), 4.15 (s, 3H), 3.80 (s, 3H), 3.51 (s, 2H), 3.19 (s, 5H).

6-((6-chloro-2-methyl-2H-indazol-5-yl)amino)-1–(3-chloro-4-fluoro-5-((3-fluorophenyl)thio)benzyl)-3–(5-methylpyridin-3-yl)-1,3,5-triazine-2,4(1H,3H)-dione **(18).** White solid. MS (ESI) *m*/*z*: 652.1 654.1 [M + H]^+^. ^1^H NMR (400 MHz, DMSO-d_6_) δ (ppm): 11.17 (s, 0.55H), 9.60 (s, 0.45H), 8.55–8.20 (m, 3H), 7.93–7.28 (m, 6H), 7.18–7.08 (m, 3H), 5.31–5.11 (m, 2H), 4.16 (s, 3H), 2.35 (s, 3H).

(E)-1–(2-(benzyloxy)-4,5-difluorobenzyl)-6-((6-chloro-2-methyl-2H-indazol-5-yl)imino)-3-((1-methyl-1H-1,2,4-triazol-3-yl)methyl)-1,3,5-triazinane-2,4-dione **(19).** White solid. MS (ESI) *m*/*z*: 620.0 [M + H]^+^.^1^H NMR (400 MHz, DMSO-d_6_) δ (ppm): 8.33 (s, 1H), 8.24 (s, 1H), 7.66 (s, 1H), 7.54–7.16 (m, 8H), 5.26–5.11 (m, 4H), 4.88 (s, 2H), 4.13 (s, 3H), 3.78 (s, 3H).

(E)-6-((6-chloro-2-methyl-2H-indazol-5-yl)imino)-1–(4,5-difluoro-2-(phenoxymethyl)benzyl)-3-((1-methyl-1H-1,2,4-triazol-3-yl)methyl)-1,3,5-triazinane-2,4-dione **(20)**. White solid. MS (ESI) *m/z*: 620.0 [M + H]^+^.^1^H NMR (400 MHz, DMSO-d_6_) δ (ppm): 8.39–8.27 (m, 2H), 7.75–7.73 (m, 1H), 7.65 (s, 1H), 7.48 (s, 1H), 7.32–6.93 (m, 6H), 5.37–5.25 (m, 4H), 4.95 (s, 2H),4.18–4.13(m, 3H), 3.81 (s, 3H).

(E)-6-((6-chloro-2-methyl-2H-indazol-5-yl)imino)-1–(4,5-difluoro-2-((E)-styryl)benzyl)-3-((1-methyl-1H-1,2,4-triazol-3-yl)methyl)-1,3,5-triazinane-2,4-dione **(21).** White solid. MS (ESI) *m/z*: 616.2 [M + Na]^+^. ^1^H NMR (400 MHz, CD_3_OD) δ 8.24 (s, 1H), 8.10 (s, 1H), 7.66 (s, 1H), 7.58–7.35 (m, 5H), 7.33–7.13 (m, 4H), 7.01 (d, *J* = 14.4 Hz, 1H), 5.40 (s, 2H), 5.04 (s, 2H), 4.14 (s, 3H), 3.80 (s, 3H).

(E)-6-((6-chloro-2-methyl-2H-indazol-5-yl)imino)-1–(5-chloro-4-fluoro-2-((E)-2-(thiazol-5-yl)vinyl)benzyl)-3-((1-methyl-1H-1,2,4-triazol-3-yl)methyl)-1,3,5-triazinane-2,4-dione **(22).** White solid. MS (ESI) *m/z*: 639.1 [M + H]^+^. ^1^H NMR (400 MHz, CD_3_OD) δ 8.87 (s, 1H), 8.34 (s, 1H), 8.13 (s, 1H), 7.91 (s, 1H), 7.67 (s, 1H), 7.62 (d, *J* = 7.4 Hz, 1H), 7.55 (d, *J* = 10.5 Hz, 1H), 7.42 (s, 1H), 7.35 (d, *J* = 15.6 Hz, 2H), 5.38 (s, 2H), 5.09 (s, 2H), 4.16 (s, 3H), 3.84 (s, 3H).

(E)-3-((6-((6-Chloro-2-methyl-2H-indazol-5-yl)imino)-3–(5-(cyclopropylthio)-pyridin-3-yl)-2,4-dioxo-1,3,5-triazinan-1-yl)methyl)-4-methylbenzonitrile **(23)**. White solid. MS (ESI): 571.1 [M + H]^+^. ^1^H NMR (400 MHz, DMSO-d_6_) δ (ppm): 8.55 (s, 1H), 8.38 (s, 2H), 7.97 (s, 1H), 7.89 (s, 2H), 7.68 (s, 2H), 7.42 (s, 1H), 5.20 (s, 2H), 4.16 (s, 3H), 2.42 (s, 3H), 2.36 (m, 1H), 1.13 (d, *J* = 5.6 Hz, 2H), 0.64 (d, *J* = 2.4 Hz, 2H).

(E)-3-((6-((6-Chloro-2-methyl-2H-indazol-5-yl)imino)-3–(5-methylpyridin-3-yl)-2,4-dioxo-1,3,5-triazinan-1-yl)methyl)-4-((5-chlorothiophen-2-yl)thio)benzonitrile **(24)**. White solid. MS (ESI): 647.1 649.1 [M + H]^+^. ^1^H NMR (400 MHz, DMSO-d_6_) δ (ppm): 11.26 (s, 0.6H), 9.71 (s, 0.4H), 8.36 (d, *J* = 75.1 Hz, 3H), 8.23–8.01 (m, 1H), 7.77 (t, *J* = 34.3 Hz, 3H), 7.49 (d, *J* = 3.3 Hz, 1H), 7.30 (s, 1H), 7.12 (d, *J* = 16.3 Hz, 2H), 5.26 (s, 2H), 4.15 (s, 3H), 2.36 (s, 3H).

(E)-5–(4-chloro-2-((6-((6-chloro-2-methyl-2H-indazol-5-yl)imino)-2,4-dioxo-3-((2-oxo-1,2-dihydropyridin-3-yl)methyl)-1,3,5-triazinan-1-yl)methyl)-5-fluoro-phenoxy)thiophene-2-carbonitrile **(25).** Pale-yellow solid. MS (ESI) *m/z*: 665.1 [M + H]^+^. ^1^H NMR (400 MHz, CD_3_OD) δ (ppm): 8.15 (s, 1H), 7.72–7.67 (m, 2H), 7.44 (d, *J* = 4.2 Hz, 1H), 7.38–7.29 (m, 3H), 7.14 (d, *J* = 9.6 Hz, 1H), 6.61 (d, *J* = 4.2 Hz, 1H), 6.34 (t, *J =* 6.7 Hz, 1H), 5.32 (s, 2H), 4.86 (s, 2H), 4.17 (s, 3H).

### Cloning, expression and purification of CoVs M^pro^

The original expression vector of CoVs M^pro^, with a cleavable C-terminal His-tag, enabling M^pro^ constructs with native termini to be generated, was produced by a biological company (IGE Biotechnology). The recombinant plasmid was introduced into *E.coli* BL21 (DE3), which were grown in Luria–Bertani (LB) broth containing 100 µg/mL ampicillin at 37 °C. Isopropyl β-d-1-thiogalactopyranoside (IPTG) was introduced at a concentration of 500 μM to initiate protein expression at 16 °C when the optical density of the culture reached 0.6–0.8 at 600 nm. Following an overnight induction period, the cells were collected by centrifugation at 5,000 rpm. The cells were then disrupted using high-pressure homogenisation, and the mixture was centrifuged at 18,000 rpm for 40 min to separate the supernatant. This supernatant was applied to a Ni–NTA affinity column from Cytiva, using a binding buffer composed of 50 mM Tris–HCl at pH 8.0, 500 mM NaCl, and 10% glycerol. The protein was subsequently eluted using an elution buffer that included 50 mM Tris–HCl at pH 8.0, 500 mM NaCl, 10% glycerol, and 500 mM imidazole.

The protein, once eluted, was subjected to concentration using an ultrafiltration system at 3,500 rpm and a temperature of 4 °C, with a membrane that has a molecular weight limit of 30 kDa. Post-centrifugation, the portions rich in M^pro^ were isolated and incubated with prescission protease (PPase) at a cool temperature of 4 °C for an extended period to cleave off the C-terminal His-tag. The protein solution that had been treated with PPase was then passed through a HisTrap FF column from GenScript, which was rinsed with a buffer devoid of imidazole. Subsequently, the purified protein was introduced to a Superdex 200 prep-grade column from Cytiva, pre-conditioned with a solution of 20 mM HEPES at a pH of 7.4 and 150 mM NaCl, and the segment displaying the most significant peak was gathered. The refined CoVs M^pro^ was preserved in a solution of 50 mM Tris at a pH of 7.4, accompanied by 1 mM EDTA (Figure S2).

### Crystallisation, data collection and structure determination

The CoVs M^pro^ was mixed with the compounds at a ratio of one molecule of protein to six molecules of compound and allowed to interact for 60 min at a cold temperature of 4 °C. Following this incubation period, the combined solutions were subjected to crystallisation using the hanging drop vapour diffusion method at a temperature of 16 °C.

X-ray diffraction data were acquired at beamlines BL18U1 and BL19U1 within the Shanghai Synchrotron Radiation Facility (SSRF), with measurements taken at a temperature of 100 K and a radiation wavelength of 0.97983 Å. The process of data integration and scaling was carried out using the XDSgui software version 24. The determination of all structures was achieved through molecular replacement, utilising the Phaser module within the PHENIX software suite version 25. For compound 1, the structure was resolved by employing the M^pro^ structure (RCSB Protein Data Bank (PDB) entry 7VU6) as a reference template. Other structures were resolved using the compound 1-Mpro complex as the reference template. The initial model from molecular replacement was refined through several rounds of manual adjustment using Coot version 26, and the final refinement was accomplished with PHENIX. A summary of the phasing and refinement statistics can be found in Table 7.

### Enzymatic activity and inhibition assays

The measurement of CoVs M^pro^ activities was conducted through a continuous kinetic assay utilising the substrate MCA-AVLQSGFR-Lys(Dnp)-Lys-NH2 (Beyotime). The assay employed 320 nm for substrate excitation and 405 nm for emission. CoVs M^pro^ were prepared in a kinetic assay buffer composed of 50 mM Tris–HCl at pH 7.5, 1 mM EDTA, at specific concentrations: 0.2 μM for Ericov, HKU2, HKU8, HKU10, PEDV, FIPV, Rs4081, Rs4231, RATG13, BANAL-236 and SARS-CoV**-**2; and 0.5 μM for SAX2011, LRNV, and MjHKU4r-CoV-1. The assay was started by adding concentrations of substrate 20 μM to the diluted M^pro^. The multimode plate reader was used to track fluorescence intensity, and initial velocities were calculated by linearising the curves’ linear segments with a specific formula.

## Supplementary Material

Revised_Supplementary Information20250428.docx

## Data Availability

The authors confirm that the data supporting the findings of this study are available within the article and its supplemental materials.

## Abbreviations

CoVs: Coronavirus
HCoVs: Human infection Coronavirus
SARS-CoV-2: Severe Acute Respiratory Syndrome Coronavirus 2
MERS-CoV: Middle East Respiratory Syndrome Coronavirus
M^pro^: The main protease
NSP5: Non-structural protein 5
SARS-CoV-1: Severe Acute Respiratory Syndrome Coronavirus 1
LiBH_4_: Lithium borohydride
THF: Tetrahydrofuran
CBr_4_: Tetrabromomethane
PPh_3_: Triphenylphosphine
DCM: Dichloromethane
CuCl: Copper(I) chloride
Cs_2_CO_3_: Caesium carbonate
TMHD: 2,2,6,6-Tetramethyl-3,5-heptanedione
NMP: *N*-Methylpyrrolidone
NBS: *N*-Bromosuccinimide
AIBN: 2,2′-Azobis(2-methylpropionitrile)
ACN: Acetonitrile
DIAD: Diisopropyl azodicarboxylate
DMF: *N,N*-Dimethylformamide
B_2_Pin_2_: Bis(pinacolato)diboron
KOAc: Potassium Acetate
Pd(dppf)Cl_2_: 1,1′-Bis(diphenylphosphino)ferrocene]dichloropalladium(II)
K_2_CO_3_: Potassium carbonate
TFA: Trifluoroacetic acid;LHMDS, Lithium bis(trimethylsilyl)amide
Cu(OAc)_2_: Copper(II) acetate
DMAP: 4-Dimethylaminopyridine
NaNO_2_: Sodium nitrite
Pd(OAc)_2_: Palladium (II) Acetate
NEt_3_: Triethylamine
CDI: 1,1′-Carbonyldiimidazole
DBU: 1,8-Diazabicyclo[5.4.0]undec-7-ene
PTSA: p-Toluenesulfonic acid
DIPEA: *N,N*-Diisopropylethylamine
NaI: Sodium iodide
TMSCl: chlorotrimethylsilane
