# Supplementary material for: Identification of broad-spectrum Mpro inhibitors: a focus on high-risk coronaviruses and conserved interactions
Source: J Enzyme Inhib Med Chem. 2025 May 21;40(1):2503961. doi: 10.1080/14756366.2025.2503961 (PMC12096674; doi:10.1080/14756366.2025.2503961)
Supplement: Revised_Supplementary Information20250428.docx [file IENZ_A_2503961_SM7811.docx]

**SUPPORTING INFORMATION**

**Identification of Broad-Spectrum M^pro^ Inhibitors: A Focus on High-Risk Coronaviruses and Conserved Interactions**

**Table of contents**

[**1.CoVs detailed information 2**](#_Toc196744726)

[**2. Protein amino acid conservation comparisons 10**](#_Toc196744727)

[**3. Protein purification 11**](#_Toc196744728)

[**4. Synthesis procedures 14**](#_Toc196744729)

[**5. Spectral data: NMR and MS/HPLC traces for compounds 2-25 38**](#_Toc196744730)

[**6. The source and catalogue number of the chemicals and assays 75**](#_Toc196744731)

**EXPERIMENTAL SECTION**

## CoVs detailed information

**Table S1.** 24 CoVs detailed information

|  | **Viruses** | **Host** | **Abbrevations** | **Accession ID** | **Protein sequences** |
| --- | --- | --- | --- | --- | --- |
| 1 | Human coronavirus OC43 | Homo sapiens | OC43 | NC_006213.1 | AVLSGIVKMVNPTSKVEPCVVSVTYGNMTLNGLWLDDKVYCPRHVICSASDMTNPDYTNLLCRVTSSDFTVLFDRLSLTVMSYQMRGCMLVLTVTLQNSRTPKYTFGVVKPGETFTVLAAYNGKPQGAFHVTMRSSYTIKGSFLCGSCGSVGYVIMGDCVKFVYMHQLELSTGCHTGTDFNGDFYGPYKDAQVVQLLIQDYIQSVNFVAWLYAAILNNCNWFVQSDKCSVEDFNVWALSNGFSQVKSDLVIDALASMTGVSLETLLAAIKRLKNGFQGRQIMGSCSFEDELTPSDVYQQLAGIKFQGP |
| 2 | Porcine hemagglutinating encephalomyelitis virus | Pig | PHEV | WCJ83077.1 | vkmvnptskvepcivsvtygnmtlnglwlddkvycprhvicsasdmtnpdytnllcrvtssdfivlfdrlsltvmsyqmqgcmlvltvtlqnsrtpkytfgvvkpgetftvlaayngkpqgafhvtmrssytikgsflcgscgsvgyvmmgdcvkfvymhqlelstgchtgtdfngdfygpykdaqvvqlpvqdyiqsvnfvawlyaailnncnwfvqsdkcsvedfnvwalsngfsqfksdlvidalasmtgvsletllaaikrlkngfqgrqimgscsfedeltpsdvyqql |
| 3 | Murine hepatitis virus strain A59 | Mouse | MHV A59 | ATN37894.1 | SGIVKMVSPTSKVEPCIVSVTYGNMTLNGLWLDDKVYCPRHVICSSADMTDPDYPNLLCRVTSSDFCVMSGRMSLTVMSYQMQGCQLVLTVTLQNPNTPKYSFGVVKPGETFTVLAAYNGRPQGAFHVTLRSSHTIKGSFLCGSCGSVGYVLTGDSVRFVYMHQLELSTGCHTGTDFSGNFYGPYRDAQVVQLPVQDYTQTVNVVAWLYAAIFNRCNWFVQSDSCSLEEFNVWAMTNGFSSIKADLVLDALASMTGVTVEQVLAAIKRLHSGFQGKQILGSCVLEDELTPSDVYQQLAGVKLQ |
| 4 | Rousettus bat coronavirus HKU9 | Bat | BtCoV-HKU9 | NC_009021 | AGLTRMAHPSGLVEPCLVKVNYGSMTLNGIWLDNFVICPRHVMCSRDELANPDYPRLSMRAANYDFHVSQNGHNIRVIGHTMEGSLLKLTVDVNNPKTPAYSFIRVSTGQAMSLLACYDGLPTGVYTCTLRSNGTMRASFLCGSCGSPGFVMNGKEVQFCYLHQLELPNGTHTGTDFSGVFYGPFEDKQVPQLAAPDCTITVNVLAWLYAAVLSGENWFLTKSSISPAEFNNCAVKYMCQSVTSESLQVLQPLAAKTGISVERMLSALKVLLSAGFCGRTIMGSCSLEDEHTPYDIGRQMLGVKLQ |
| 5 | Bat Hp-betacoronavirus/Zhejiang2013 | Bat | BtCoV-ZJ2013 | NC_025217 | SGIRKMSCPTGKVERCMVRVTCGTMTLNGLWLDNTVYCPRHVMCTPEELLAPDYDSILLRKATHSFTVQYGTAYLKVVSYKMTGSVLQLGVDQINPETPKYKFVRAKPGATFSVLACYNGMPAGVYQVAMRPNHTIKGSFLNGSCGSVGYTLGYDRVEFCYMHHMELPTGVHTGTDLEGTFYGDFVDRQTSQSAGSDNTLTLNVLAWLYAAVINGERWFIVPQTCALTDFNTAVLKYGYQSLTEDGVAALDPLVAQTGISVQTMCASLKDLLVHGMRGRCILSSPTLEDEFTPFDIVRQCSGVTLQ |
| 6 | Severe acute respiratory syndrome coronavirus 2 | Homo sapiens | SARS-COV-2 | QTN76269.1 | SGFRKMAFPSGKVEGCMVQVTCGTTTLNGLWLDDVVYCPRHVICTSEDMLNPNYEDLLIRKSNHNFLVQAGNVQLRVIGHSMQNCVLKLKVDTANPKTPKYKFVRIQPGQTFSVLACYNGSPSGVYQCAMRPNFTIKGSFLNGSCGSVGFNIDYDCVSFCYMHHMELPTGVHAGTDLEGNFYGPFVDRQTAQAAGTDTTITVNVLAWLYAAVINGDRWFLNRFTTTLNDFNLVAMKYNYEPLTQDHVDILGPLSAQTGIAVLDMCASLKELLQNGMNGRTILGSALLEDEFTPFDVVRQCSGVTFQ |
| 7 | Severe acute respiratory syndrome-related coronavirus | Rhinolophus sinicus | SARS-COV | WEG19431.1 | SGFRKMAFPSGKVEGCMVQVTCGTTTLNGLWLDDTVYCPRHVICTAEDMLNPNYEDLLIRKSNHSFLVQAGNVQLRVIGHSMQNCLLRLKVDTSNPKTPKYKFVRIQPGQTFSVLACYNGSPSGVYQCAMRPNHTIKGSFLNGSCGSVGFNIDYDCVSFCYMHHMELPTGVHAGTDLEGKFYGPFVDRQTAQAAGTDTTITLNVLAWLYAAVINGDRWFLNRFTTTLNDFNLVAMKYNYEPLTQDHVDILGPLSAQTGIAVLDMCAALKELLQNGMNGRTILGSTILEDEFTPFDVVRQCSGVTFQ |
| 8 | ErinaceusCoV/2012-174/GER/2012 | Erinaceus europaeus | EriCoV | YP_009513009.1 | SGLVKMAHPSGAVEQCIVQVTCGSMTLNGLWLDNIVYCPRHVMCPQDQLVDPNYDALLNSMTNHSFTIQRHGRSTANLRCTGHAMHGTLLKLTVDSANPETPAYTFTTIKQGSSFSVLACYNGRPSGTYTVVMRPNSTIKGSFLCGSCGSVGYVKEGNVINFCYMHQMELSNGTHTGSSFDGNMYGNFQDRQIYQAQLSDKHCTINVVAWLYAAVLNGCNWFVKPNKTGVAAFNEWALSNQFTEFVSTQALELLAVKTGVQIEQLLYSIQQLNNGFQGNVILGSAMLEDEYTPEDVNMQMMGVVMQ |
| 9 | Pipistrellus bat coronavirus HKU5 | Bat | BtCoV-HKU5 | NC_009020 | SGLVKMAAPSGVVENCMVQVTCGSMTLNGLWLDNYVWCPRHVMCPADQLSDPNYDALLVSKTNLSFIVQKNVGAPANLRVVGHTMVGTLLKLTVESANPQTPAYTFTTVKPGASFSVLACYNGRPTGVFMVNMRQNSTIKGSFLCGSCGSVGYTQEGNVINFCYMHQMELSNGTHTGCAFDGVMYGAFEDRQVHQVQLSDKYCTINIVAWLYAAILNGCNWFVKPNKTGIATFNEWAMSNQFTEFIGTQSVDMLAHKTGVSVEQLLYAIQTLHKGFQGKTILGNSMLEDEFTPDDVNMQVMGVVMQ |
| 10 | Middle East respiratory syndrome-related coronavirus | Camelus dromedarius | MERS-CoV | WXL51590.1 | AVLSGLVKMSHPSGDVEACMVQVTCGSMTLNGLWLDNTVWCPRHVMCPADQLSDPNYDALLISMTNHSFSVQKYIGAPANLRVVGHAMQGTLLKLTVDVANPSTPAYTFTTVKPGASFSVLACYNGRPTGTFTVVMRPNYTIKGSFLCGSCGSVGYTKEGSVINFCYMHQMELANGTHTGSAFDGTMYGAFMDKQVHQVQLTDKYCSVNVVAWLYAAILNGCAWFVKPNRTSVVSFNEWALANQFTEFVGTQSVDMLAVKTGVAIEQLLYAIQQLYTGFQGKQILGSTMLEDEFTPEDVNMQIMGVVFQGP |
| 11 | Tylonycteris bat coronavirus HKU4 | Bat | BtCoV HKU4 | AWH65876.1 | SGLVKMSAPSGAVENCIVQVTCGSMTLNGLWLDNTVWCPRHIMCPADQLTDPNYDALLISKTNHSFIVQKHIGAQANLRVVAHSMVGVLLKLTVDVANPSTPAYTFSTVKPGASFSVLACYNGKPTGVFTVNLRHNSTIKGSFLCGSCGSVGYTENGGVINFVYMHQMELSNGTHTGSSFDGVMYGAFEDKQTHQLQLTDKYCTINVVAWLYAAVLNGCKWFVKPTRVGIVTYNEWALSNQFTEFVGTQSIDMLAHRTGVSVEQMLAAIQSLHAGFQGKTILGQSTLEDEFTPDDVNMQVMGVVMQ |
| 12 | Rhinolophus bat coronavirus HKU2 | Bat | Bat_HKU2 | YP_001552235.1 | AGLKKMAQPSGLVEPCVVRVSYGNTVLNGVWLDDKVYCPRHVLASDTTVTIDYDAVYHSMRLHNFSISKGNVFLGVVGAVMQGANLVITVSQANVNTPSYSFRTLKAGECFNILACYDGTPAGVYGVNLRSTHTIKGSFVNGACGSPGFVMNGYKVEFVYMHQIELGNASHVGSDMFGNIYGGFEDQPSIQLEGVATLITENVVAFLYAALINGERWWCSNERCTIDSFNEWALGNGFTNLVSGDGFSMLAAKTGVDVCQLLSAIQRLATGLGGKTILGYASVTDEYTLSEVVRQMYGVNIQ |
| 13 | Feline coronavirus | Cat | FIPV | AAY32595.1 | SGLRKMAQPSGVVEPCIVRVAYGNNVLNGLWLGDEVICPRHVIASDTSRVINYENELSSVRLHNFSIAKNNAFLGVVSAKYKGVNLVLKVNQVNPNTPEHKFKSVRPGESFNILACYEGCPGSVYGVNMRSQGTIKGSFIAGTCGSVGYVLENGTLYFVYMHHLELGNGSHVGSNLEGEMYGGYEDQPSMQLEGTNVMSSDNVVAFLYAALINGERWFVTNTSMTLESYNAWAKTNSFTEIVSTDAFNMLAAKTGYSVEKLLECIVRLNKGFGGRTILSYGSLCDEFTPTEVIRQMYGVNLQ |
| 14 | Canine coronavirus | Homo sapiens | CCoV-huPn-2018 | QWY12681.1 | SGLRKMAQPSGLVEPCIVRVSYGNNVLNGLWLGDEVICPRHVIASDTTRVINYENEMSSVRLHNFSVSKNNVFLGVVSAKYKGVNLVLKVNQVNPNTPEHKFKSIKAGESFNILACYEGCPGSVYGVNMRSQGTIKGSFIAGTCGSVGYVSENATLYFVYMHHLELGNGSHVGSNLEGEMYGGYEDQPSMQLEGTNVMSSDNVVAFLYAALINGERWFVTNTSMSLESYNTWAKTNSFTELSSTDAFSMLAAKTGQSVEKLLDSIVRLNKGFGGRTILSYGSLCDEFTPTEVIRQMYGVNLQ |
| 15 | Lucheng Rn rat coronavirus | Rattus norvegicus | RtCoV_LRNV | YP_009336483.1 | SGFKKIAQPSGLVEPCVVKVTYLNSYLNGVWLGDQVYAPRHVIASDVTKIVDYDTEQNLVRSHNFSISRGNSYLTVKGFRFEGCNVVISVVEVNPFTPEHKFDTLKPGDNFNILACYDGIPSGVYGVTLRHNSTIKGSFVNGTCGSPGYVISNGVIKFCYLHQMELGSGAHVGSDFNGKMYGGYQDQARIQVEGANKLITENVIAFFYAALLNGERWWCSKDSVCVTNFNSWAADNHYTMLSTTDVFNLVASKTGVSVEQVLAAIISYAKGFGHRTILGYASINDEYTITEVMQQMFGVQLQ |
| 16 | Human coronavirus 229E | Homo sapiens | 229E | AGT21358.1 | STLQGLRKMAQPSGFVEKCVVRVCYGNTVLNGLWLGDIVYCPRHVIASNTTSAIDYDHEYSIMRLHNFSIISGTAFLGVVGATMHGVTLKIKVSQTNMHTPRHSFRTLKSGEGFNILACYDGCAQGVFGVNMRTNWTIRGSFINGACGSPGYNLKNGEVEFVYMHQIELGSGSHVGSSFDGVMYGGFEDQPNLQVESANQMLTVNVVAFLYAAILNGCTWWLKGDKLFVEHYNEWAQANGFTAMNGEDAFSILAAKTGVCVERLLHAIQVLNNGFGGKQILGYSSLNDEFSINEVVKQMFGVNFQGP |
| 17 | Human coronavirus NL63 | Homo sapiens | NL63 | WDE19256.1 | STLQSGLKKMAQPSGCVERCVVRVCYGSTVLNGVWLGDTVTCPRHVIAPSTTVLIDYDHAYSTMRLHNFSVSHNGVFLGVVGVTMHGSVLRIKVSQSNVHTPKHVFKTLKPGDSFNILACYEGIASGVFGVNLRTNFTIKGSFINGACGSPGYNVRNDGTVEFCYLHQIELGSGAHVGSDFTGSVYGNFDDQPSLQVESANLMLSDNVVAFLYAALLNGCRWWLRSTRVNVDGFNEWAMANGYTSVSSVECYSILAAKTGVSVEQLLASIQHLHEGFGGKNILGYSSLCDEFTLAEVVKQMYGVNFQGP |
| 18 | Scotophilus bat coronavirus 512 | Bat | BtCoV-S512 | YP_001351683.1 | AGLRKMAQPSGIVEGCIVRVSYGNLTLNGLWLGDTVICPRHVIASNTTNVIDYDHAMSLVRLHNFSISSGNMFLGVISASMRGTLLHIKVNQSNVNTPNYTYKVLKPGDSFNILACYDGSAAGVYGVNMRTNYTIRGSFISGACGSPGYNINNGVVEFCYMHHLELGSGCHVGSDMDGTMYGKYEDQPTLQIEGASNLVTENVCSWLYGALINGDRWWLSSVSVGVDTYNEWALRNGMTALKNVDCFSLLVAKTGVDVGRLLASIQKLHGNFGGKSILGCTSLCDEFTLSEVVKQMYGVTLQ |
| 19 | BtMr-AlphaCoV/SAX2011 | Myotis ricketti | BtCoV_SAX2011 | YP_009199608.1 | SGLKKMAQPSGIVEPCVVRVSYNSTVLNGLWLGDTVYCPRHIIASNTNAVIDYDHAYTVMRLHNFSISAGNIYLGVVSATMHGANLHIKVNQSNVHTPVHSFRTVKPGEMFNILACYDGTPAGVYSVNLRTNHTIKGSFINGACGSPGYNIVNGTVEFVYMHQIELGSGCHVGSSFDGKIYGNYEDQPTLQIEGSANLVTDNVVAFLYGALLNGITWWLSPSRITVEAFNEWASVSGYTPLQSADGYSILAAKTGVEVERILSSIQKHANGFGGRNVLGYSALTDDFTLSEVVKQMFGVNLQ |
| 20 | BtRf-AlphaCoV/HuB2013 | Rhinolophus ferrumequinum | BtCoV_Rf2013 | YP_009199789.1 | SGLRKMAQPSGVVERCVVRVCYGNMVLNGLWLGDTVICPRHIIASSTSSIIDYEHQYSVMRLHNFSVSVGNVFLGVVGVTMKGTNLHIKVNQTNVHTPEHTFKTLKQGDSFNILACYDGVPSGVYGVTLRTNNTIRGSFINGACGSPGYNINNGKVEFCYLHQLELGSGCHVGSNLDGVMYGGFEDQPTLQVEGANNLVTINVIAFLYGALLNGITWFLSNDRVTVESFNEWASVNGYTDCGSIDCFAMLAAKTNVDVQRILAAVQRLHKGFGGKNILGFTTLTDEFTVTEVIKQMYGVSLQ |
| 21 | Bat coronavirus HKU10 | Hipposideros pomona | BtCoV_HKU10 | WCZ55949.1 | SGLRKMAQPSGVVEKCVVRVCYGNMALNGLWLGDVVICPRHVIASNTNALIDYEHEISVMRLHNFSVSVGNVFLGVISATMKGANLHIKVNQTNVNTPEHSFKTIKQGESFNILACYDGMPSGVYGVTMRNNYTIRGSFITGACGSPGYNVTNGKVEFCYLHQLELGSGCHVGSNLEGVMYGCYEDQPTLQIEGVNHLVTPNVIAFLYGALLNGITWWLNSDKVSVESFNEWALNNGFTTCGNLDCFNMLSAKTGVDVQRLLAAVQRLHNSFGGKNILGYTTLTDEFTVGEVIKQMYGVTLQ |
| 22 | Porcine epidemic diarrhea virus | Pig | PEDV | NP_598309.2 | AGLRKMAQPSGVVEKCIVRVCYGNMALNGLWLGDIVMCPRHVIASSTTSTIDYDYALSVLRLHNFSISSGNVFLGVVSATMRGALLQIKVNQNNVHTPKYTYRTVRPGESFNILACYDGAAAGVYGVNMRSNYTIRGSFINGACGSPGYNINNGTVEFCYLHQLELGSGCHVGSDLDGVMYGGYEDQPTLQVEGASSLFTENVLAFLYAALINGSTWWLSSSRIAVDRFNEWAVHNGMTTVGNTDCFSILAAKTGVDVQRLLASIQSLHKNFGGKQILGHTSLTDEFTTGEVVRQMYGVNLQ |
| 23 | Bat coronavirus 1A | Bat | BtCoV-MiCoV1 | YP_001718604.1 | AGLRKFAQPSGVIEHCIVRVSYGNMVLNGLWLGDEVICPRHVIASSINSAIDYDHEYTMMRLHNFSVSSGNLFIGVVSAKMRGASLVIKVNQNNPHTPKHVFKTLRAGDAFNILACYDGVPSGVYGTILRHNKTIRGSFINGACGSPGFNINGDTVEFVYLHQLELGSGCHVGSNMEGVMYGGFDDQPSLQIEGADCLVTVNVIAFLYGAILNGCTWFLSNERVSAEVFNGWAHDNNFTDVGSFDCFNILAAKTGVDVQRILASIQKLAKGFGGRNIIGYASLTDEFTVSEVVKQMYGVSLQ |
| 24 | Miniopterus bat coronavirus HKU8 | Bat | Bat_HKU8 | YP_001718611.1 | SGLKKMAQPSGIVEKCVVRVCYGNMTLNGLWLGDIVVCPRHVMASSTTNTIDYEYEYSMMRLHNFSVSVGNVFLGVNGVTMEGVNLHIKVNQSNPHTPKHTFRTLKPGDSFNILACYDGTPAGVYGITMRPNYTIRGSFINGACGSPGYNVVNGNVEFCYLHQLELGSGCHVGSNFDGIMYGNFQDQPSLQIEGADQLVTPNVVAFLYGALLNGVNWFVSPERLSVEAFNEWAHNNGFTDMSGAECFTMLAAKTGVDVQRVLASIQKIAKSFGGRNILGFTSLTDEFTAAEVIKQMYGVNLQ |

## 2. Protein amino acid conservation comparisons


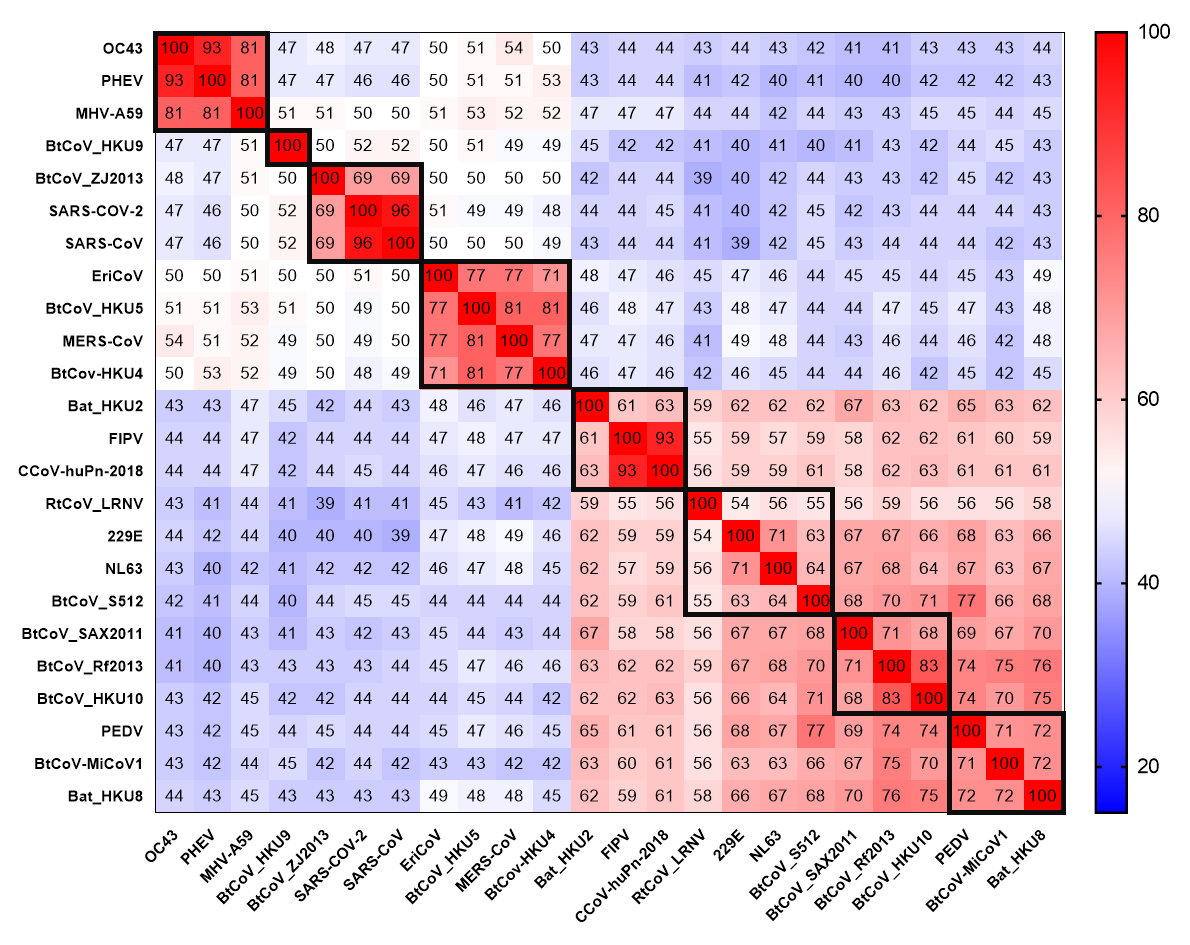


**Figure S1.** 24 high-risk CoV Mpros clustering diagram. Different categories of conserved levels were distinguished using two colors (red: conserved; blue: variable). Four categories of *beta*-CoVs (OC43~Bat_HKU4) and four categories of *alpha*-CoVs (Bat_HKU2~Bat_HKU8) were marked with a black box.

## 3. Protein purification


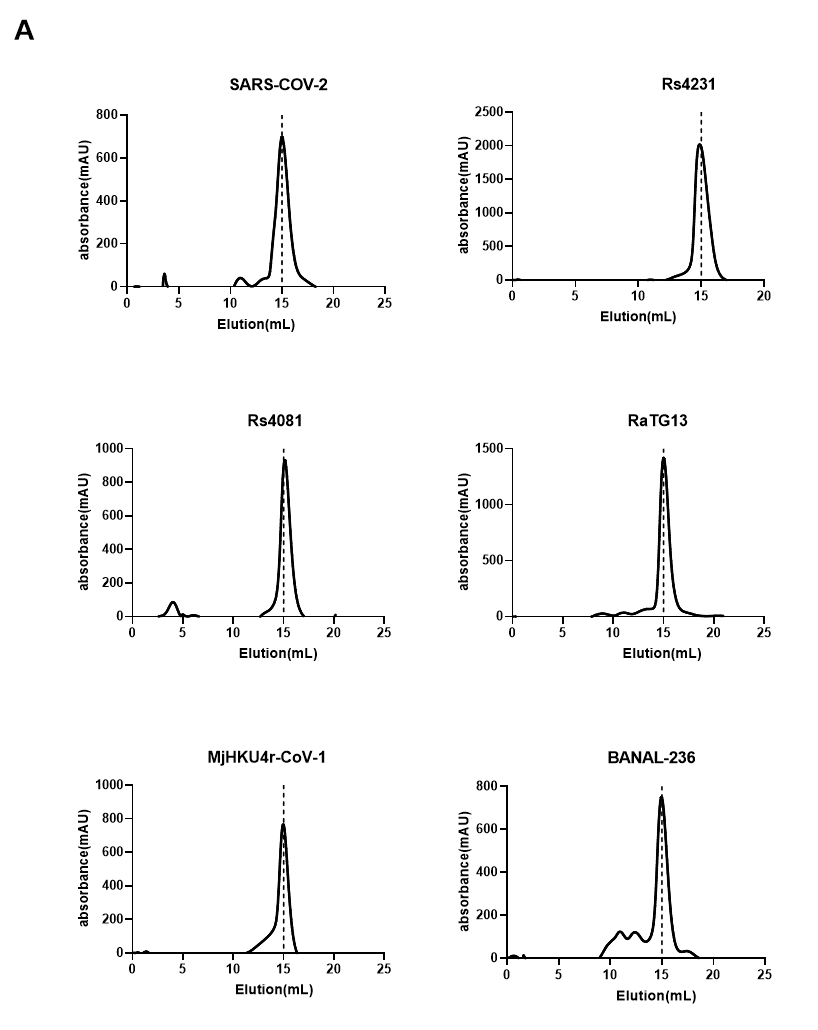


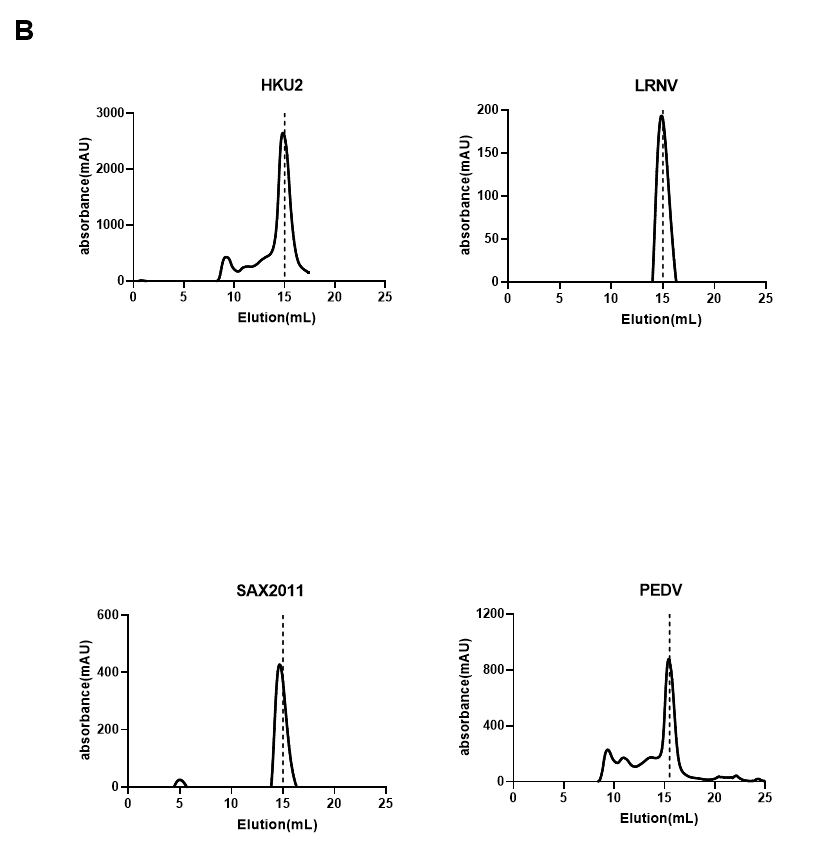


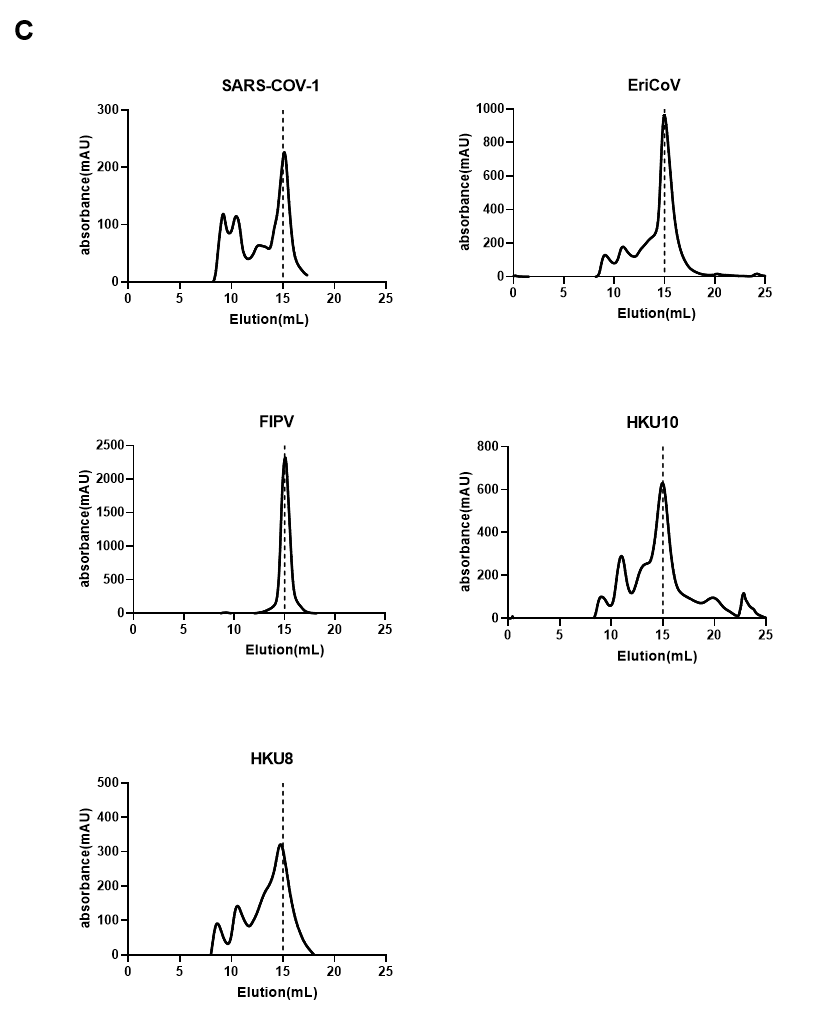


**Figure S2:** Collect the protein purified by HisTrap FF column, and then purify it using a Superdex 200 column from Cytiva. The standard elution position is indicated by a dashed line in the figure. The protein samples corresponding to the peak tip are collected.

## 4. Synthesis procedures

**Scheme S1: Synthesis of Compound 1**

**3-(*tert*-butyl)-6-(ethylthio)-1-(2,4,5-trifluorobenzyl)-1,3,5-triazine-2,4(1*H*,3*H*)-dione *(1b)***

A mixture of 3-(tert-butyl)-6-(ethylthio)-1,3,5-triazine-2,4(1*H*,3*H*)-dione (5750 mg, 25.0 mmol), potassium carbonate (6932 mg, 50.0 mmol), and **1a** (8464 mg, 37.6 mmol) in MeCN (30 mL) was stirred at 85°C for 16 h. The reaction mixture was cooled to room temperature and then diluted with water and EtOAc. The aqueous layer was extracted with EtOAc. The organic layer was washed with water and brine, dried over Na_2_SO_4_, and concentrated under reduced pressure. The residue was purified by silica gel column chromatography (PE: EtOAc = 3:1) to afford **1b** (8600 mg, 89%) as a white solid. MS (ESI): 318.0 [M+H-56]^+^.

**6-(ethylthio)-1-(2,4,5-trifluorobenzyl)-1,3,5-triazine-2,4(1*H*,3*H*)-dione (1c)**

A mixture of **1b** (6700 mg, 17.9 mmol) in THF (30 mL) and TFA (30 mL) was stirred at room temperature for 6 h. Concentration under reduced pressure afforded the residue, crude **1c** (7800 mg), as a white solid. MS (ESI): 318.0 [M+H]^+^.

**6-(ethylthio)-3-((1-methyl-1*H*-1,2,4-triazol-3-yl)methyl)-1-(2,4,5-trifluorobenzyl)-1,3,5-triazine-2,4(1*H*,3*H*)-dione *(1d)***

A mixture of **1c** (7800 mg, 24.6 mmol), potassium carbonate (10.2 g, 73.8 mmol), and 3-(chloromethyl)-1-methyl-1*H*-1,2,4-triazole hydrochloride (3882 mg, 29.5 mmol) in DMF (60 mL) was stirred at 60°C for 16 h. The reaction mixture was cooled to room temperature and then diluted with water and EtOAc. The aqueous layer was extracted with EtOAc. The organic layer was washed with water and brine, dried over Na_2_SO_4_, and concentrated under reduced pressure. The residue was purified by silica gel column chromatography (DCM: MeoH = 20:1) to afford **1d** (5200 mg, 50%) as a white solid. MS (ESI): 413.0 [M+H]^+^.

**(*E*)-6-((6-chloro-2-methyl-2*H*-indazol-5-yl)imino)-3-(pyridin-3-yl)-1-(2,4,5-trifluorobenzyl)-1,3,5-triazinane-2,4-dione *(1)***

LHMDS (0.73 mL, 0.73 mmol; 1.0 M in THF) was added dropwise to a solution of **1d** (150 mg, 0.37 mmol) and 6-chloro-2-methyl-2*H*-indazol-5-amine (80 mg, 0.43 mmol) in THF (20 mL) at 0°C. The reaction mixture was stirred at 0°C for 2 h. The reaction was quenched with aqueous NH_4_Cl, and the aqueous layer was extracted with EtOAc. The organic layer was washed with brine, dried over Na_2_SO_4_, and concentrated under reduced pressure. The residue was purified by silica gel column chromatography (DCM: MeoH = 20:1) to afford **1** (37.1 mg, 19%) as a white solid. MS (ESI): 532.0 [M+H]^+^. ^1^H NMR (400 MHz, DMSO-*d*_6_, DCl in D_2_O) δ 9.54 (s, 1H), 8.48 (s, 1H), 7.78 (s, 1H), 7.69-7.58 (m, 2H), 7.49 (s, 1H), 5.29 (s, 2H), 5.09 (s, 2H), 4.20 (s, 3H), 3.96 (s, 3H).

**Scheme S2. Synthesis of compound 2**

**(5-chloro-2,4-difluorophenyl)methanol *(2a2)***

To a solution of **2a1** (5.0 g, 26.0 mmol) in THF (50 mL) was added borane-tetrahydrofuran complex (52 mL, 52.0 mmol) at room temperature. The reaction mixture was stirred at 60°C for 3 h. The reaction was cooled to room temperature and then quenched with MeOH (50 mL). The reaction mixture was stirred at 60°C for 1 h. After concentration under reduced pressure, the residue was purified by silica gel column chromatography (PE : EtOAc = 5:1) to afford **2a2** (4.4 g, 94%) as a white solid. MS (ESI): 161.4 [M-18+H]^+^.

**1-(bromomethyl)-5-chloro-2,4-difluorobenzene *(2a)***

To a solution of **2a2** (4.4 g, 24.6 mmol) in DCM (50 mL) was added triphenylphosphine (9.68 g, 36.9mmol) and carbon tetrabromide (8.97 g, 27.1 mmol) at 0°C. The reaction mixture was stirred at room temperature for 4 h, and then diluted with water and DCM. The aqueous layer was extracted with DCM. The organic layer was dried over Na_2_SO_4_ and concentrated under reduced pressure. The residue was purified by silica gel column chromatography (PE: EtOAc = 10:1) to afford **2a** (5.6 g, 94%) as a pale-yellow oil. ^1^H NMR (400 MHz, CDCl_3_) *δ* 7.50-7.43 (m, 1H), 6.96-

6.90 (m, 1H), 4.42 (d, *J* = 0.9 Hz, 2H).

**(*E*)-1-(5-chloro-2,4-difluorobenzyl)-6-((6-chloro-2-methyl-2*H*-indazol-5-yl)imino)-3-((1-methyl-1*H*-1,2,4-triazol-3-yl)methyl)-1,3,5-triazinane-2,4-dione *(2)***

**2** was prepared from **2a** in a manner similar to that for **1**. White solid. MS (ESI) m/z:547.7[M+H]^+^.^1^H NMR (400 MHz, CD_3_OD) δ 8.36 (s, 1H), 8.19 (s, 1H), 7.75-7.69 (m, 2H), 7.41 (s, 1H), 7.20 (t, *J* = 9.5 Hz, 1H), 5.36 (s, 2H), 5.12 (s, 2H), 4.21 (s, 3H), 3.91 (s, 3H).

**Scheme S3. Synthesis of compound 3**

**3-bromo-5-((tetrahydrofuran-3-yl)oxy)pyridine *(3f2)***

To a solution of **3f1** (5.0 g，28.7 mmol), 3-Hydroxytetrahydrofuran (2.53 g，28.7 mmol), and Triphenylphosphine (11.3 g，43.1 mmol) in THF (100 mL) was added diisopropyl azodicarboxylate (8.71 g，43.1 mmol) at 0°C. The reaction mixture was stirred at room temperature for 16 h. After concentration under reduced pressure, the residue was purified by silica gel column chromatography (PE: EtOAc = 3:1) to afford **3f2** (7.7 g, 92%) as yellow oil. MS (ESI) m/z: 244.0[M+H]^+^.

**(5-((Tetrahydrofuran-3-yl)oxy)pyridin-3-yl)boronic acid *(3f)***

To a solution of **3f2** (6.4 g, 26.2 mmol), bis(pinacolato)diboron (9.98 g, 39.3 mmol), potassium acetate（7.71 g, 78.6 mmol）and 1,1'-bis(diphenylphosphino)ferrocene]-dichloropalladium(II)（1.92 g, 2.62 mmol） in 1,4-Dioxane (100 mL) was stirred under an nitrogen atmosphere at 80°C for 16 h. The reaction mixture was cooled to room temperature and stirred another 1h in the presence of PE (500mL), then filter through celite, wash the filter cake with PE, after concentration under reduced pressure, the residue was purified by C18 reversed-phase column chromatography (MeCN/ 0.5% Formic acid water gradient, 0−15% MeCN) to afford **3f** (8.0g, 69%) as yellow solid. MS (ESI): 210.1 [M+H]^+^.

**(S)-1-(5-chloro-2,4-difluorobenzyl)-6-(ethylthio)-3-(5-((tetrahydrofuran-3-yl)oxy)pyridin-3-yl)-1,3,5-triazine-2,4(1*H*,3*H*)-dione *(3d)***

A mixture of **3e** (2.0 g，6.0 mmol), **3f** (3.76 g，18.0 mmol), cupric acetate (92.18 g，12.0 mmol), triethylamine (3.04 g，30.0 mmol) and 4A molecular sieve (13.2 g，30.0 mmol) in DMF (50 mL) was stirred under an oxygen atmosphere at 60°C for 16 h. The reaction mixture was cooled to room temperature and then diluted with water and EtOAc. The aqueous layer was extracted with EtOAc. The organic layer was washed with water and brine, dried over Na_2_SO_4_, and concentrated under reduced pressure. The residue was purified by silica gel column chromatography (DCM:MeOH = 10:1) to afford **3d** (7.2 g, 79%) as a pale-yellow solid. MS (ESI) m/z: 497.0[M+H]^+^.

**(*S*,*E*)-1-(5-Chloro-2,4-difluorobenzyl)-6-((6-chloro-2-methyl-2*H*-indazol-5-yl)imino)-3-(5-((tetrahydrofuran-3-yl)oxy)pyridin-3-yl)-1,3,5-triazinane-2,4-dione *(3)***

**3** was prepared from **3d** in a manner similar to that for **1**. White solid. MS (ESI): 615.9 [M+H]^+^. ^1^H NMR (400 MHz, CD_3_OD) *δ* 8.33 (s, 1H), 8.25 (s, 1H), 8.18 (s, 1H), 7.81 (t, *J* = 7.8 Hz, 1H), 7.71 (s, 1H), 7.69-7.67 (m, 1H), 7.45 (br, 1H), 7.18 (t, *J* = 9.5 Hz, 1H), 5.35 (s, 2H), 5.11 (d, *J* = 1.4 Hz, 1H), 4.18 (s, 3H), 3.95 (d, *J* = 3.1 Hz, 3H), 3.87 -3.83 (m, 1H), 2.32-2.23 (m, 1H), 2.16-2.09 (m, 1H).

**Scheme S4. Synthesis of compound 4**

**(*E*)-1-(5-chloro-2,4-difluorobenzyl)-6-((6-chloro-2-methyl-2*H*-indazol-5-yl)imino)-3-(5-(2-methoxyethoxy)pyridin-3-yl)-1,3,5-triazinane-2,4-dione *(4)***

**4** was prepared from **2a** in a manner similar to that for **3**. MS (ESI) m/z: 604.0[M+H]^+^. ^1^H NMR (400 MHz, CD_3_OD) δ 8.26 (d, *J* = 2.6 Hz, 1H), 8.18-8.10 (m, 2H), 7.94-7.63 (m, 3H), 7.52-7.50 (m, 1H), 7.16 (s, 1H), 5.33 (s, 2H), 4.17-4.15 (m, 2H), 4.14 (s, 3H), 3.72-3.70 (m, 2H), 3.37 (s, 3H).

**Scheme S5. Synthesis of compound 5**

**4-(4,5-difluoro-2-methylphenoxy)benzonitrile *(5a2)***

To a solution of **5a1** (2.00 g, 9.70 mmol) in NMP (20 mL) was added 4-cyanophenol (2.12 g, 17.8 mmol), copper(I) chloride (440 mg, 4.45 mmol), cesium carbonate (5.80g, 17.8 mmol) and 2,2,6,6-tetramethyl-3,5-heptanedione (1.80 g, 9.79 mmol) at room temperature. The reaction mixture was stirred under a nitrogen atmosphere at 120°C for 12 h, and then diluted with water and EtOAc. The aqueous layer was extracted with EtOAc. The organic layer was washed with water and brine, dried over Na_2_SO_4_, and concentrated under reduced pressure. The residue was purified by silica gel column chromatography (PE: EtOAc = 100:1) to afford **5a2** (1.20 g, 50%) as a yellow oil. MS (ESI) m/z: 245.0 [M]^+^.

**4-(2-(bromomethyl)-4,5-difluorophenoxy)benzonitrile *(5a)***

To a solution of **5a2** (1.20 g, 4.9 mmol) in carbon tetrabromide (15 mL) was added 2,2'-azobis(2-methylpropionitrile) (140 mg, 0.88 mmol) and *N*-bromosuccinimide (940 mg, 5.28 mmol) at 0°C. The reaction mixture was stirred under a nitrogen atmosphere at stirred at 80°C for 2 h and then the reaction mixture concentrated under reduced pressure and purified by silica gel column chromatography (PE: EA = 10:1) to afford **5a** (1.0 g, 63%) as yellow oil. MS (ESI) m/z: 323.0 [M]^+^.

**(*E*)-4-(2-((6-((6-chloro-2-methyl-2*H*-indazol-5-yl)imino)-3-((1-methyl-1*H*-1,2,4-triazol-3-yl)methyl)-2,4-dioxo-1,3,5-triazinan-1-yl)methyl)-4,5-difluorophenoxy)-benzonitrile *(5)***

**5** was prepared from **5a** in a manner similar to that for **1** (18%) as yellow solid. MS (ESI) m/z: 631.0[M+H]^+^. ^1^H NMR (400 MHz, CD_3_OD) δ 8.33 (s, 1H), 8.11 (s, 1H), 7.68 (s, 1H), 7.60-7.49 (m, 3H), 7.09-7.04 (m, 4H), 5.25 (s, 2H), 5.04-5.02 (m, 2H), 4.19 (s, 3H), 3.89 (s, 3H).

**Scheme S6. Synthesis of compound 6**

**5-(4-chloro-5-fluoro-2-methylphenoxy)thiophene-2-carbonitrile *(6a2)***

A mixture of **6a1** (1.0 g，6.2 mmol), cesium carbonate (3.03 g, 9.3 mmol), and 5-nitrothiophene-2-carbonitrile (0.67 g，4.34 mmol) in DMF (60 mL) was stirred under a nitrogen atmosphere at room temperature for 16 h. After concentration under reduced pressure, the residue was purified by silica gel column chromatography (DCM: MeOH = 20:1) at 60°C for 16 h. The reaction mixture was diluted with water and EtOAc, and the aqueous layer was extracted with EtOAc. The organic layer was washed with water and brine, dried over Na_2_SO_4_, and concentrated under reduced pressure. The residue was purified by silica gel column chromatography (PE: EA = 5:1) to afford **6a2** (5100 mg, 30%) as yellow oil. MS (ESI) m/z: 268.2[M+H]^+^.

**(E)-5-(4-chloro-2-((6-((6-chloro-2-methyl-2*H*-indazol-5-yl)imino)-3-((1-methyl-1*H*-1,2,4-triazol-3-yl)methyl)-2,4-dioxo-1,3,5-triazinan-1-yl)methyl)-5-fluoro-phenoxy)thiophene-2-carbonitrile *(6)***

**6** was prepared from **6a** in a manner similar to that for **1**. yellow solid. MS (ESI) m/z: 653.1 [M+H]^+^. ^1^H NMR (400 MHz, DMSO-*d*_6_) δ 11.04 (br, 1H), 8.43-8.21 (m, 2H), 7.85-7.61 (m, 3H), 7.49 (d, *J* = 9.8 Hz, 1H), 7.40-7.10 (m, 1H), 6.83 (d, *J* = 3.9 Hz, 1H), 5.24 (s, 2H), 4.91 (s, 2H), 4.15 (s, 3H), 3.81 (s, 3H).

**Scheme S7. Synthesis of compound 7**

**4-(2-(bromomethyl)-4-chloro-5-fluorophenoxy)benzonitrile *(7a)***

**7a** was prepared from **7a1** in a manner similar to that for **5a**. yellow solid. MS (ESI) m/z: 340.4 [M+H]^+^.

**(E)-4-(4-chloro-2-((6-((6-chloro-2-methyl-2*H*-indazol-5-yl)imino)-3-(5-fluoro-pyridin-3-yl)-2,4-dioxo-1,3,5-triazinan-1-yl)methyl)-5-fluorophenoxy)-benzonitrile *(7)***

**7** was prepared from **7a** in a manner similar to that for **3** as yellow solid. MS (ESI) m/z: 647.1 [M+H]^+^. ^1^H NMR (400 MHz, DMSO-*d*_6_) δ 8.66 (s, 1H), 8.41 (d, *J* = 41.1 Hz, 2H), 8.09-7.71 (m, 5H), 7.35-7.11 (m, 3H), 5.16 (s, 2H), 4.16 (s, 3H).

**Scheme S8. Synthesis of compound 8**

**(2-Amino-5-chloro-4-fluorophenyl)methanol *(8a2)***

To a solution of **8a1** (1.8 g, 10.3 mmol) in THF (10 mL) was added lithium borohydride (1.12 g, 51.5 mmol) at room temperature. The reaction mixture was stirred at room temperature for 12 h, and then diluted with water. The aqueous layer was extracted with EtOAc. The organic layer was dried over Na_2_SO_4_ and concentrated under reduced pressure to afford **8a2** (1.3 g, 85%) as white solid. MS (ESI): 176.1 [M+H]^+^.

**(5-Chloro-4-fluoro-2-iodophenyl)methanol *(8a3)***

To a solution of **8a2** (6.7 g, 370 mmol) in acetone (100 mL) was added concentrated hydrochloric acid (15 mL) at 0°C. The reaction mixture was stirred at 0°C for 1 h, and then the sodium nitrite solution (3.32 g, 48 mmol) was added. The reaction mixture was stirred at 0°C for 2 h, and then the sodium iodide solution (16.65 g, 111 mmol) was added. The reaction mixture was stirred at room temperature for 16 h, and then diluted with water. The aqueous layer was extracted with EtOAc. The organic layer was dried over Na_2_SO_4_ and concentrated under reduced pressure. The residue was purified by silica gel column chromatography (PE: EtOAc = 10:1) to afford **8a3** (4.1 g, 35%) as a pale-yellow solid. MS (ESI): 285.1 [M-H]^+^.

**(*E*)-6-((6-Chloro-2-methyl-2*H*-indazol-5-yl)imino)-1-(5-chloro-4-fluoro-2-iodobenzyl)-3-(5-methylpyridin-3-yl)-1,3,5-triazinane-2,4-dione *(8e)***

**8e** was prepared from **8a** in a manner similar to that for **3**.

**(*E*)-6-((6-Chloro-2-methyl-2*H*-indazol-5-yl)imino)-1-(5-chloro-4-fluoro-2-((3-fluorophenyl)thio)benzyl)-3-(5-methylpyridin-3-yl)-1,3,5-triazinane-2,4-dione *(8)***

To a solution of **8e** (135 mg, 0.21 mmol) in 1,4-dioxane (10 mL) was added 3-fluorothiophenol (29 mg, 0.23 mmol), potassium carbonate (86 mg, 0.62 mmol), cupric acetate (4 mg, 0.02 mmol) and *o*-phenanthroline (3.7 mg, 0.02 mmol) at room temperature. The reaction mixture was stirred under a nitrogen atmosphere at 110°C for 12 h. After concentration under reduced pressure, the residue was purified by silica gel column chromatography (DCM: MeOH = 20:1) and preparative HPLC to afford **8** (66.3 mg, 48%) as white solid. MS (ESI): 652.2 [M+H]^+^. ^1^H NMR (400 MHz, CD_3_OD) δ 8.76-8.41 (m, 2H), 8.16 (s, 1H), 8.03 (s, 1H), 7.80 (d, *J* = 7.3 Hz, 1H), 7.70 (s, 1H), 7.41 (s, 1H), 7.30-7.19 (m, 2H), 7.06 (d, *J* = 7.9 Hz, 1H), 7.01-6.96 (m, 1H), 6.91 (t, *J* = 8.5 Hz, 1H), 5.39 (s, 2H), 4.17 (s,3H), 2.46 (s, 3H).

**Scheme S9. Synthesis of compound 9**

***(E)*-6-((6-chloro-2-methyl-2*H*-indazol-5-yl)imino)-1-(5-chloro-4-fluoro-2-(4-fluorophenoxy)benzyl)-3-((1-methyl-1*H*-1,2,4-triazol-3-yl)methyl)-1,3,5-triazinane-2,4-dione *(9)***

**9** was prepared from **9a** in a manner similar to that for **1** as white solid. MS (ESI) m/z:640.1[M+H]^+^. ^1^H NMR (400 MHz, CD_3_OD) δ 8.35 (s, 1H), 8.19 (s, 1H), 7.73 (s, 1H), 7.65 (d, *J* = 8.1 Hz, 1H), 7.43 (s, 1H), 7.12 (d, *J* = 6.0 Hz, 4H), 6.69 (d, *J* = 10.2 Hz, 1H), 5.39 (s, 2H), 5.10 (s, 2H), 4.21 (s, 3H), 3.91 (s, 3H).

**Scheme S10 Synthesis of compound 10**

***(E)*-1-(5-chloro-2-(3-chlorophenoxy)-4-fluorobenzyl)-6-((6-chloro-2-methyl-2*H*-indazol-5-yl)imino)-3-((1-methyl-1*H*-1,2,4-triazol-3-yl)methyl)-1,3,5-triazinane-2,4-dione *(10)***

**10** was prepared from **10a** in a manner similar to that for **1** as white solid. MS (ESI) m/z: 656.1[M+H]^+^. ^1^H NMR (400 MHz, DMSO-*d*6) δ 8.31 (dd, *J* = 57.0, 37.1 Hz, 2H), 7.83-7.36 (m, 3H), 7.33-7.20 (m, 1H), 7.16-6.98 (m, 4H), 5.17 (d, *J* = 47.7 Hz,2H), 4.84 (d, *J* = 12.0 Hz, 2H), 4.12 (d, *J* = 22.2 Hz, 3H), 3.76 (d, *J* = 13.2 Hz, 3H).

**Scheme S11 Synthesis of compound 11**

***(E)*-1-(5-chloro-2-(3,4-difluorophenoxy)-4-fluorobenzyl)-6-((6-chloro-2-methyl-2*H*-indazol-5-yl)imino)-3-((1-methyl-1*H*-1,2,4-triazol-3-yl)methyl)-1,3,5-triazinane-2,4-dione *(11)***

**11** was prepared from **11a** in a manner similar to that for **1** as white solid. MS (ESI) m/z:658.1[M+H]^+^. ^1^H NMR (400 MHz, CD_3_OD) δ 8.34 (s, 1H), 8.13 (s, 1H), 7.78-7.62 (m, 2H), 7.09 (d, *J* = 45.0 Hz, 3H), 6.83 (d, *J* = 9.9 Hz, 2H), 5.35 (s, 2H), 5.10 (s, 2H), 4.21 (s, 3H), 3.91 (s, 3H).

**Scheme S12. Synthesis of compound 12**

***(E)*-6-((6-chloro-2-methyl-2*H*-indazol-5-yl)imino)-1-(5-chloro-4-fluoro-2-((5-fluoropyridin-2-yl)oxy)benzyl)-3-((1-methyl-1*H*-1,2,4-triazol-3-yl)methyl)-1,3,5-triazinane-2,4-dione *(12)***

**12** was prepared from **12a** in a manner similar to that for **1** as white solid. MS (ESI) m/z:641.0[M+H]^+^. ^1^H NMR (400 MHz, Chloroform-*d*) δ 8.04 (s, 1H), 7.76 (s, 1H), 7.60 (d, *J* = 7.9 Hz, 1H), 7.42 (s, 1H), 5.31 (s, 1H), 5.19 (s, 1H), 4.21 (s, 2H), 3.89 (s, 1H).

**Scheme S13. Synthesis of compound 13**

***(E)*-1-(5-chloro-2-((4,4-difluorocyclohexyl)oxy)-4-fluorobenzyl)-6-((6-chloro-2-methyl-2*H*-indazol-5-yl)imino)-3-((1-methyl-1*H*-1,2,4-triazol-3-yl)methyl)-1,3,5-triazinane-2,4-dione *(13)***

**13** was prepared from **13a** in a manner similar to that for **1**. MS (ESI) m/z: 664.1 [M+H]^+^. ^1^H NMR (400 MHz, CD_3_OD) δ 8.32-8.08 (m, 2H), 7.80-7.63 (m, 1H), 7.45 (d, *J* = 8.0 Hz, 1H), 7.13-6.98 (m, 2H), 5.26 (s, 2H), 5.11 (s, 2H), 4.66 (d, *J* = 17.2 Hz, 1H), 4.18 (d, *J* = 14.0 Hz, 3H), 3.88 (d, *J* = 9.6 Hz, 3H), 2.23-2.12 (m, 2H), 2.10-1.98 (m, 6H).

**Scheme S14. Synthesis of compound 14**

***(E)*-6-((6-chloro-2-methyl-2*H*-indazol-5-yl)imino)-1-(5-chloro-4-fluoro-2-((5-(trifluoromethyl)thiazol-2-yl)oxy)benzyl)-3-((1-methyl-1*H*-1,2,4-triazol-3-yl)methyl)-1,3,5-triazinane-2,4-dione *(14)***

**14** was prepared from **14a** in a manner similar to that for **1** as pale-yellow solid. MS (ESI) m/z: 697.2 [M+H]^+^. ^1^H-NMR (400 MHz, DMSO-*d*6) δ 8.42 (d, *J* = 27.4 Hz, 1H), 8.21 (s, 1H), 7.96-7.90 (m, 1H), 7.82 (d, *J* = 5.4 Hz, 1H), 7.66 (d, *J* = 12.8 Hz, 1H), 7.10 (t, *J* = 51.2 Hz, 3H), 5.31 (s, 3H), 4.90 (d, *J* = 6.8 Hz, 2H), 4.15 (d, *J* = 23.3 Hz, 3H), 3.83-3.76 (m, 3H).

**Scheme S15. Synthesis of compound 15**

***(E)*-4-((4-chloro-2-((6-((6-chloro-2-methyl-2*H*-indazol-5-yl)imino)-3-(5-methylpyridin-3-yl)-2,4-dioxo-1,3,5-triazinan-1-yl)methyl)-5-fluoro-phenyl)thio)benzonitrile *(15)***

**15** was prepared from **8a** in a manner similar to that for **8** as white solid. MS (ESI) m/z:659.2[M+H]^+^. ^1^H NMR (400 MHz, CD_3_OD) δ 8.50 (s, 1H), 8.13 (s, 1H), 7.94-7.84 (m, 3H), 7.69 (s, 1H), 7.49 (d, *J* = 8.7 Hz, 1H), 7.36 (d, *J* = 10.7 Hz, 2H), 7.21 (d, *J* = 8.2 Hz, 3H), 5.37 (s, 2H), 4.17 (s, 3H), 2.44 (s, 3H).

**Scheme S16. Synthesis of compound 16**

***(E)*-6-((6-chloro-2-methyl-2*H*-indazol-5-yl)imino)-1-(5-chloro-4-fluoro-2-((4-fluorophenyl)thio)benzyl)-3-(5-methylpyridin-3-yl)-1,3,5-triazinane-2,4-dione *(16)***

**16** was prepared from **8a** in a manner similar to that for **8** as white solid. MS (ESI) m/z:652.2[M+H]^+^. ^1^H NMR (400 MHz, CD_3_OD) δ 8.61 (s, 1H), 8.23-8.10 (m, 2H), 7.82 (d, *J* = 8.4 Hz, 1H), 7.71 (s, 1H), 7.65 (d, *J* = 7.3 Hz, 1H), 7.51 (s, 1H), 5.22 (s, 2H), 4.18 (s,3H), 2.49 (d, *J* = 2.6 Hz, 3H).

**Scheme S17. Synthesis of compound 17**

**methyl 3-chloro-4-fluoro-5-((2-methoxyethyl)thio)benzoate *(17a2)***

**17** was prepared from **17a1** in a manner similar to that for **3f2.**

**(3-chloro-4-fluoro-5-((2-methoxyethyl)thio)phenyl)methanol *(17a3)***

**17** was prepared from **17a2** in a manner similar to that for **2a2.**

**3-chloro-4-fluoro-5-((2-methoxyethyl)thio)benzyl methanesulfonate *(17a)***

To a solution of **17a3** (1 g, 4.00 mmol) in DCM (30 mL) was added triethylamine (2.02 g, 0.02 mol) and methylsulfonyl chloride (0.6 g, 5.2 mmol) at 0°C. The mixture was stirred at 0°C for 1 h. and then diluted with water and EtOAc. The aqueous layer was extracted with EtOAc. The organic layer was washed with water and brine, dried over Na_2_SO_4_, and concentrated under reduced pressure to afford**17a** as pale-yellow oil (1 g, yield: 75%). MS (ESI) m/z: 350.9 [M+Na]^+^.

**3-(*tert*-butyl)-1-(3-chloro-4-fluoro-5-((2-methoxyethyl)thio)benzyl)-6-(ethylthio)-1,3,5-triazine-2,4(1*H*,3*H*)-dione *(17b)***

To a solution of **17a** (1 g, 3 mmol) in acetonitrile (30 mL) was added potassium carbonate (1.24 g, 9 mmol) and 3-(tert-butyl)-6-(ethylthio)-1,3,5-triazine-2,4(1*H*,3*H*)-dione (0.69 g, 3 mmol). The mixture was stirred at 60°C for 2 h. And then diluted with water and EtOAc. The aqueous layer was extracted with EtOAc. The organic layer was washed with water and brine, dried over Na_2_SO_4_, and concentrated under reduced pressure. The residue was purified by silica gel column chromatography (PE: EtOAc = 4:1) to afford **17b** as pale-yellow oil (1 g, yield: 73%). MS (ESI) m/z:484.0[M+Na]^+^.

***(E)*-6-((6-chloro-2-methyl-2*H*-indazol-5-yl)imino)-1-(3-chloro-4-fluoro-5-((2-methoxyethyl)thio)benzyl)-3-((1-methyl-1*H*-1,2,4-triazol-3-yl)methyl)-1,3,5-triazinane-2,4-dione *(17)***

**17** was prepared from **17b** in a manner similar to that for **1**. MS (ESI) m/z:620.2[M+H]^+^. ^1^H NMR (400 MHz, DMSO-*d*6) δ 11.05 (s, 1H), 9.55 (s, 1H), 8.34 (m, 2H), 7.73 (s, 1H), 7.31 (d, *J* = 144.5 Hz, 3H), 5.19 (t, *J* = 31.5 Hz, 2H), 4.92 (s, 2H), 4.15 (s, 3H), 3.80 (s, 3H), 3.51 (s, 2H), 3.19 (s, 5H).

**Scheme S18. Synthesis of compound 18 (swd0406)**

**6-((6-chloro-2-methyl-2*H*-indazol-5-yl)amino)-1-(3-chloro-4-fluoro-5-((3-fluorophenyl)thio)benzyl)-3-(5-methylpyridin-3-yl)-1,3,5-triazine-2,4(1*H*,3*H*)-dione *(18)***

**18** was prepared from **8a** in a manner similar to that for **8** as white solid. MS (ESI) m/z: 652.1 654.1 [M+H]^+^. ^1^H NMR (400 MHz, DMSO-*d*_6_) δ 11.17 (s, 0.55H), 9.60 (s, 0.45H), 8.55-8.20 (m, 3H), 7.93-7.28 (m, 6H), 7.18-7.08 (m, 3H), 5.31-5.11 (m, 2H), 4.16 (s, 3H), 2.35 (s, 3H).

**Scheme S19 Synthesis of compound 19 (swd061)**

**methyl 2-(benzyloxy)-4,5-difluorobenzoate *(19a2)***

To a solution of **19a1** (2 g, 0.01 mol) in DMF (20 mL) was add potassium carbonate (4.4 g, 0.03 mol) and benzyl bromide (2.18 g, 0.013 mol). The mixture was stirred at 70°C for 16 h. And then diluted with water and EtOAc. The aqueous layer was extracted with EtOAc. The organic layer was washed with water and brine, dried over Na_2_SO_4_, and concentrated under reduced pressure to afford white solid **19a2** (2.4 g). ^1^H NMR (400 MHz, DMSO-*d*_6_) δ 7.73 (dd, *J* = 10.8, 9.4 Hz, 1H), 7.62-7.23 (m, 6H), 5.17 (s, 2H), 3.77 (dd, *J* = 6.4, 1.0 Hz, 3H).

**1-(benzyloxy)-2-(bromomethyl)-4,5-difluorobenzene** *(****19a****)*

**19a** was prepared from **19a2** in a manner similar to that for **2a.** ^1^H NMR (400 MHz, DMSO-*d*_6_) δ 7.56 (dd, *J* = 10.9, 9.4 Hz, 1H), 7.52-7.43 (m, 2H), 7.43-7.21 (m, 4H), 5.17 (s, 2H), 4.60 (s, 2H).

***(E)*-1-(2-(benzyloxy)-4,5-difluorobenzyl)-6-((6-chloro-2-methyl-2H-indazol-5-yl)imino)-3-((1-methyl-1H-1,2,4-triazol-3-yl)methyl)-1,3,5-triazinane-2,4-dione** ***(19)***

**19** was prepared from **19a** in a manner similar to that for **1** as white solid**.** MS (ESI) m/z:620.0[M+H]^+^. ^1^H NMR (400 MHz, DMSO-*d*_6_) δ 8.33 (s, 1H), 8.24 (s, 1H), 7.66 (s, 1H), 7.54-7.16 (m, 8H), 5.26-5.11 (m, 4H), 4.88 (s, 2H), 4.13 (s, 3H), 3.78 (s, 3H).

**Scheme S20. Synthesis of compound 20(swd0098)**

**methyl 2-(bromomethyl)-4,5-difluorobenzoate *(20a2)***

**20a2** was prepared from **20a1** in a manner similar to that for **5a** as pale-yellow oil**.** MS (ESI) m/z:264.0[M]^+^.

***(E)*-6-((6-chloro-2-methyl-2H-indazol-5-yl)imino)-1-(4,5-difluoro-2-(phenoxymethyl)benzyl)-3-((1-methyl-1*H*-1,2,4-triazol-3-yl)methyl)-1,3,5-triazinane-2,4-dione *(20)***

**20** was prepared from **20a** in a manner similar to that for **2** as white solid. MS (ESI) m/z:620.0[M+H]^+^.^1^H NMR (400 MHz, DMSO-*d*_6_) δ 8.39-8.27 (m, 2H), 7.75-7.73 (m, 1H), 7.65 (s, 1H), 7.48 (s, 1H), 7.32-6.93 (m, 6H), 5.37-5.25 (m, 4H), 4.95 (s, 2H),4.18-4.13(m, 3H), 3.81 (s, 3H).

**Scheme S21. Synthesis of compound 21(swd0250)**

***(E)*-6-((6-chloro-2-methyl-2*H*-indazol-5-yl)imino)-1-(5-chloro-4-fluoro-2-iodobenzyl)-3-((1-methyl-1*H*-1,2,4-triazol-3-yl)methyl)-1,3,5-triazinane-2,4-dione *(21c)***

**21c** was prepared from **8c** in a manner similar to that for **1**.

***(E)*-6-((6-chloro-2-methyl-2*H*-indazol-5-yl)imino)-1-(4,5-difluoro-2-(*(E)*-styryl)benzyl)-3-((1-methyl-1*H*-1,2,4-triazol-3-yl)methyl)-1,3,5-triazinane-2,4-dione *(21)***

To a solution of styrene (63 mg, 0.607 mmol) and compound **21c** (139 mg, 0.212 mmol) in DMF (5 mL) was added triethylamine (184 mg, 1.82 mmol), palladium acetate (13.6 mg, 0.0607 mmol) and triphenylphosphine (18.5 mg, 0.0607 mmol) at room temperature. The reaction mixture was stirred under nitrogen atmosphere at 90°C for 16 h. And then diluted with water and EtOAc. The aqueous layer was extracted with EtOAc. The organic layer was washed with water and brine, dried over Na_2_SO_4_, and concentrated under reduced pressure. The residue was purified by silica gel column chromatography (PE: EtOAc = 1:1) to afford compound **21** as white solid. MS (ESI) m/z:616.2[M+Na]^+^. ^1^H NMR (400 MHz, CD_3_OD) δ 8.24 (s, 1H), 8.10 (s, 1H), 7.66 (s, 1H), 7.58-7.35 (m, 5H), 7.33-7.13 (m, 4H), 7.01 (d, *J* = 14.4 Hz, 1H), 5.40 (s, 2H), 5.04 (s, 2H), 4.14 (s, 3H), 3.80 (s, 3H).

**Scheme S22. Synthesis of compound 22 (swd0299)**

***(E)*-6-((6-chloro-2-methyl-2H-indazol-5-yl)imino)-1-(5-chloro-4-fluoro-2-(*(E)*-2-(thiazol-5-yl)vinyl)benzyl)-3-((1-methyl-1*H*-1,2,4-triazol-3-yl)methyl)-1,3,5-triazinane-2,4-dione *(22)***

**22** was prepared from **21b** in a manner similar to that for **21** as white solid. MS (ESI) m/z:639.1[M+H]^+^. ^1^H NMR (400 MHz, CD_3_OD) δ 8.87 (s, 1H), 8.34 (s, 1H), 8.13 (s, 1H), 7.91 (s, 1H), 7.67 (s, 1H), 7.62 (d, *J* = 7.4 Hz, 1H), 7.55 (d, *J* = 10.5 Hz, 1H), 7.42 (s, 1H), 7.35 (d, *J* = 15.6 Hz, 2H), 5.38 (s, 2H), 5.09 (s, 2H), 4.16 (s, 3H), 3.84 (s, 3H).

**Scheme S23. Synthesis of compound 23 (swd0480)**

**5-Bromopyridine-3-thiol *(23f2)***

A mixture of **23f1** (2.00 g, 6.40 mol) in TFA (10 mL) was stirred at 130°C for 1.5 h. The reaction mixture was concentrated under reduced pressure to afford **23f2** (1.5 g, 98%) as a black oil. MS (ESI): 190.1 [M+H]^+^.

**3-Bromo-5-(cyclopropylthio)pyridine *(23f3)***

To a solution of **23f2** (1.90 g, 0.01 mol) in 1,4-dioxane (20 mL) was added cyclopropylboronic acid (1.03 g, 0.012 mol), cupric acetate (3.63 g, 0.02 mmol) and triethylamine (6.07 g, 0.06 mmol) at room temperature. The reaction mixture was stirred under an oxygen atmosphere at 90°C for 16 h, and then filtered, the filtrate was concentrated under reduced pressure to afford **23f3** (300 mg, 10%) as a black oil. MS (ESI) : 232.0 [M+H]^+^.

**Methyl 5-cyano-2-methylbenzoate *(23a2)***

To a solution of **23a1** (3.85 g, 13.9 mmol) in DMF (50 mL) was added copper(I) cyanide (1.87 g, 20.8 mmol) and tetrakis(triphenylphosphine)palladium (0.8 g, 0.695 mmol) at room temperature. The reaction mixture was stirred at 100°C for 12 h. The reaction mixture was filtered, and the filtrate was concentrated under reduced pressure. The residue was purified by silica gel column chromatography (PE: EtOAc = 4:1) to afford **23a2** (1.9 g, 77%) as a brown solid. MS (ESI): 175 [M-18+H]^+^

**3-(Hydroxymethyl)-4-methylbenzonitrile *(23a3)***

To a solution of **23a2** (1.8 g, 10.3 mmol) in THF (10 mL) was added lithium borohydride (1.12 g, 51.5 mmol) at room temperature. The reaction mixture was stirred at room temperature for 12 h, and then diluted with water. The aqueous layer was extracted with EtOAc. The organic layer was dried over Na_2_SO_4_ and concentrated under reduced pressure to afford **23a3** (1.3 g, 85.44%) as a colorless oil.

**(*E*)-3-((6-((6-Chloro-2-methyl-2*H*-indazol-5-yl)imino)-3-(5-(cyclopropylthio)-pyridin-3-yl)-2,4-dioxo-1,3,5-triazinan-1-yl)methyl)-4-methylbenzonitrile *(23)***

**23** was prepared from **23b** in a manner similar to that for **3**. White solid. MS (ESI): 571.1 [M+H]^+^. ^1^H NMR (400 MHz, DMSO-*d_6_*) δ 8.55 (s, 1H), 8.38 (s, 2H), 7.97 (s, 1H), 7.89 (s, 2H), 7.68 (s, 2H), 7.42 (s, 1H), 5.20 (s, 2H), 4.16 (s, 3H), 2.42 (s, 3H), 2.36 (m, 1H), 1.13 (d, *J* = 5.6 Hz, 2H), 0.64 (d, *J* = 2.4 Hz, 2H).

**Scheme S24. Synthesis of compound 24 (SWD0496)**

**4-((5-chlorothiophen-2-yl)thio)-3-methylbenzonitrile *(24a2)***

To a solution of **24a1** (800 mg, 5.36 mmol) in 1,4-dioxane (15 mL) was added 2-bromo-5-chlorothiophene (1059 mg, 5.36 mmol), potassium carbonate (2223 mg, 16.08 mmol), cupric acetate (306 mg, 1.61 mmol) and *o*-phenanthroline (290 mg, 1.61 mmol) at room temperature. The reaction mixture was stirred under a nitrogen atmosphere at 120°C for 16 h. After concentration under reduced pressure, the residue was purified by silica gel column chromatography (PE: EtOAc = 10:1) to afford **24a2** (500 mg, 35.0%) as a yellow oil. MS (ESI): 265.1 [M+H]^+^.

**(*E*)-3-((6-((6-Chloro-2-methyl-2*H*-indazol-5-yl)imino)-3-(5-methylpyridin-3-yl)-2,4-dioxo-1,3,5-triazinan-1-yl)methyl)-4-((5-chlorothiophen-2-yl)thio)benzonitrile *(24)***

**24** was prepared from **24a2** in a manner similar to that for **3**as white solid. MS (ESI): 647.1 649.1 [M+H]^+^. ^1^H NMR (400 MHz, DMSO-*d*_6_) δ 11.26 (s, 0.6H), 9.71 (s, 0.4H), 8.36 (d, *J* = 75.1 Hz, 3H), 8.23-8.01 (m, 1H), 7.77 (t, *J* = 34.3 Hz, 3H), 7.49 (d, *J* = 3.3 Hz, 1H), 7.30 (s, 1H), 7.12 (d, *J* = 16.3 Hz, 2H), 5.26 (s, 2H), 4.15 (s, 3H), 2.36 (s, 3H).

**Scheme S25. Synthesis of compound 25 (swd0356)**

**3-((2-methoxypyridin-3-yl)methyl)-6-(1*H*-pyrazol-1-yl)-1,3,5-triazine-2,4(1*H*,3*H*)-dione *(25b)***

To a solution of **25a** (2.0 g, 14.5 mmol) and 1,1'-Carbonyldiimidazole (2.59 g, 16.0 mmol) in *N*, *N*-Dimethylacetamide (10 mL) was stirred at 0°C, the reaction mixture was warm to room temperature for 1 h. Then 1*H*-pyrazole-1-carboximidamide (1.60 g, 14.5 mmol) and DBU (2.43 g, 16.0 mmol) was added at 0°C, the reaction mixture was warm to room temperature for 17 h. Then 1*H*-pyrazole-1-carboximidamide (3.53 g，21.8 mmol) and DBU (3.31 g, 21.8 mmol) was added at 0°C, the reaction mixture was warm to room temperature for 2 h. Then 1*H*-pyrazole-1-carboximidamide (2.35 g，14.5 mmol) and DBU (2.21 g, 14.5 mmol) was added at 0°C, the reaction mixture was warm to room temperature for 2 h. After quenched with 2N HCl, the reaction mixture was stirred another 1h, while the white solid was precipitated, then filtered and washed the filter cake with H_2_O to afford **25b** (2.41g, 55%) as white solid. MS (ESI): 210.1 [M+H]^+^.

**6-((6-chloro-2-methyl-2*H*-indazol-5-yl)imino)-3-((2-methoxypyridin-3-yl)-methyl)-1,3,5-triazinane-2,4-dione *(25c)***

To a solution of **25b** (300 mg, 0.999 mmol) and 6-chloro-2-methyl-2*H*-indazol-5-amine (181 mg, 0.999 mmol) in NMP (5 mL) was added *p*-toluenesulfonic acid (596.0 mg, 0.999 mmol) at room temperature. The reaction mixture was stirred at 80°C for 2 h. The reaction was cooled to room temperature and then quenched with ice water, the solid was precipitated, then filtered and washed the filter cake with H_2_O to afford **25c** (299 mg, 72%) as brown solid. MS (ESI) m/z: 414.1[M+H]^+^.

**(*E*)-5-(4-chloro-2-((6-((6-chloro-2-methyl-2*H*-indazol-5-yl)imino)-3-((2-methoxy-pyridin-3-yl)methyl)-2,4-dioxo-1,3,5-triazinan-1-yl)methyl)-5-fluorophenoxy)-thiophene-2-carbonitrile (*25d*)**

A mixture of **25c** (267 mg, 0.645 mmol), **6a** (224 mg, 0.645 mmol), and DIPEA (167 mg, 1.29 mmol) in *N*, *N*-Dimethylacetamide (5 mL) was stirred at 60°C for 3 h. The reaction mixture was diluted with water and EtOAc, and the aqueous layer was extracted with EtOAc. The organic layer was washed with water and brine, dried over Na_2_SO_4_, and concentrated under reduced pressure. The residue was purified by silica gel column chromatography (DCM: MeOH = 10:1) to afford **25d** (95 mg, 21%) as yellow oil. MS (ESI) m/z: 679.1[M+H]^+^.

**(*E*)-5-(4-chloro-2-((6-((6-chloro-2-methyl-2*H*-indazol-5-yl)imino)-2,4-dioxo-3-((2-oxo-1,2-dihydropyridin-3-yl)methyl)-1,3,5-triazinan-1-yl)methyl)-5-fluoro-phenoxy)thiophene-2-carbonitrile *(25)***

A mixture of **25d** (95 mg，0.14 mmol), sodiumiodide (41.9 mg, 0.28 mmol), and chlorotrimethylsilane (30.4 mg，0.280 mmol) in Acetonitrile (5 mL) was stirred at 65°C for 1 h. The reaction mixture was diluted with water and EtOAc, and the aqueous layer was extracted with EtOAc. The organic layer was washed with water and brine, dried over Na_2_SO_4_, and concentrated under reduced pressure. The residue was purified by silica gel column chromatography (DCM: MeOH = 20:1) and preparative HPLC to afford **25** (60 mg, 64%) as pale-yellow solid. MS (ESI) m/z: 665.1[M+H]^+^. ^1^H NMR (400 MHz, CD_3_OD) *δ* 8.15 (s, 1H), 7.72-7.67 (m, 2H), 7.44 (d, *J* = 4.2 Hz, 1H), 7.38-7.29 (m, 3H), 7.14 (d, *J* = 9.6 Hz, 1H), 6.61 (d, *J* = 4.2 Hz, 1H), 6.34 (t, *J* = 6.7 Hz, 1H), 5.32 (s, 2H), 4.86 (s, 2H), 4.17 (s, 3H).

## 5. Spectral data: NMR and MS/HPLC traces for compounds 2-25

^1^H NMR spectrum of compound **2**


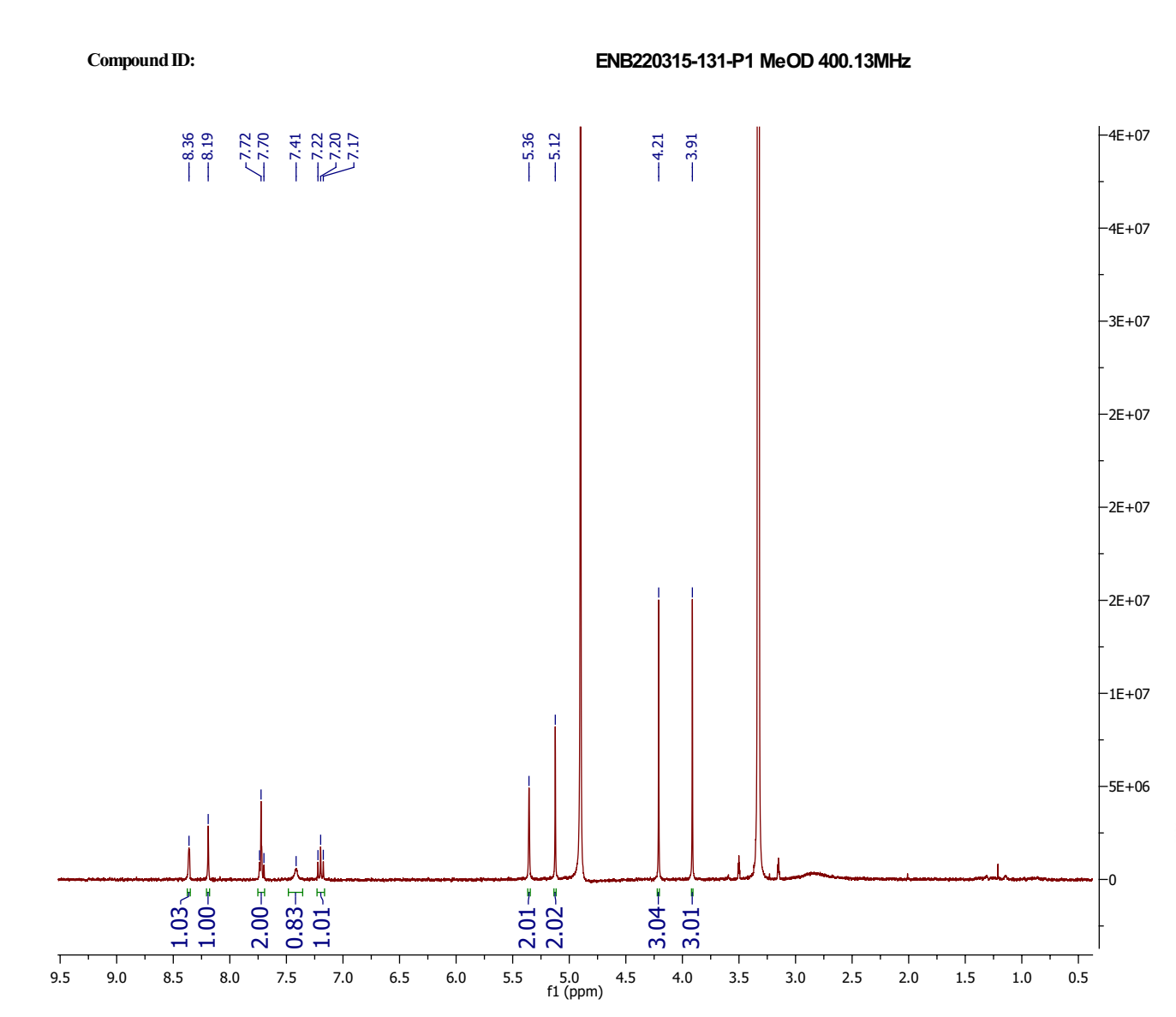


HPLC chart of compound **2**


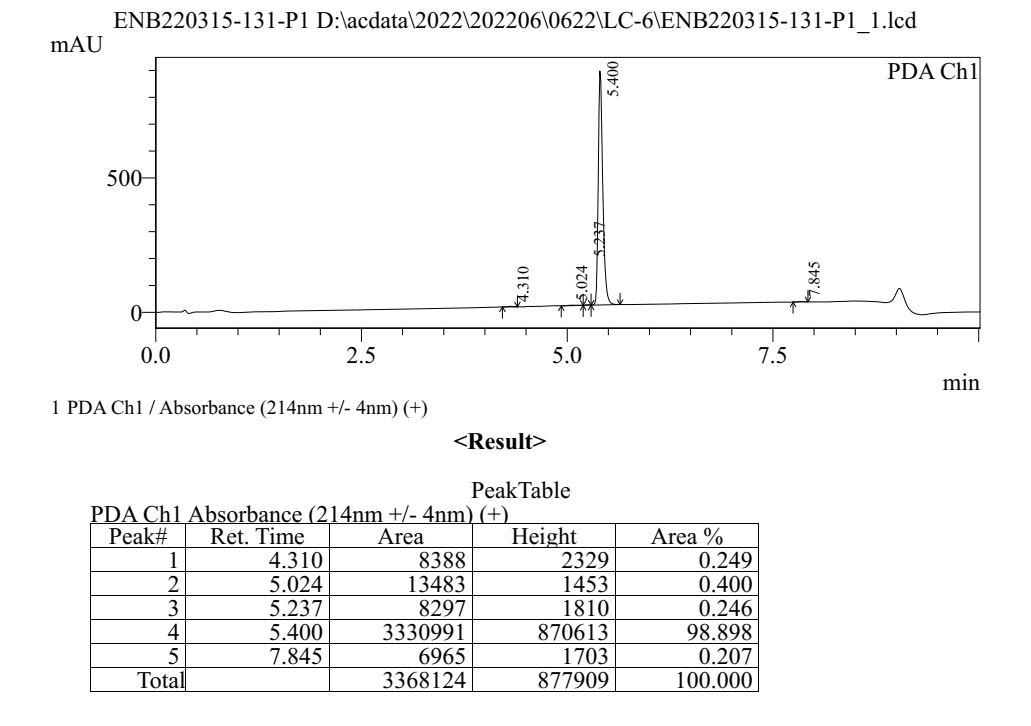


^1^H NMR spectrum of compound **3**


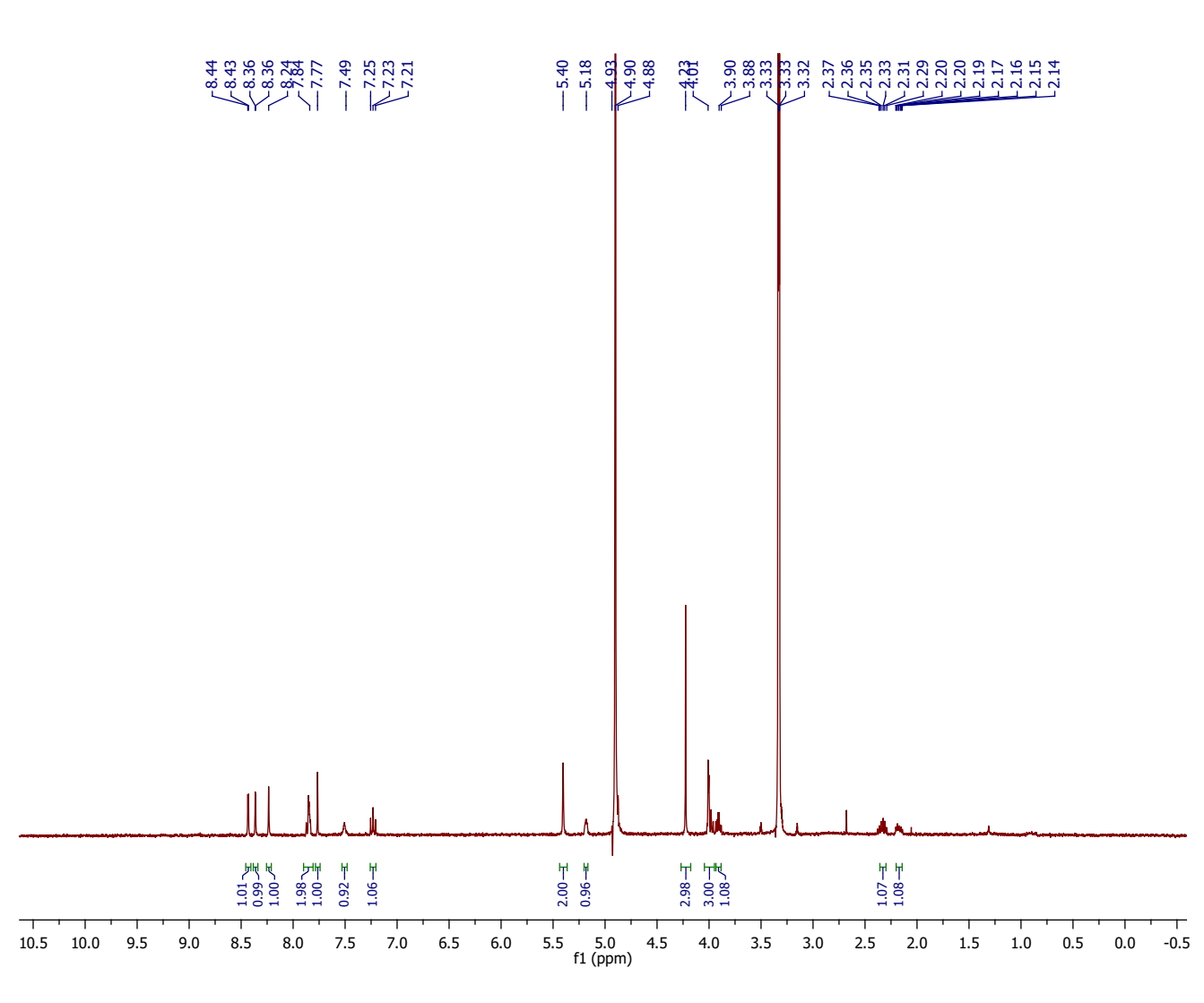


HPLC chart of compound **3**


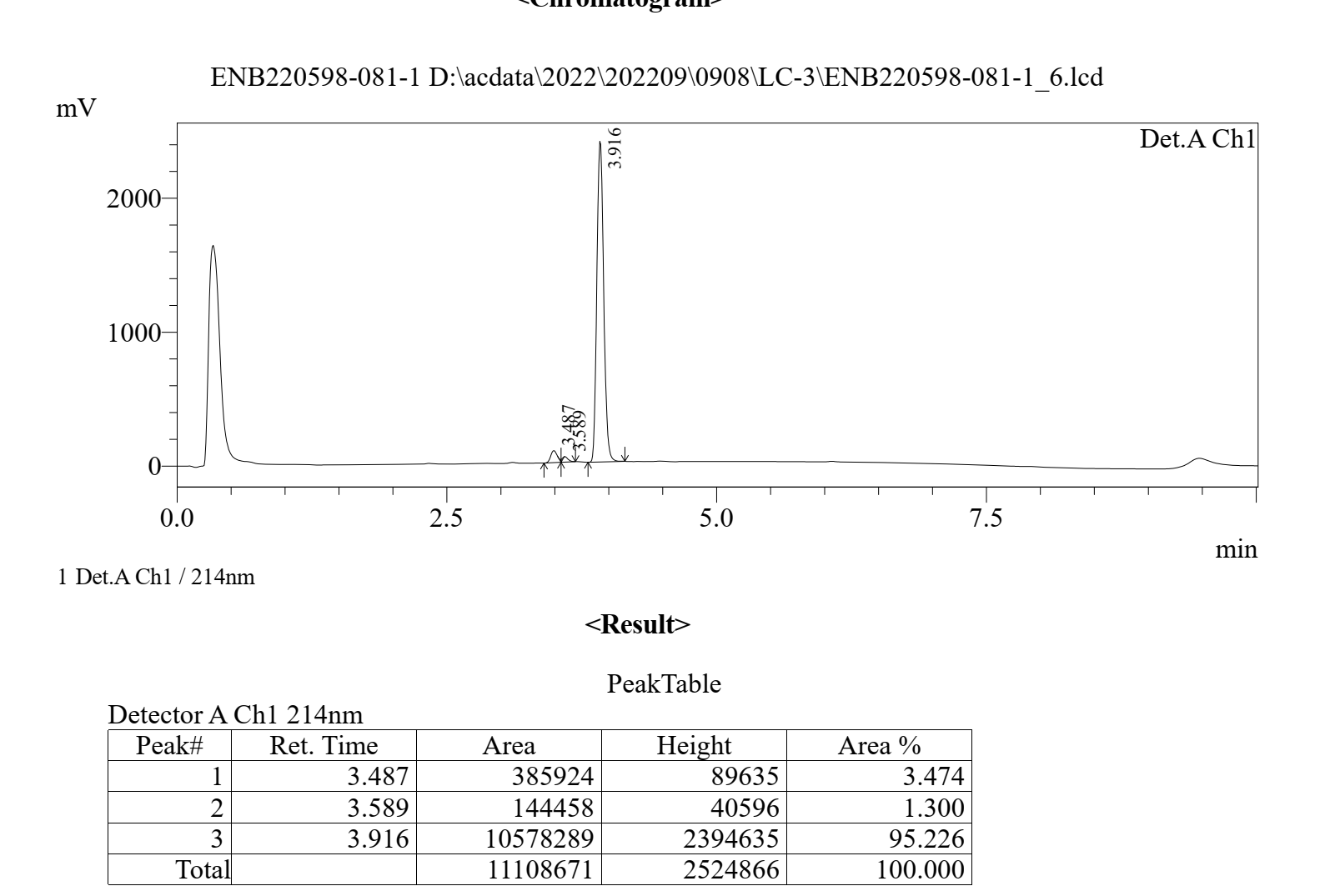


^1^H NMR spectrum of compound **4**

HPLC chart of compound **4**


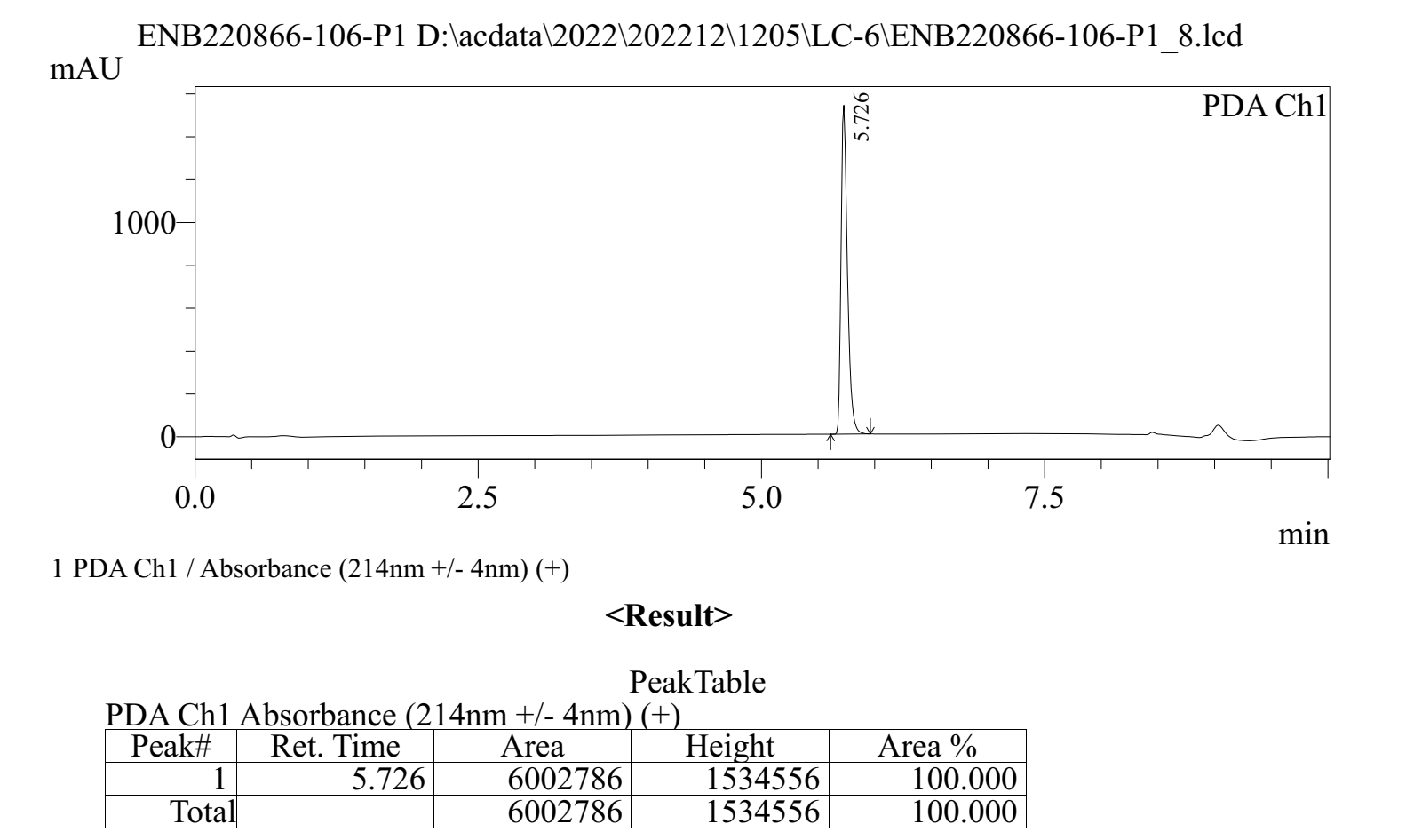


^1^H NMR spectrum of compound **5**


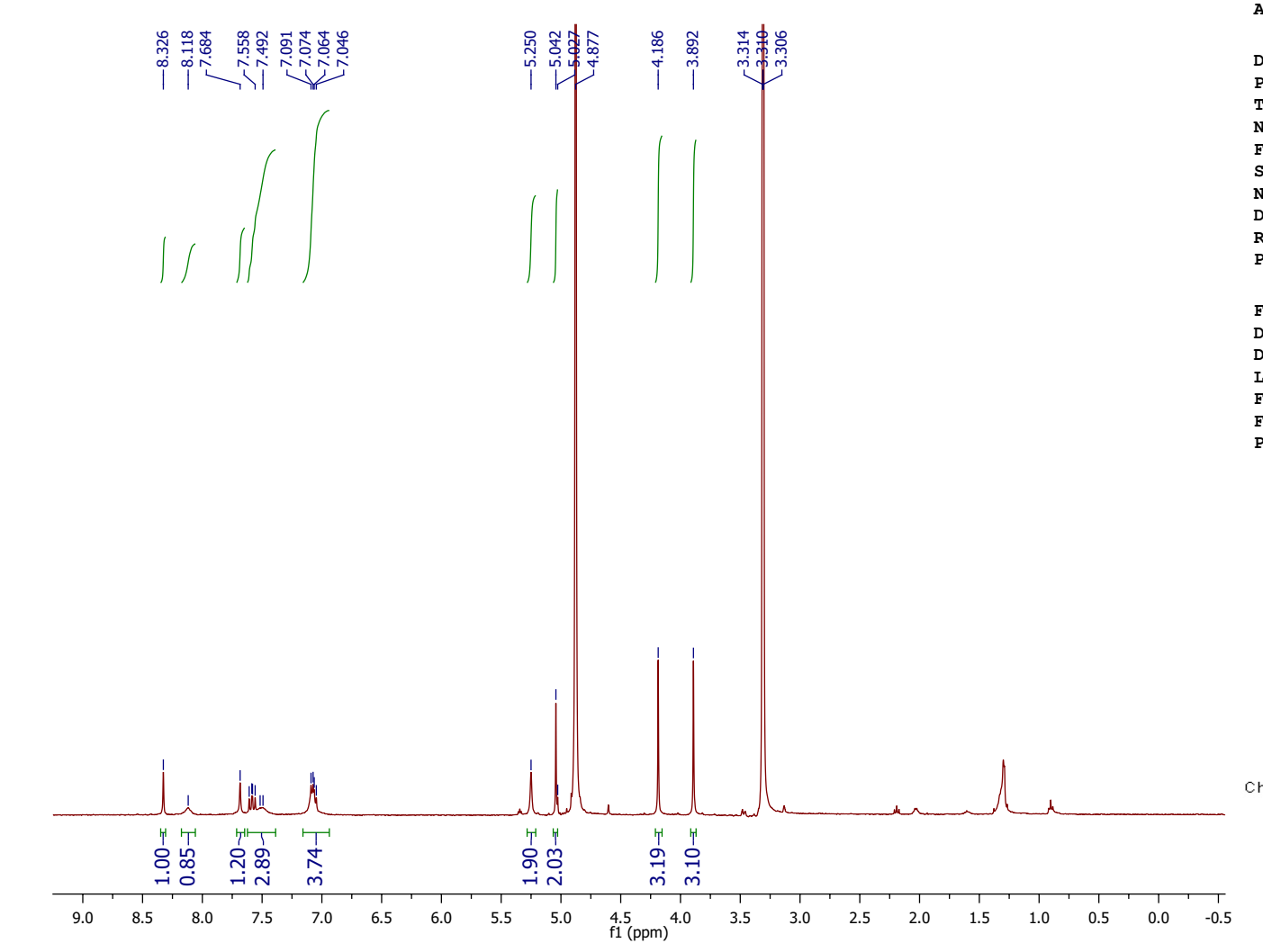


HPLC chart of compound **5**


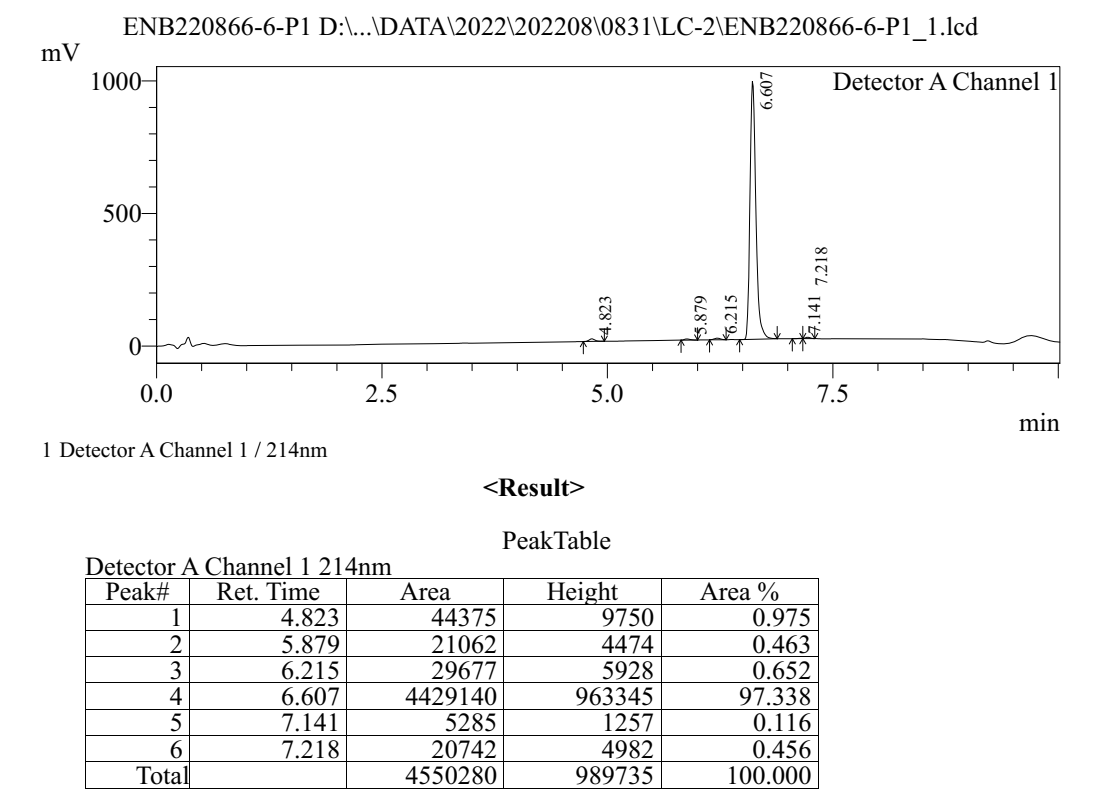


^1^H NMR spectrum of compound **6**


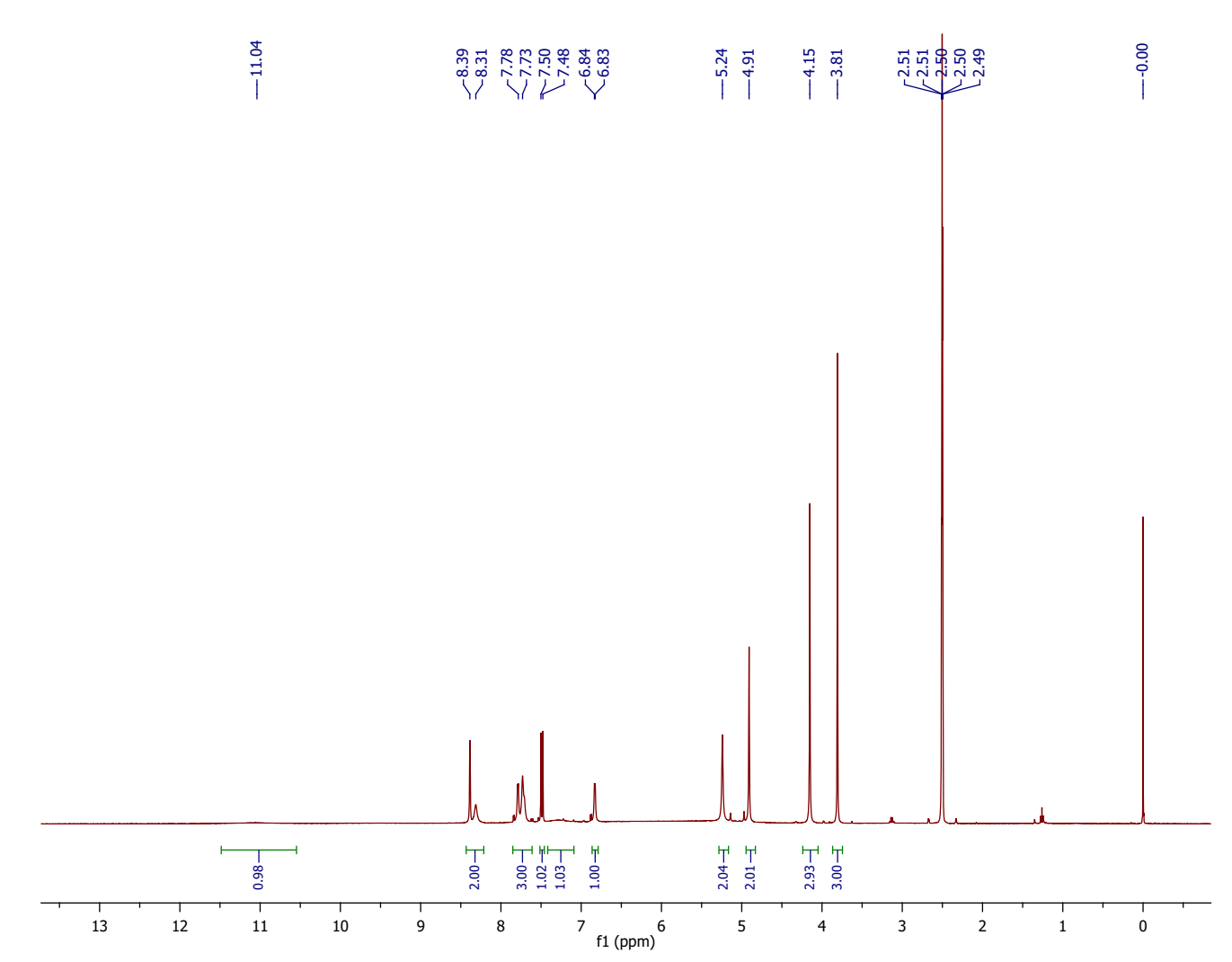


HPLC chart of compound **6**


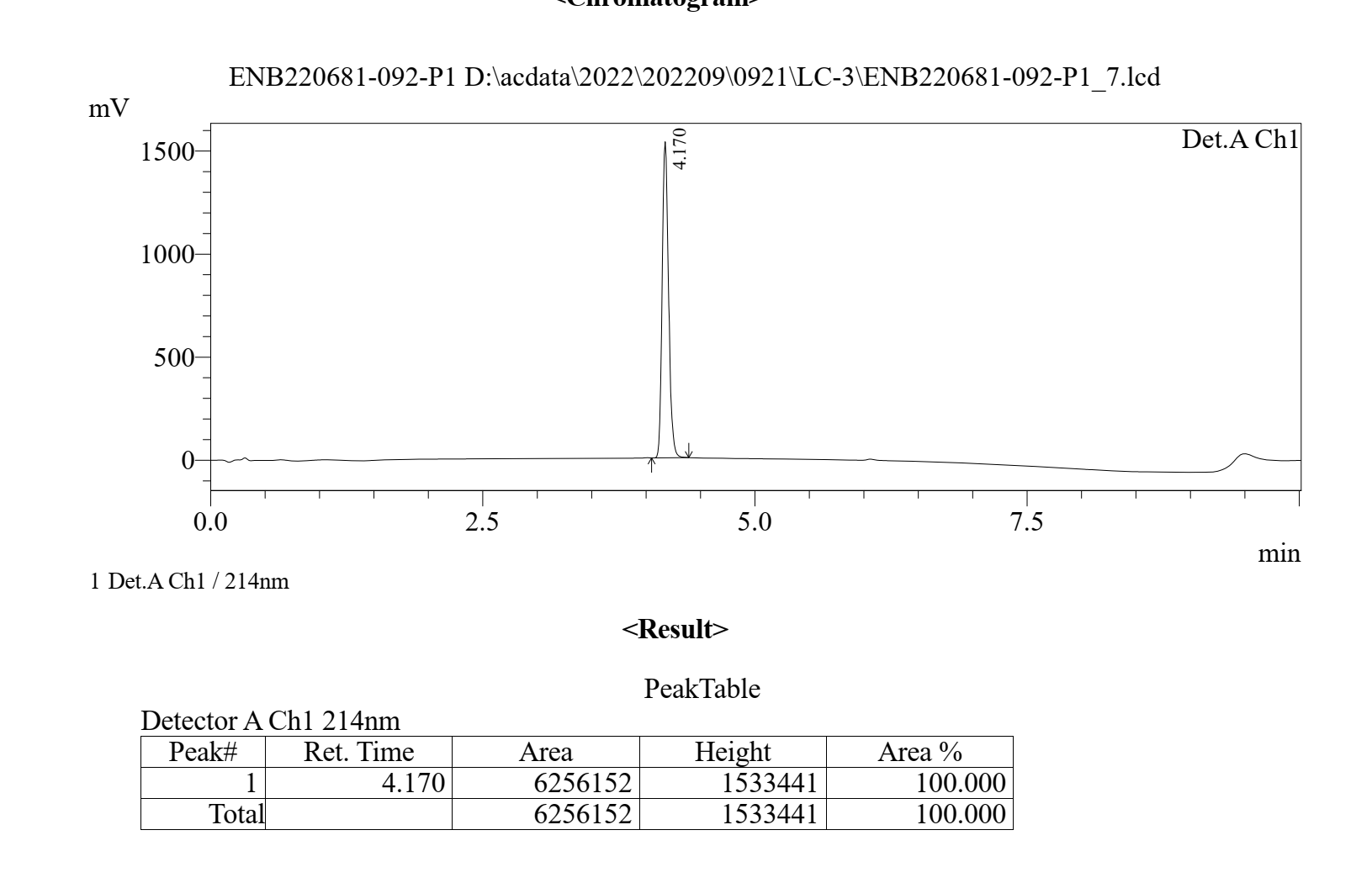


^1^H NMR spectrum of compound **7**


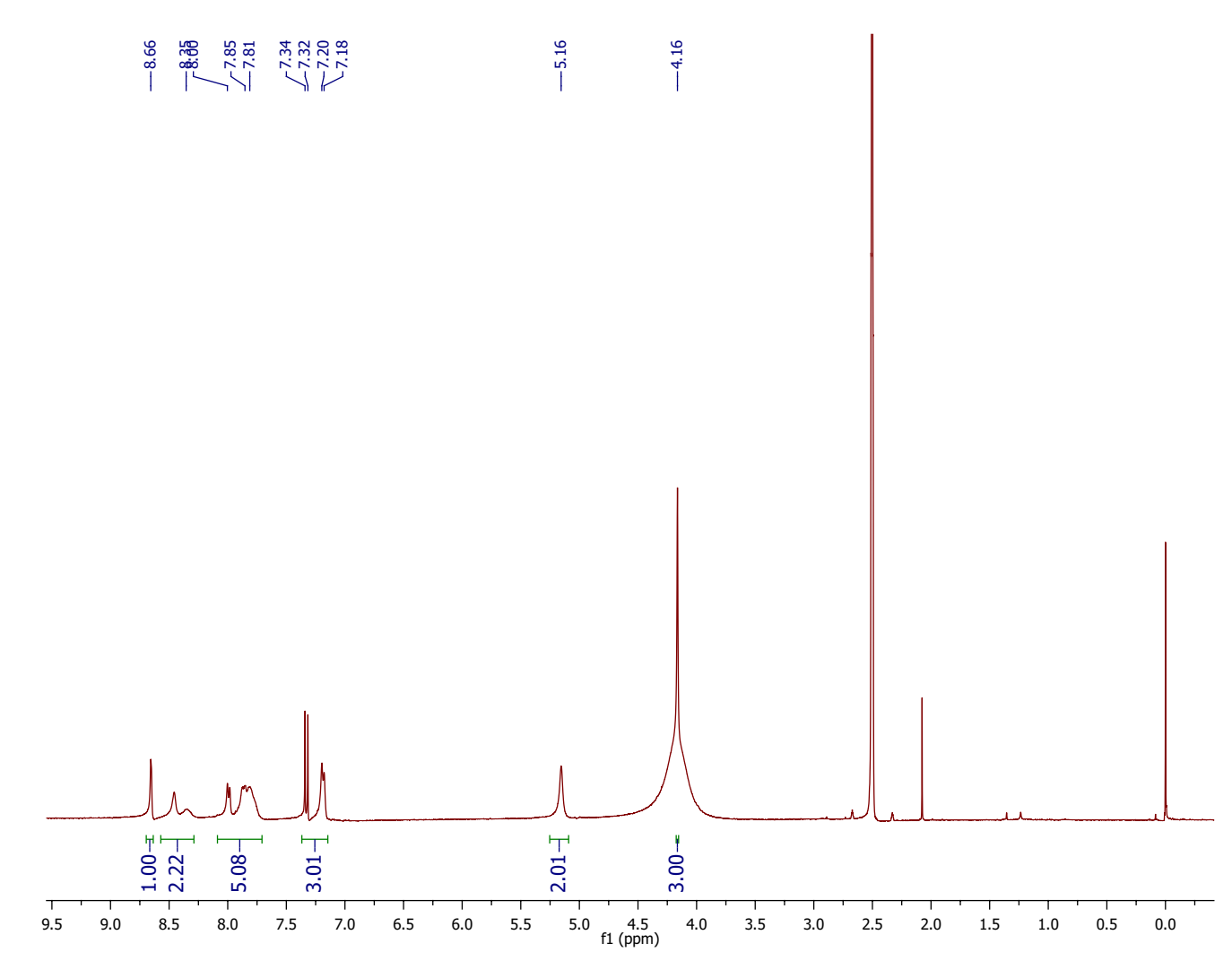


HPLC chart of compound **7**


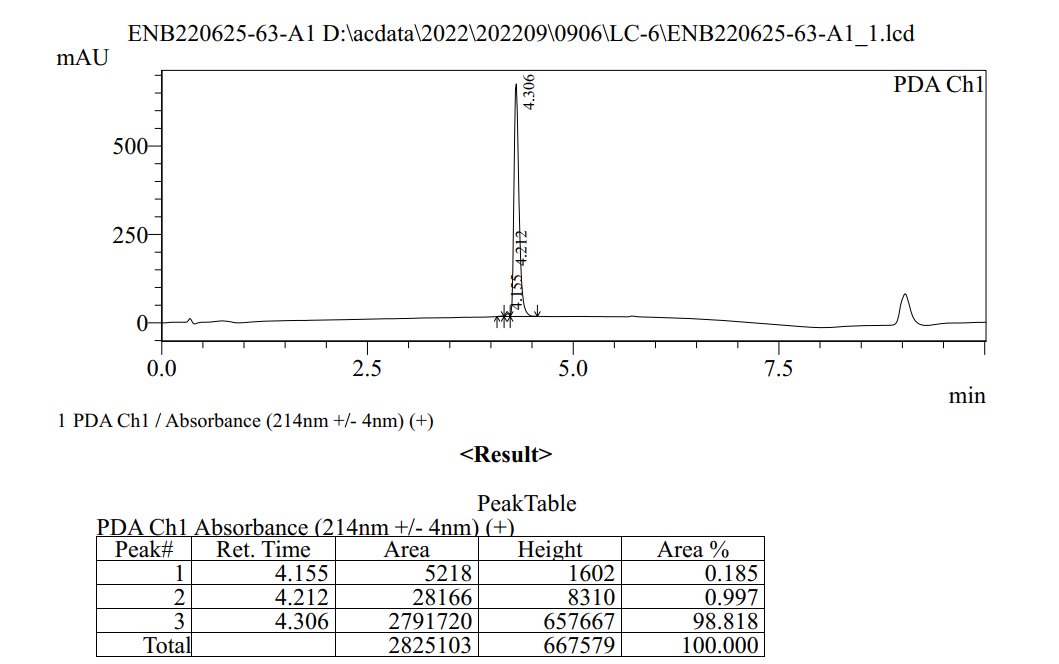


^1^H NMR spectrum of compound **8**


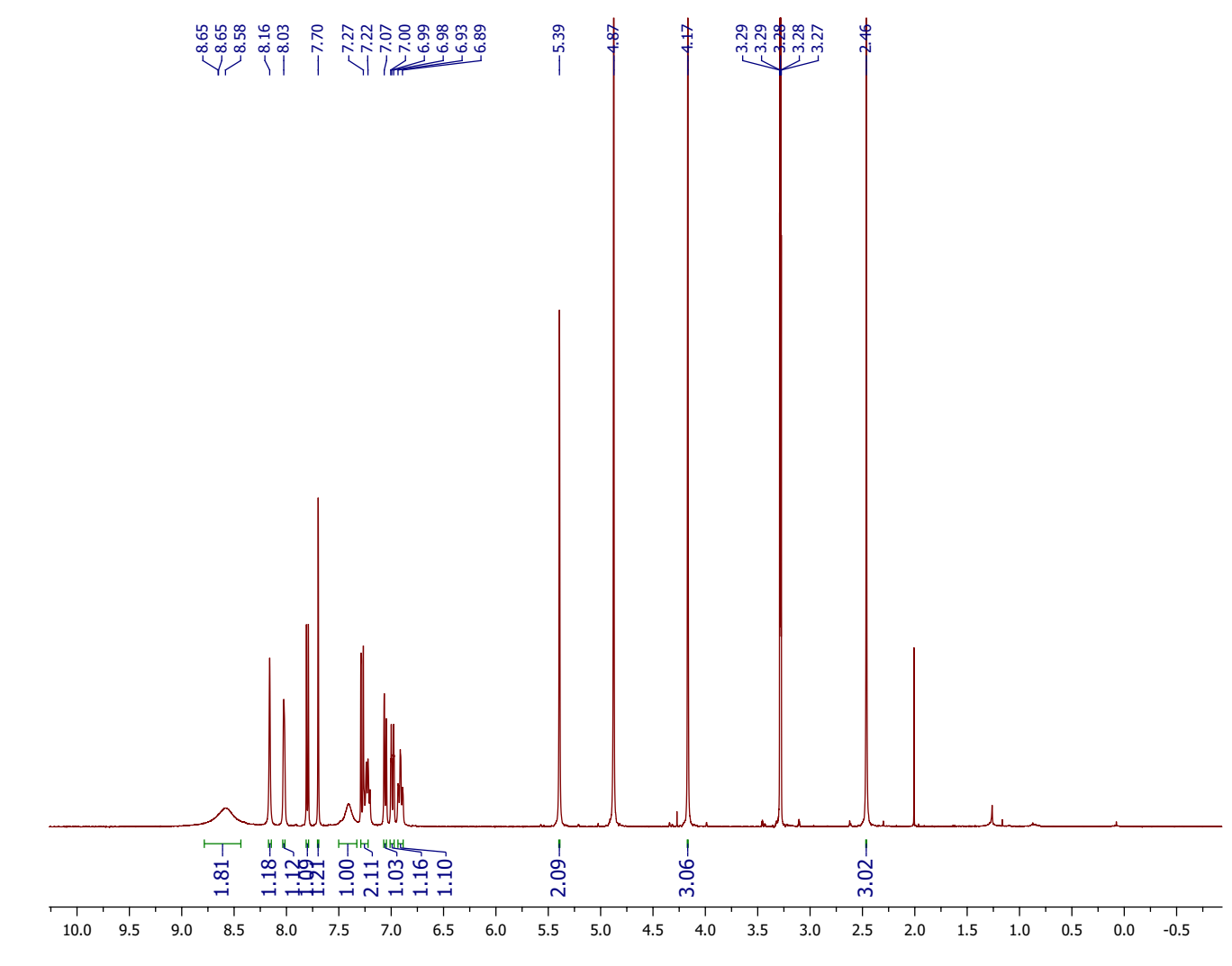


HPLC chart of compound **8**


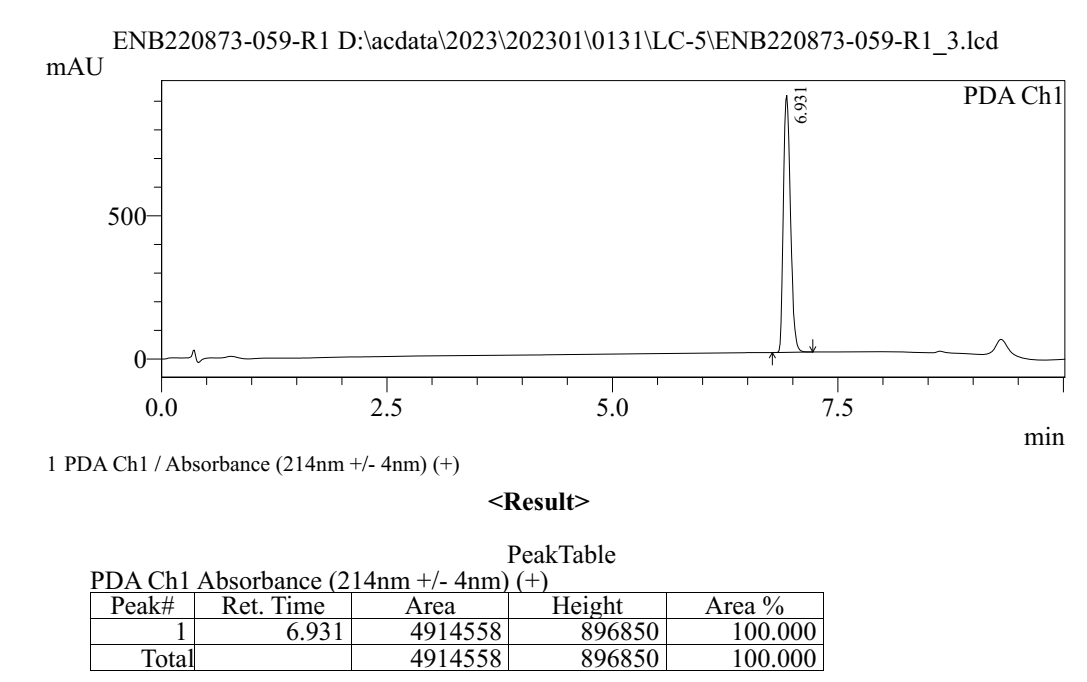


MS chart of compound **8**


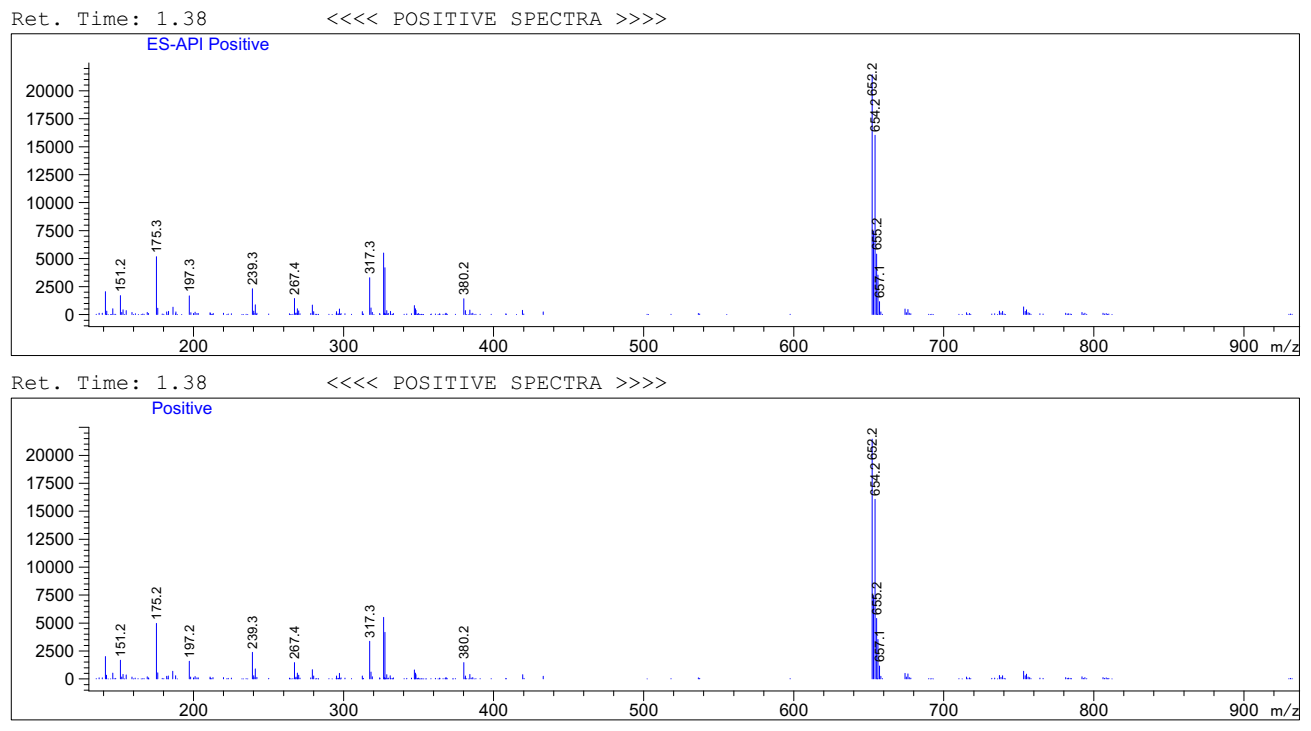


^1^H NMR spectrum of compound **9**


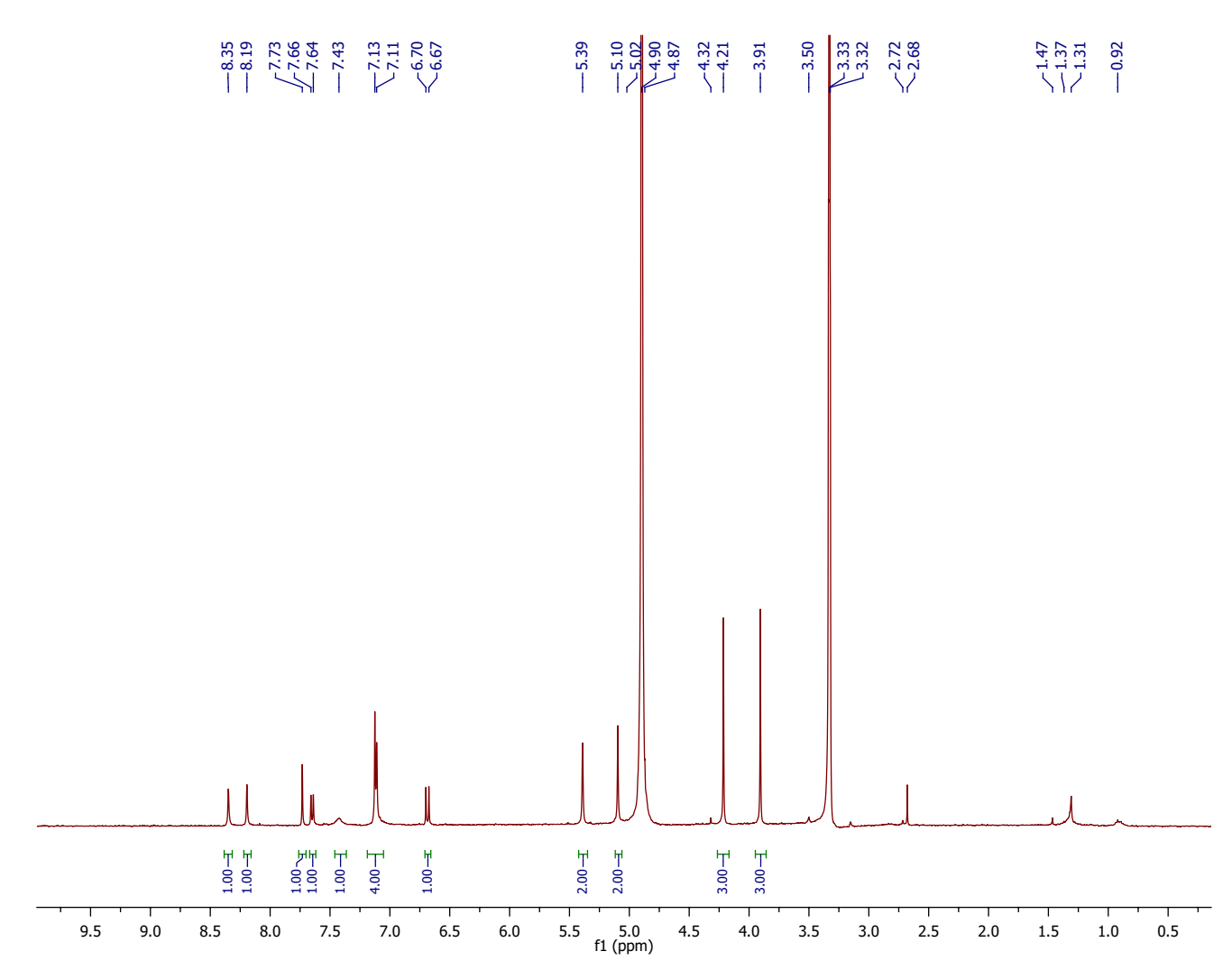


HPLC chart of compound **9**


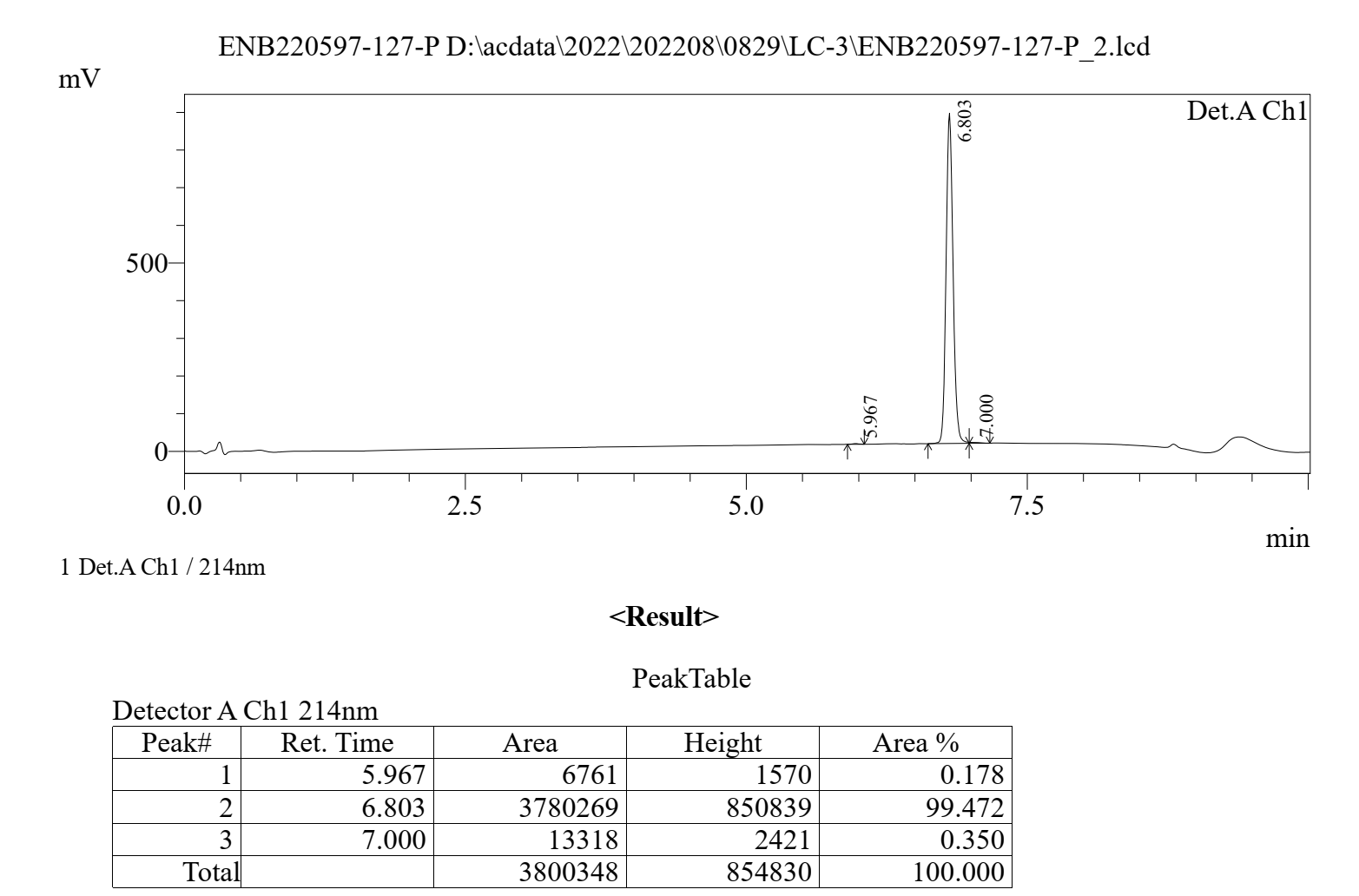


MS chart of compound **9**


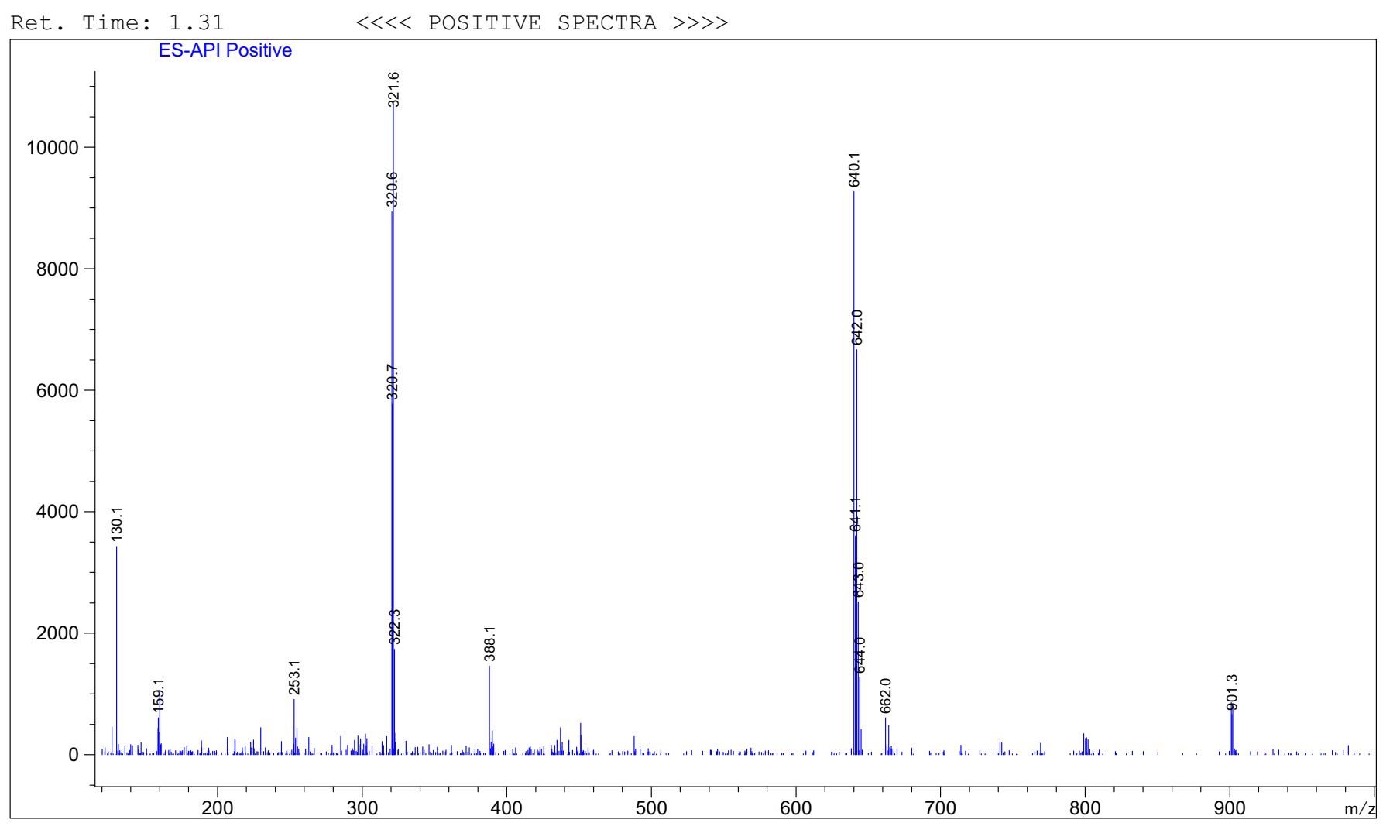


^1^H NMR spectrum of compound **10**


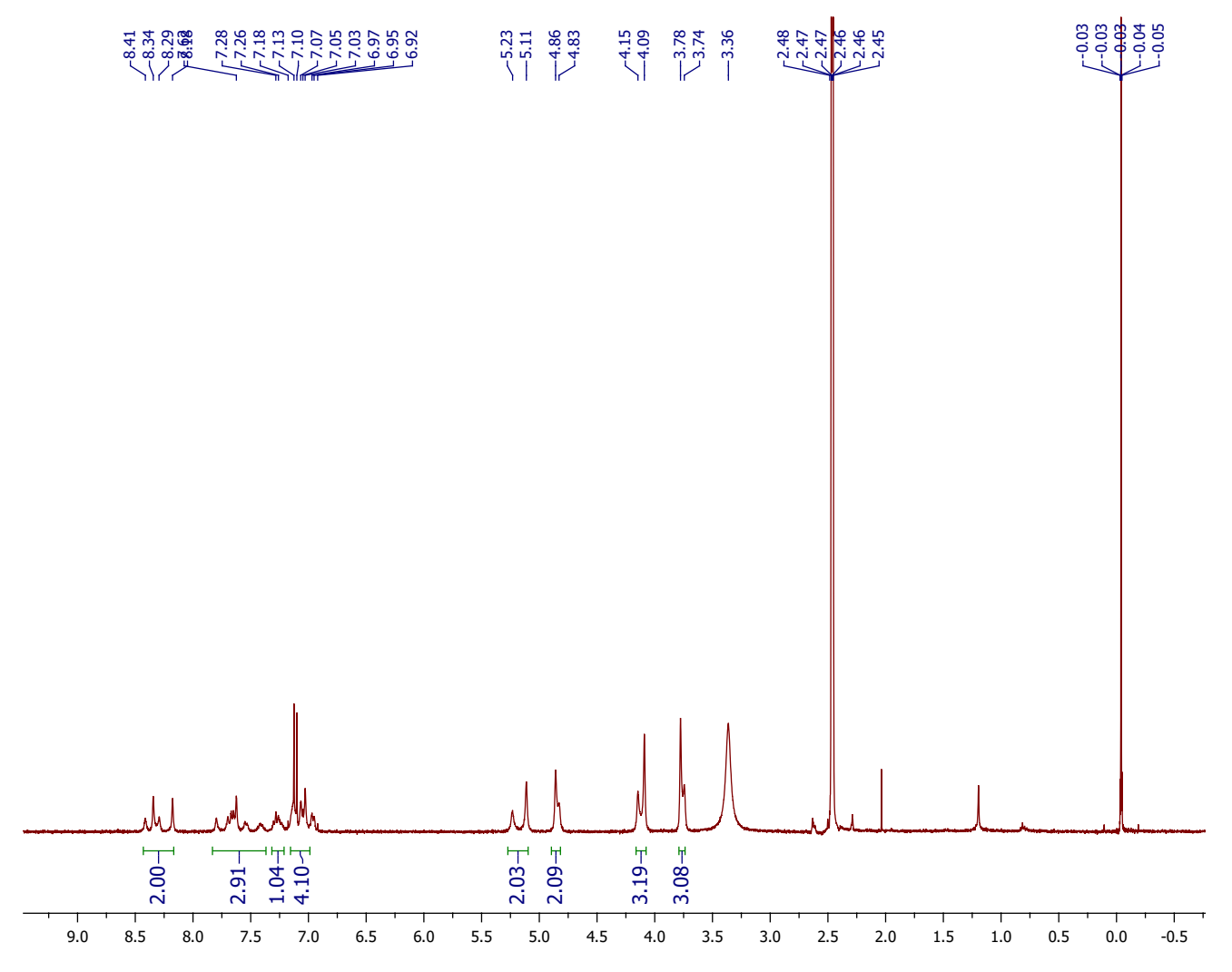


HPLC chart of compound **10**


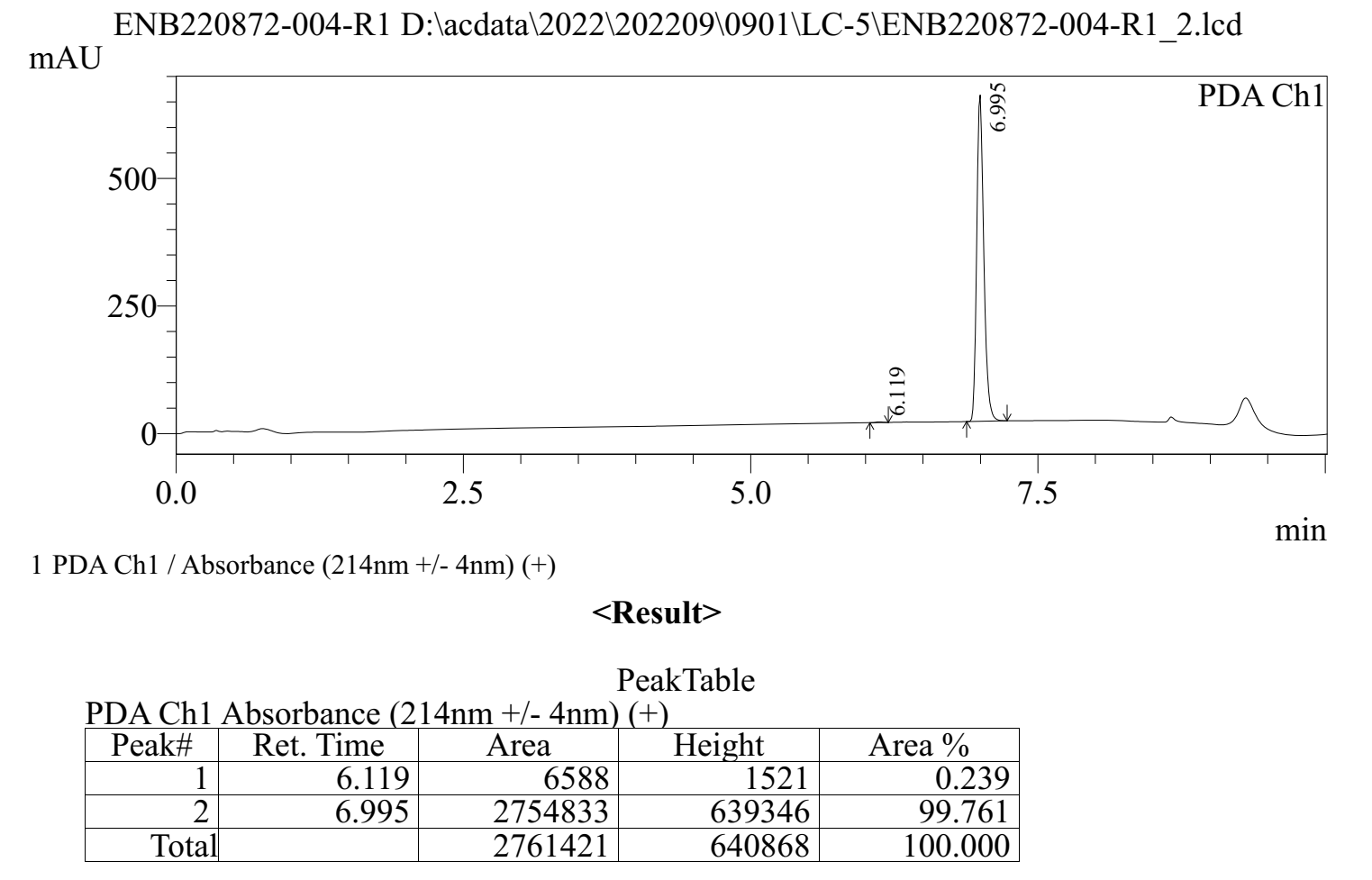


MS chart of compound **10**


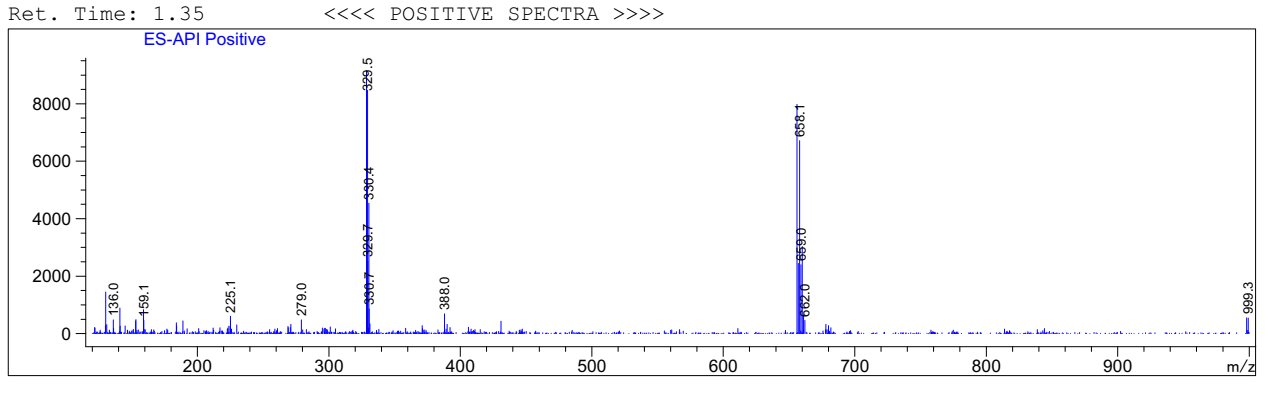


^1^H NMR spectrum of compound **11**


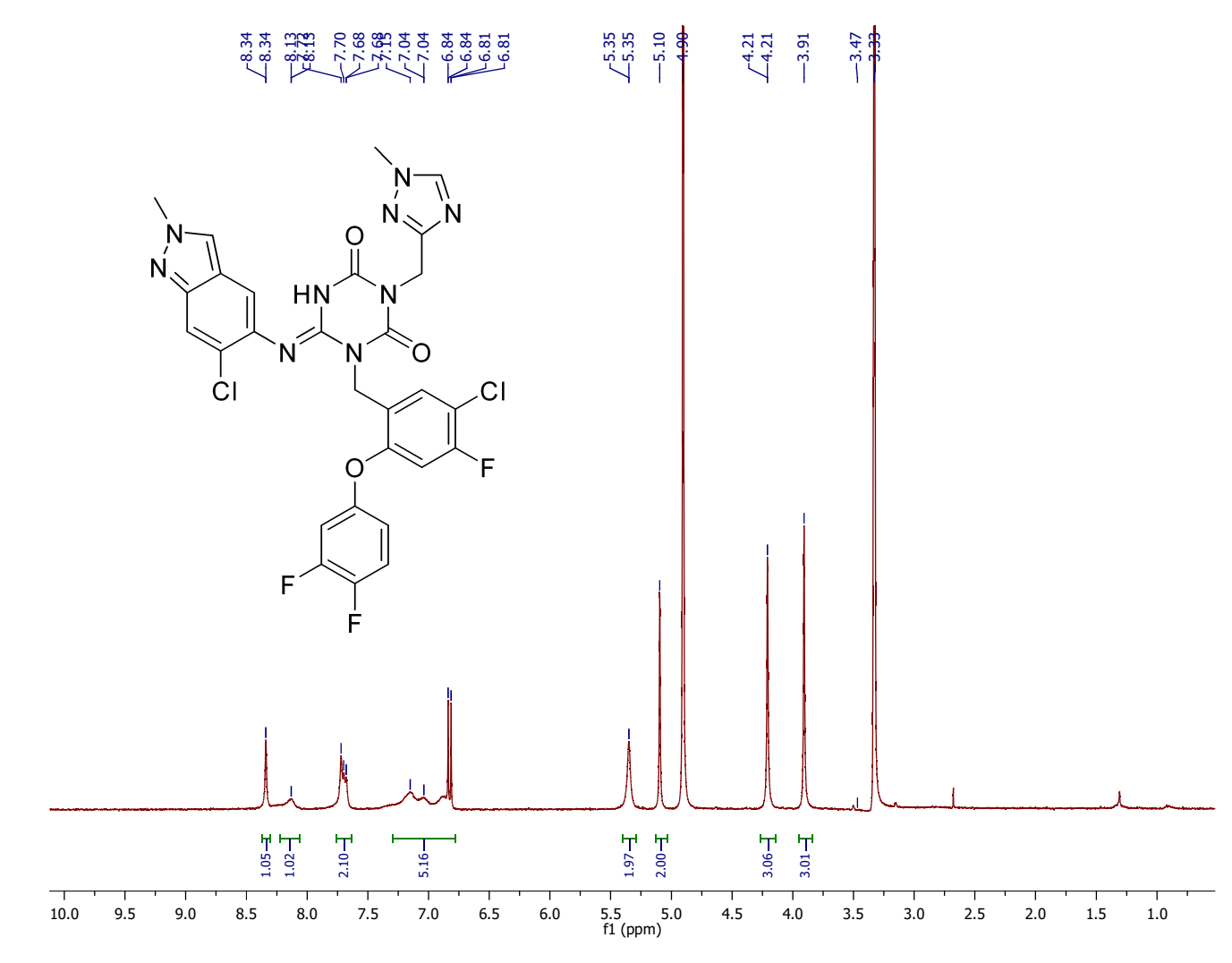


HPLC chart of compound **11**


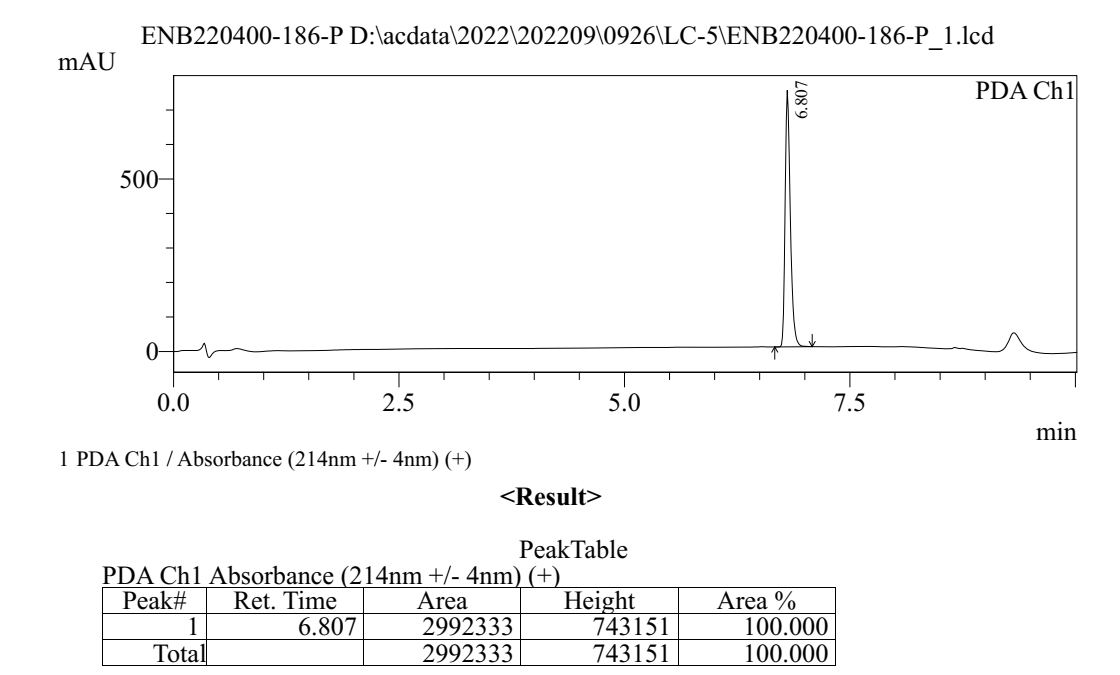


^1^H NMR spectrum of compound **12**


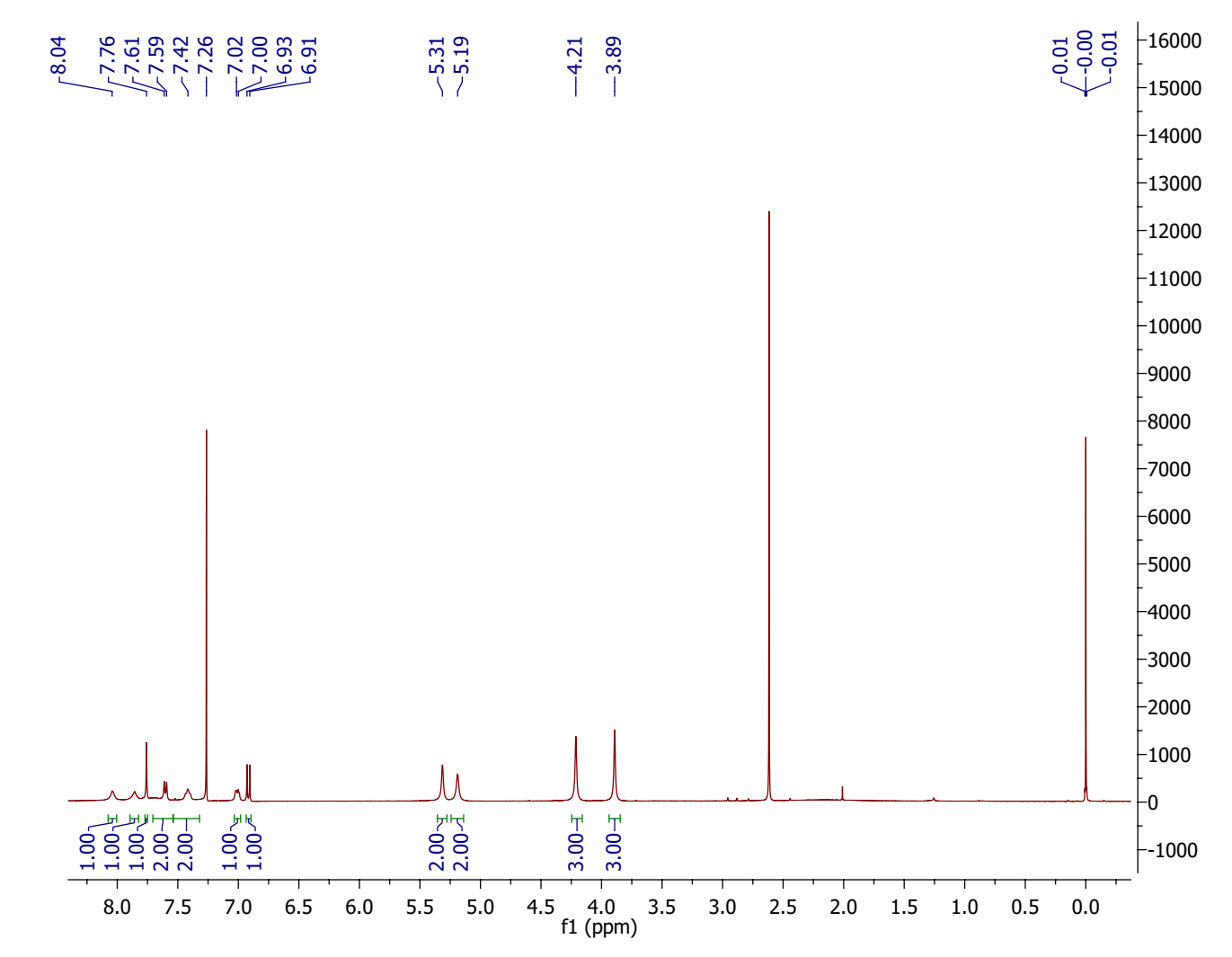


HPLC chart of compound **12**


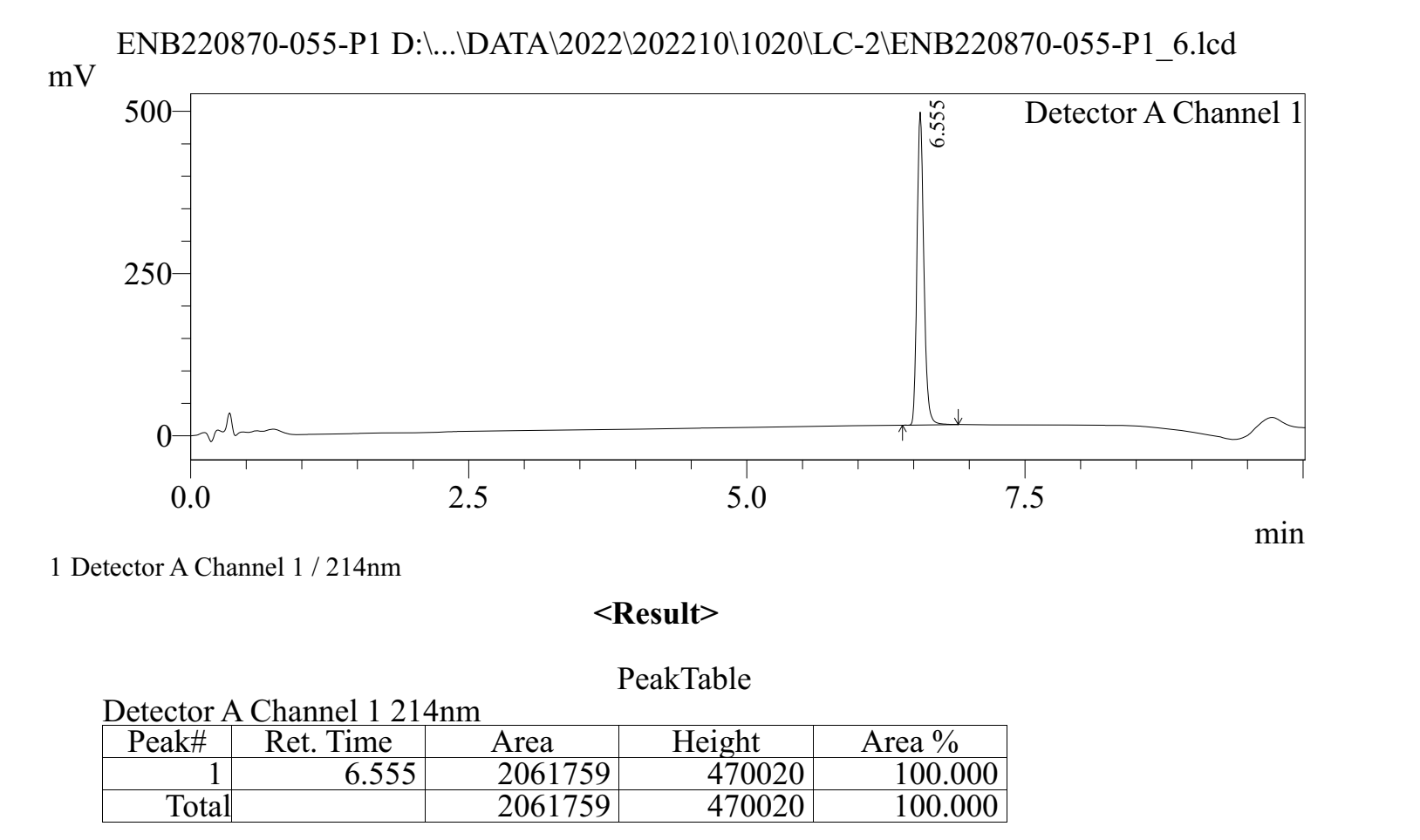


^1^H NMR spectrum of compound **13**


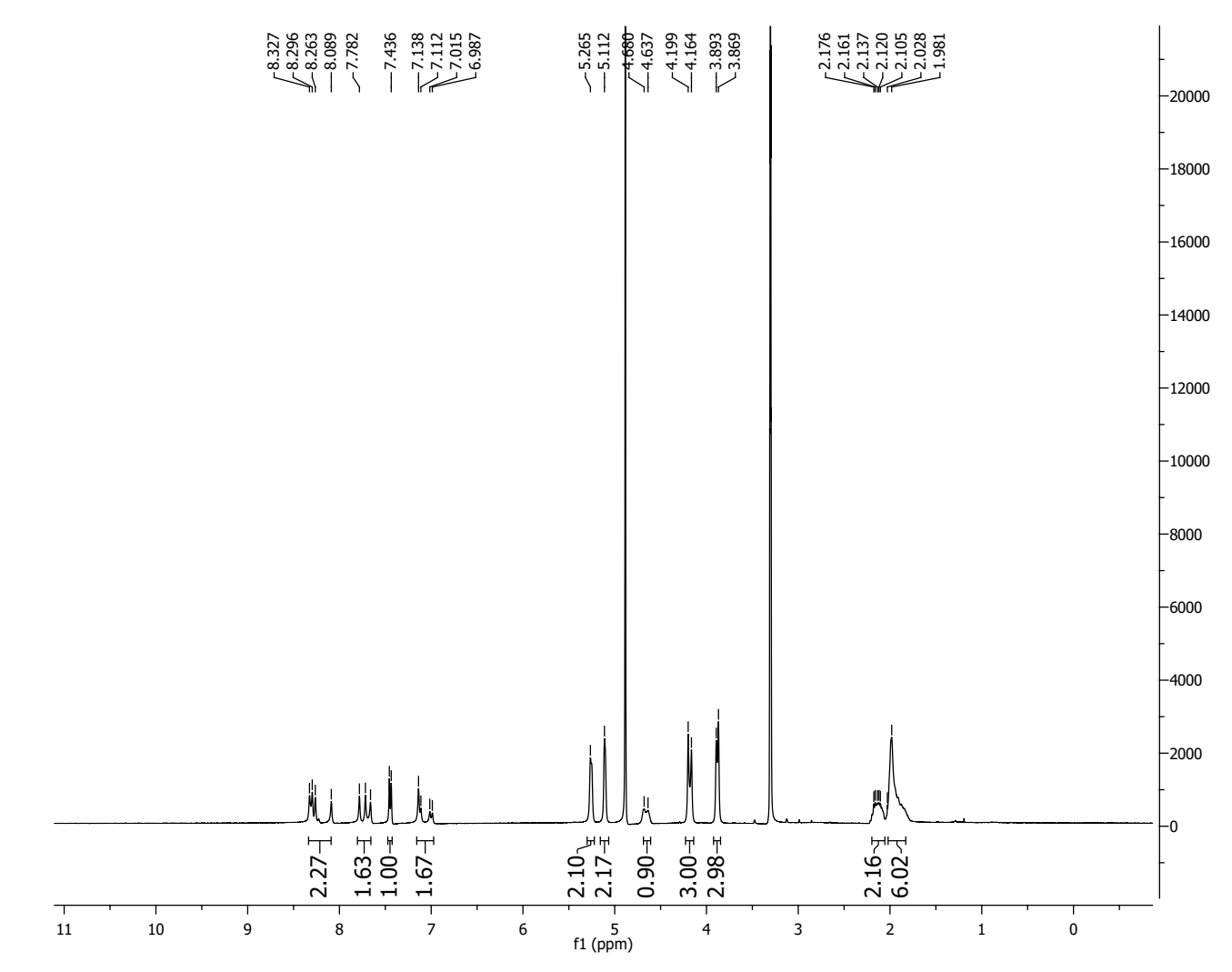


HPLC chart of compound **13**


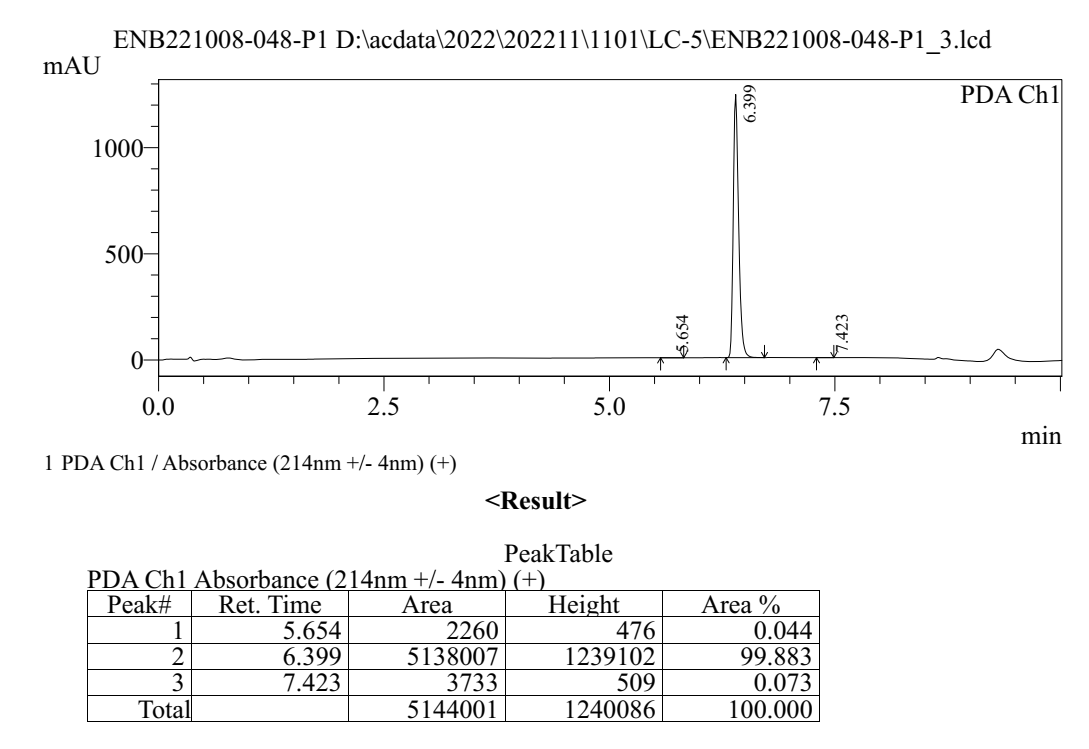


MS chart of compound **13**


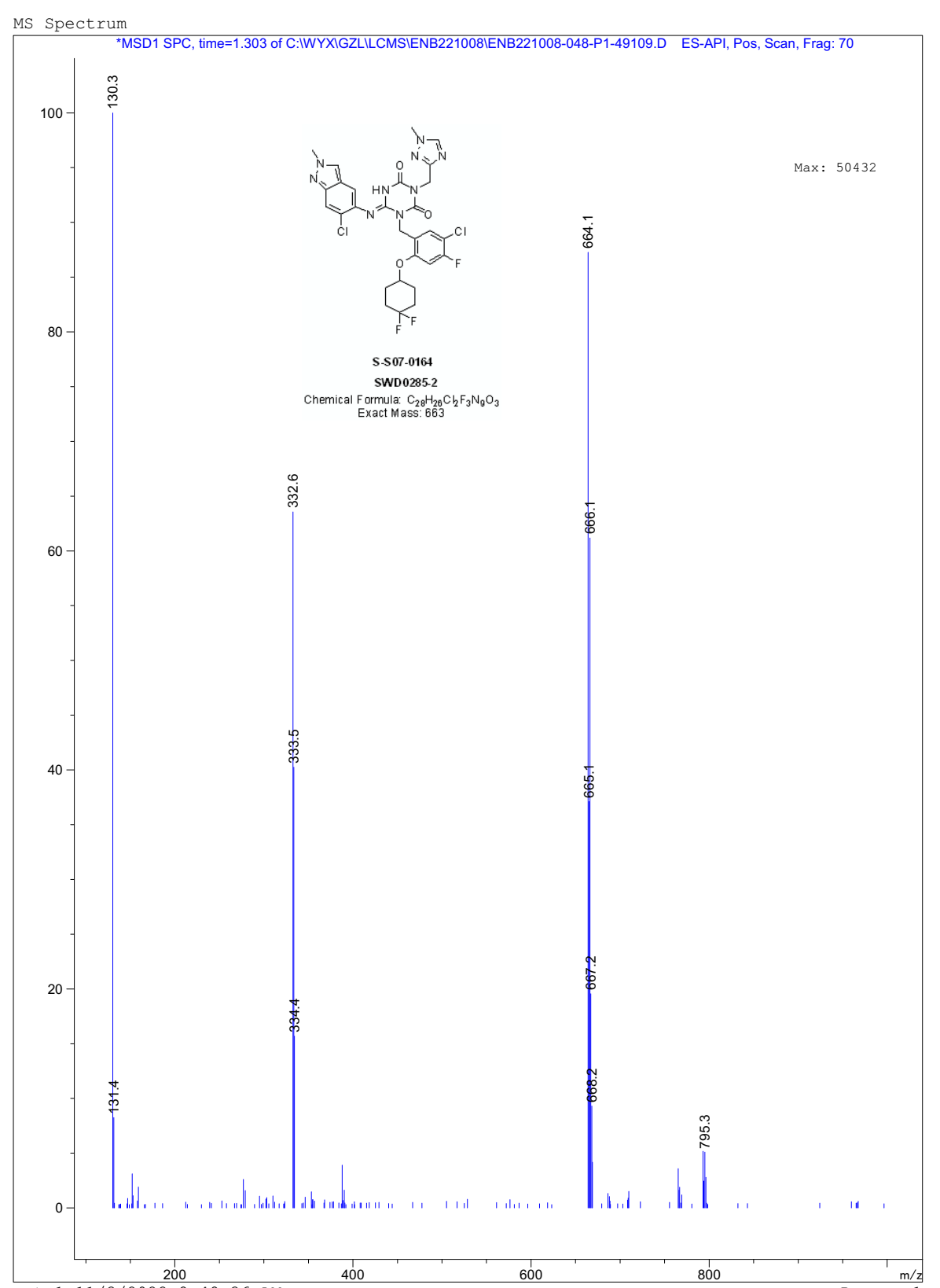


^1^H NMR spectrum of compound **14**


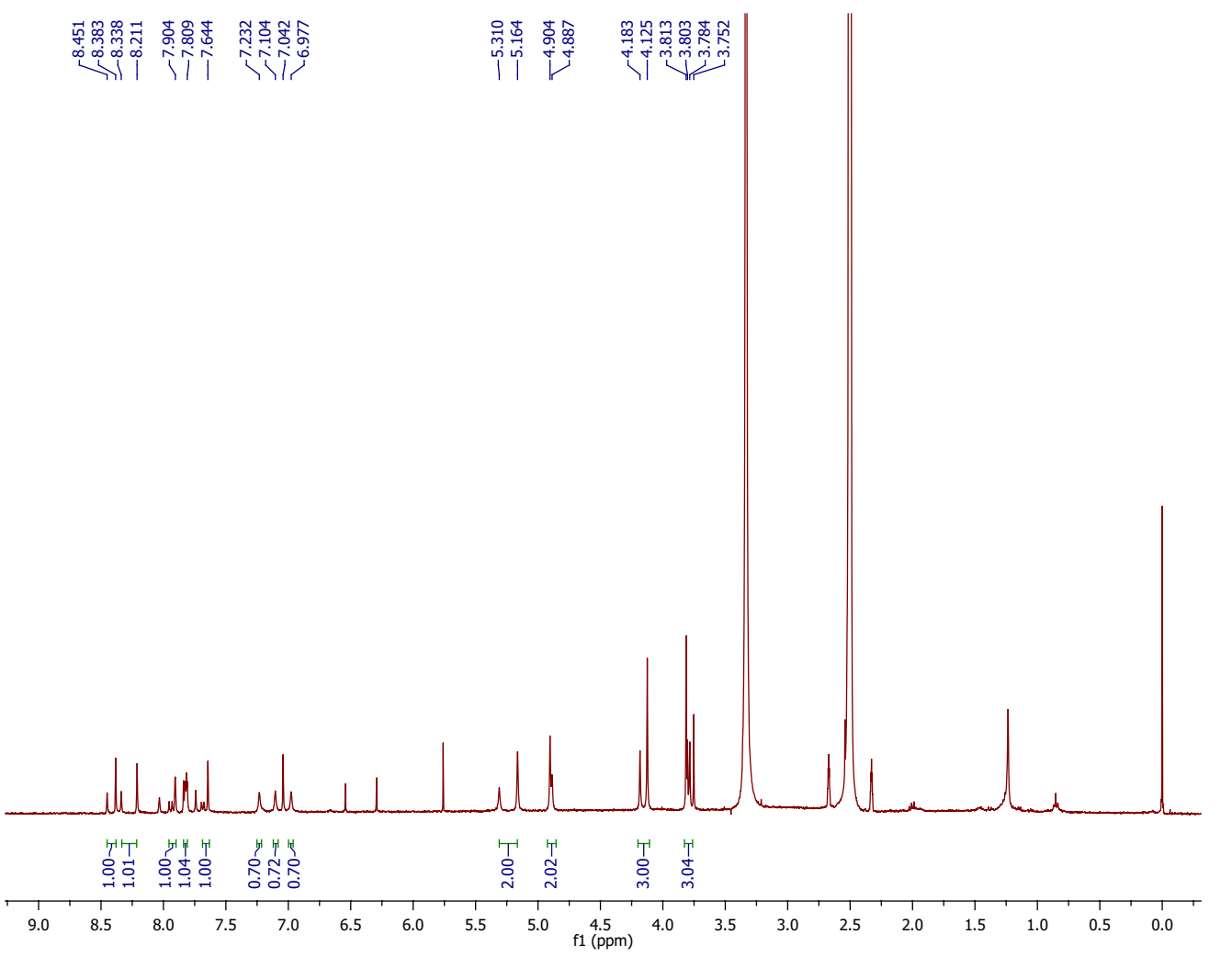


HPLC chart of compound **14**


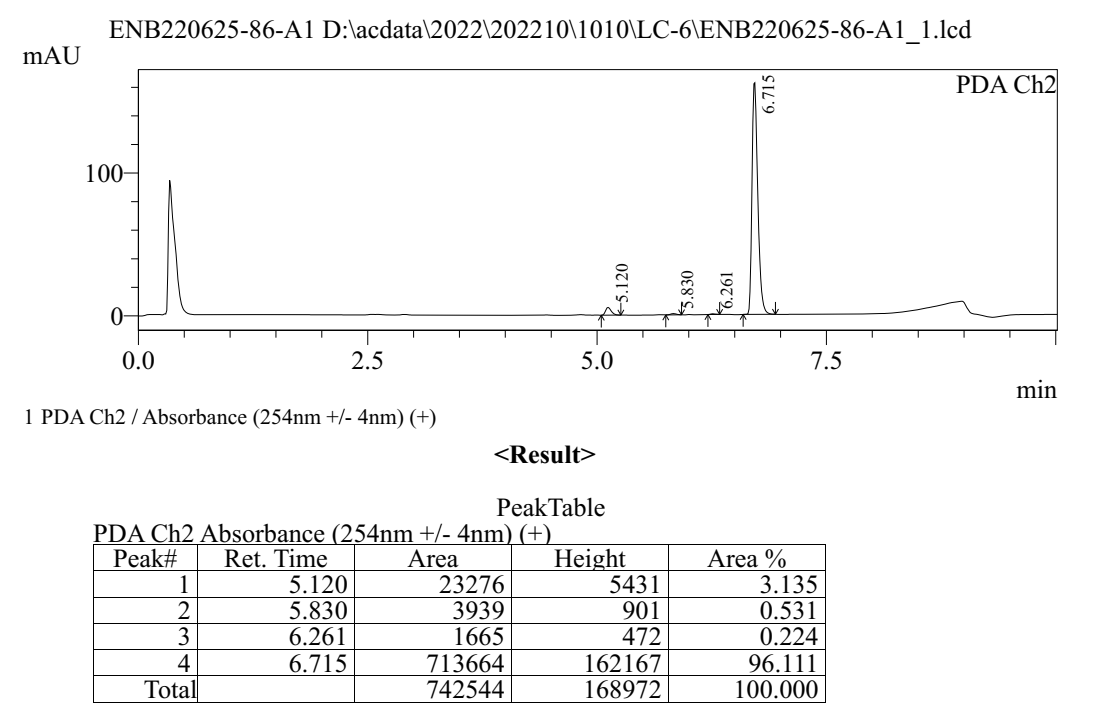


MS chart of compound **14**


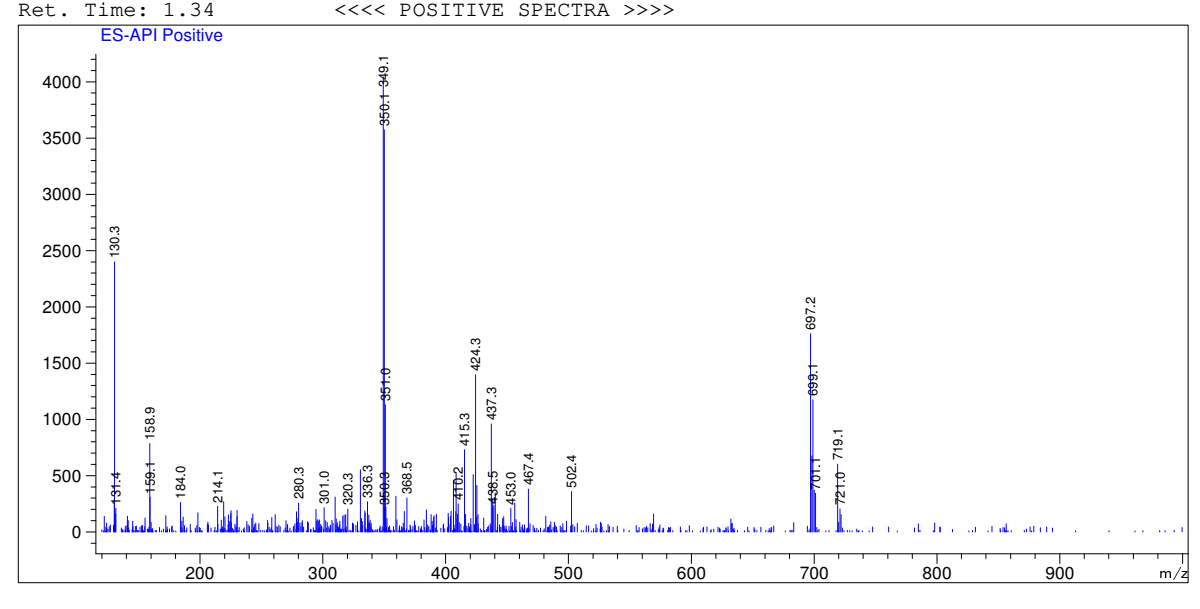


^1^H NMR spectrum of compound **15**


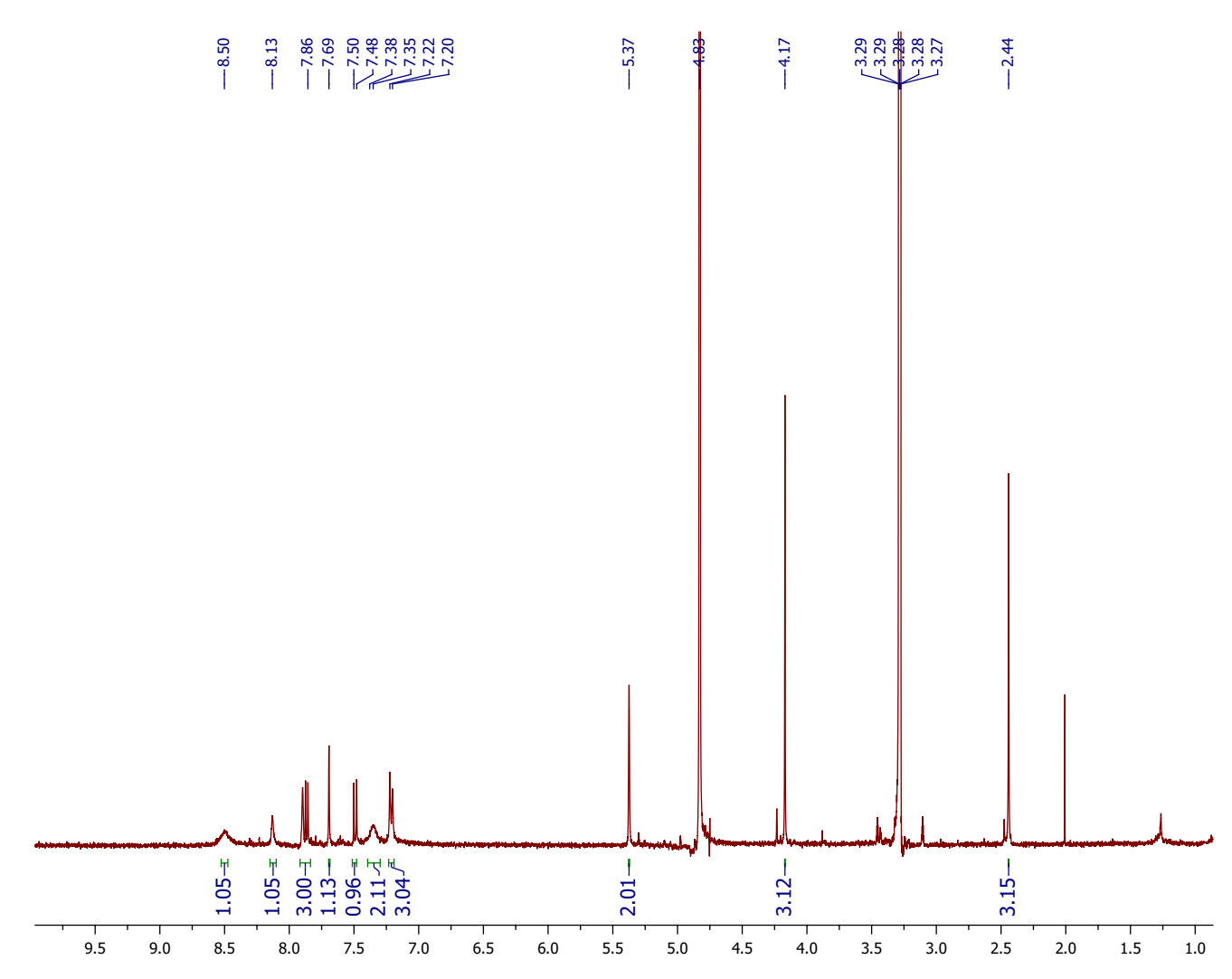


HPLC chart of compound **15**


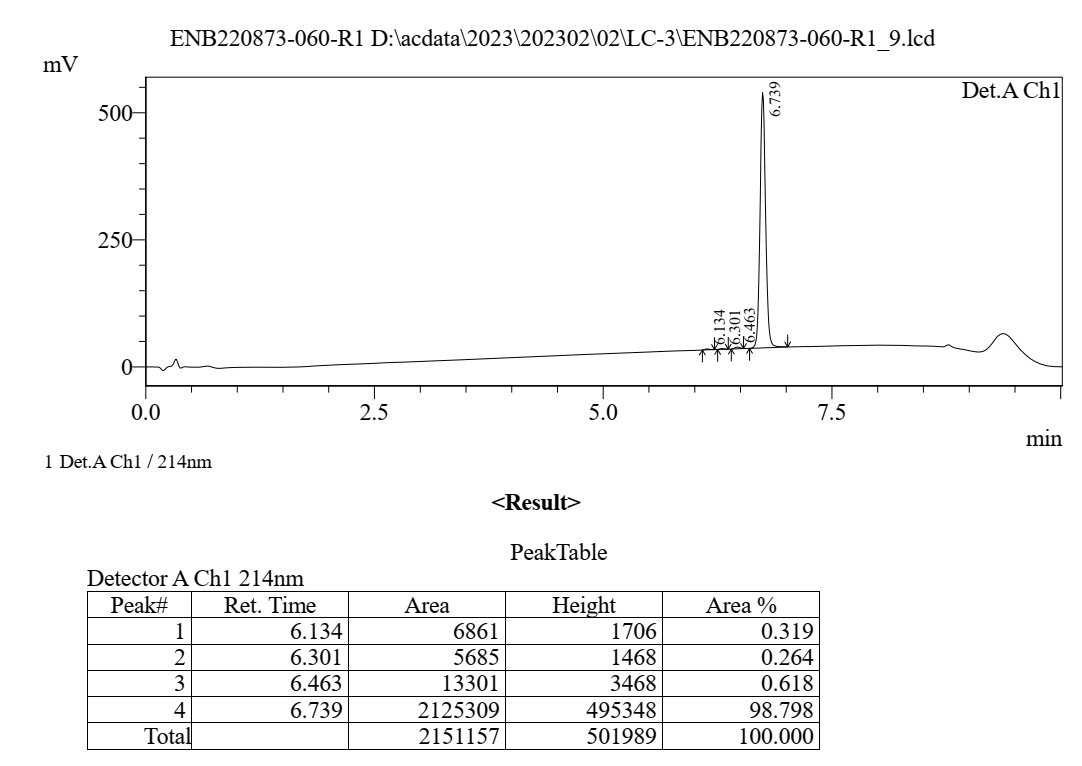


MS chart of compound **15**


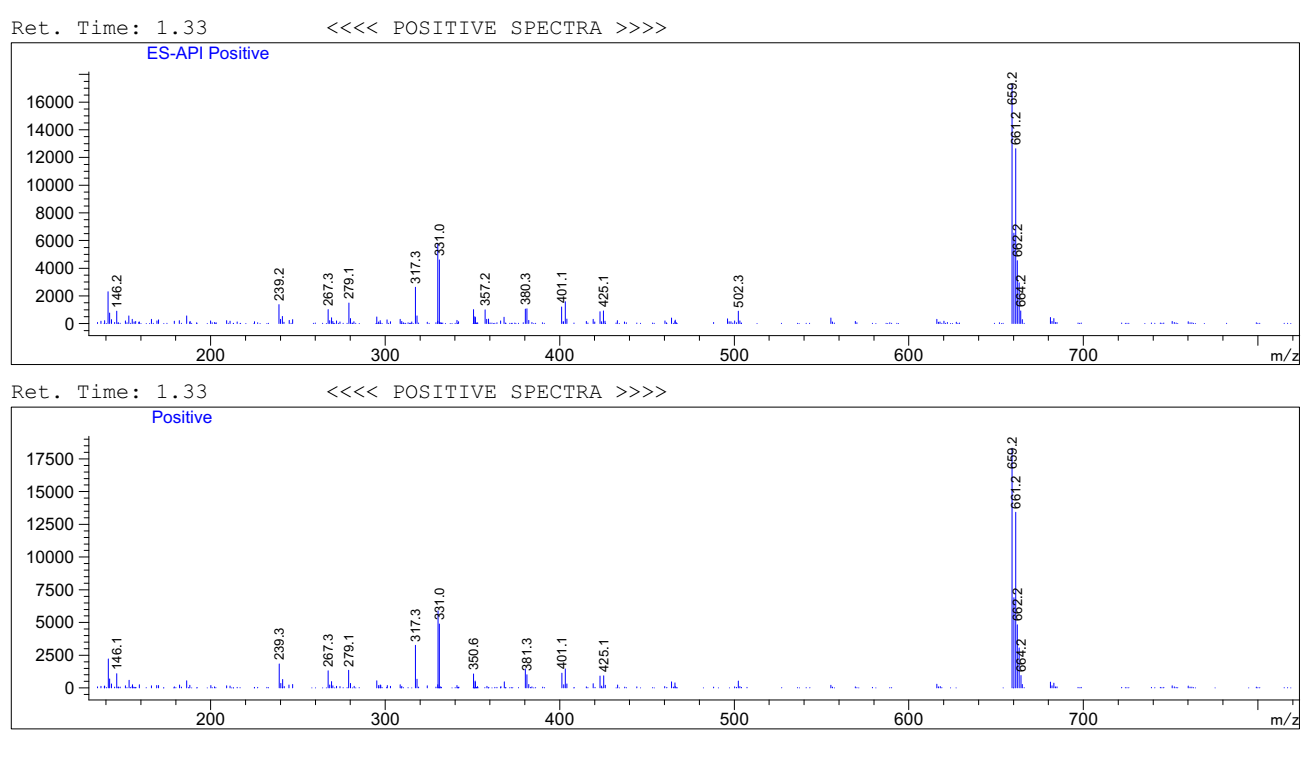


^1^H NMR spectrum of compound **16**


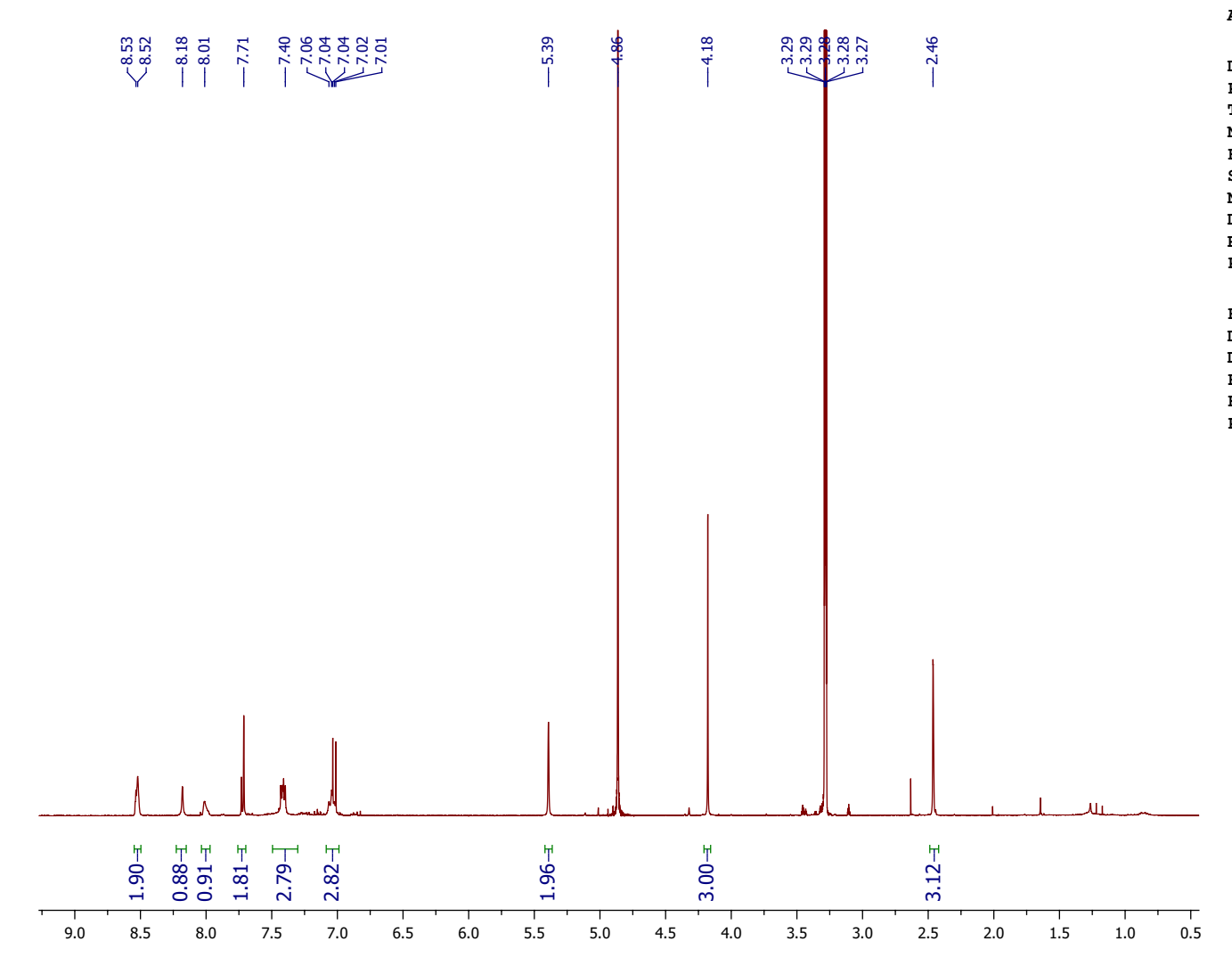


HPLC chart of compound **16**


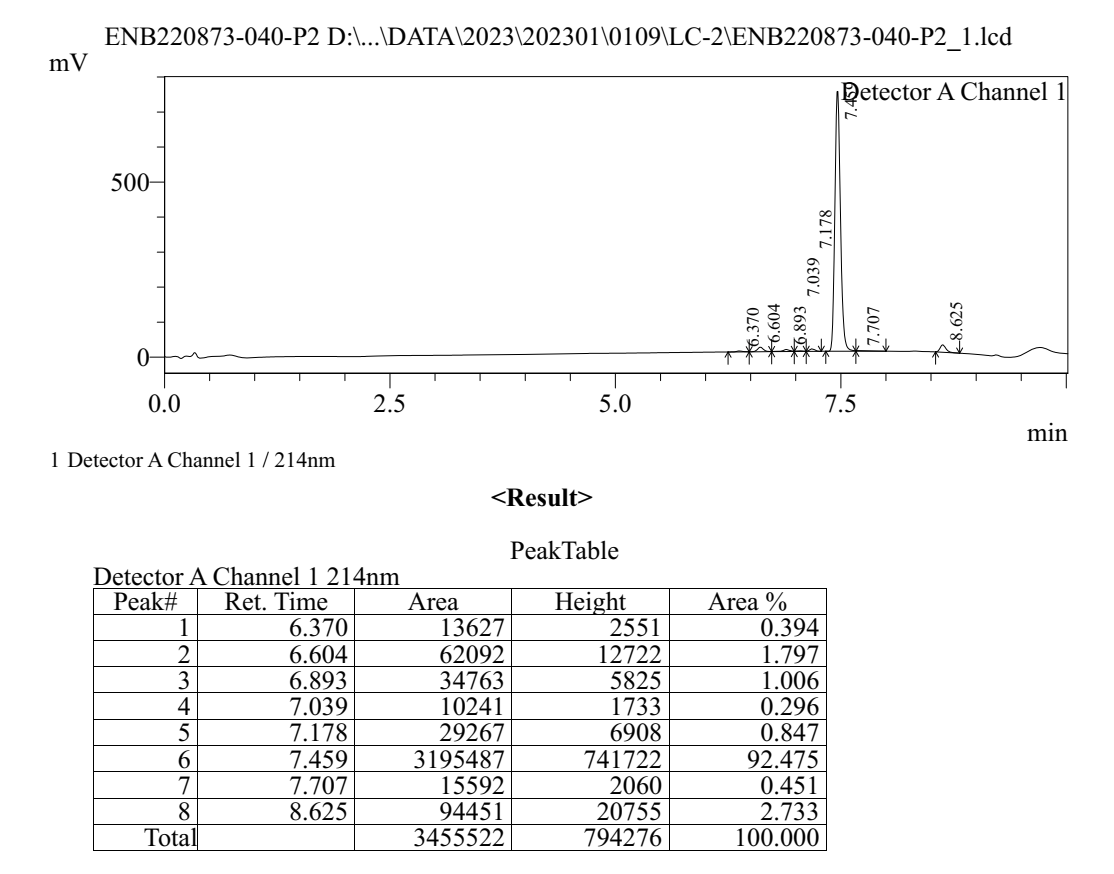
MS chart of compound **16**


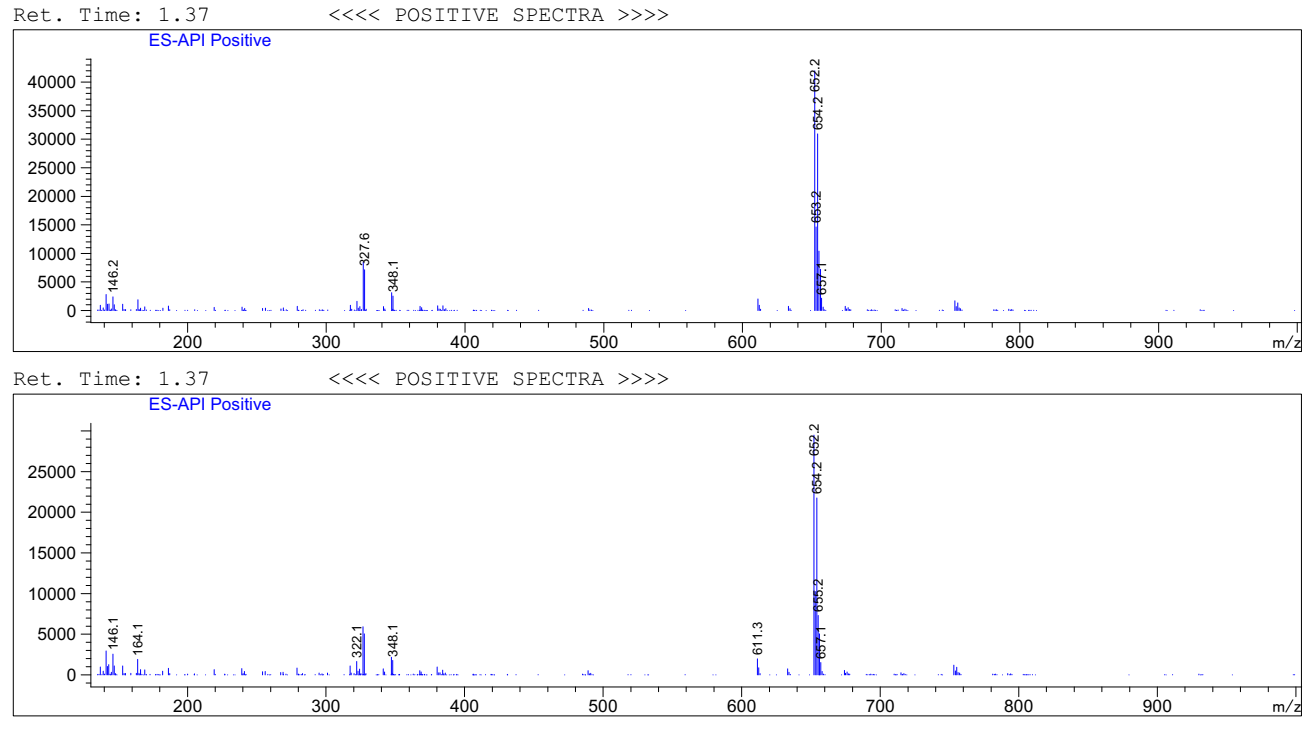


^1^H NMR spectrum of compound **17**


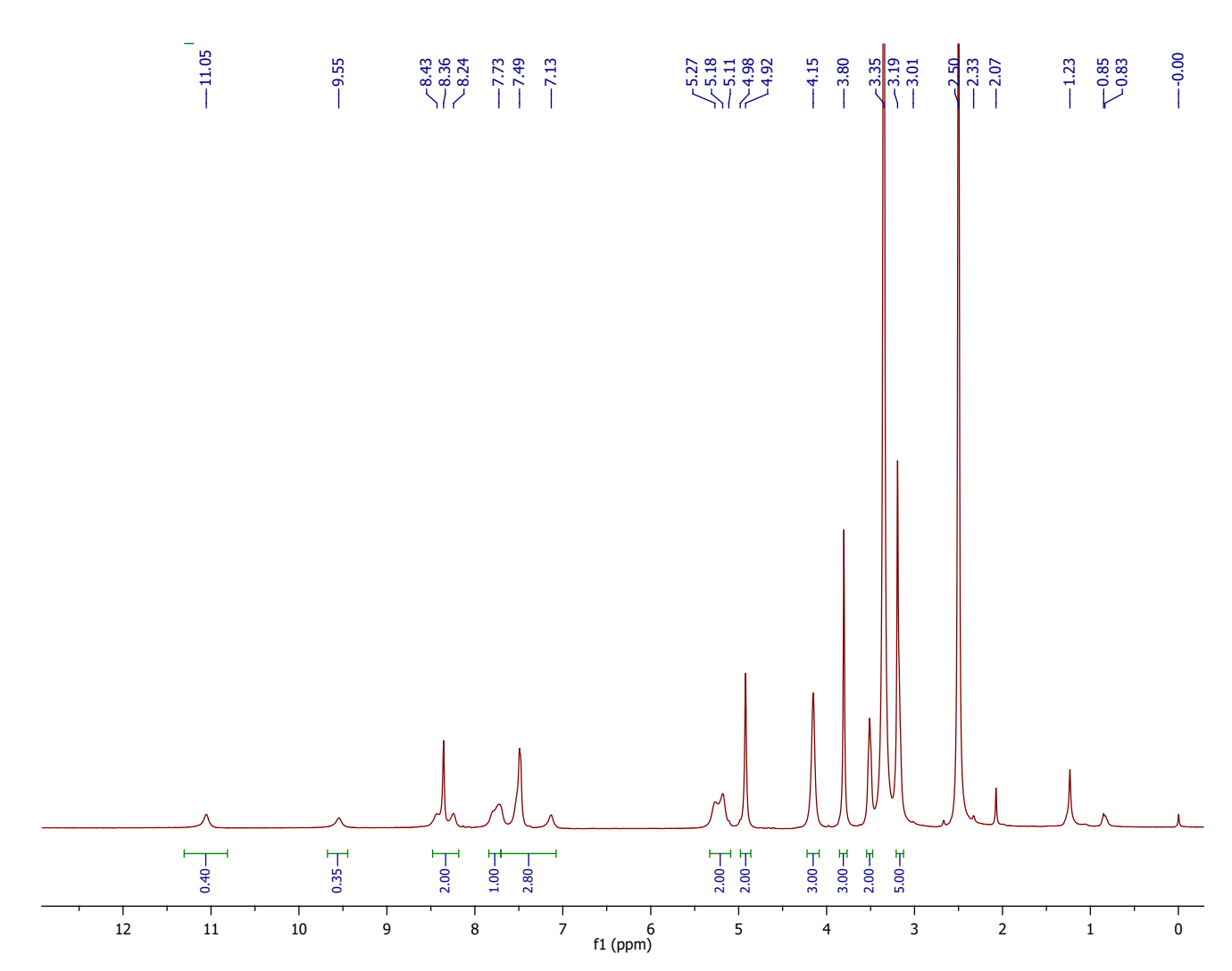


HPLC chart of compound **17**


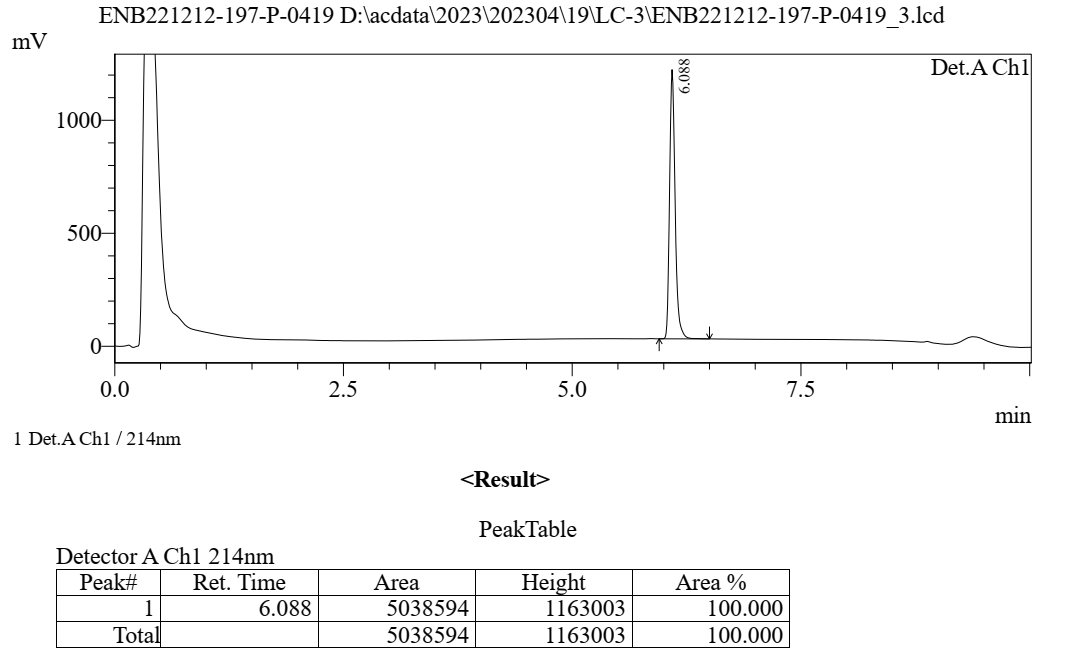


^1^H NMR spectrum of compound **18**


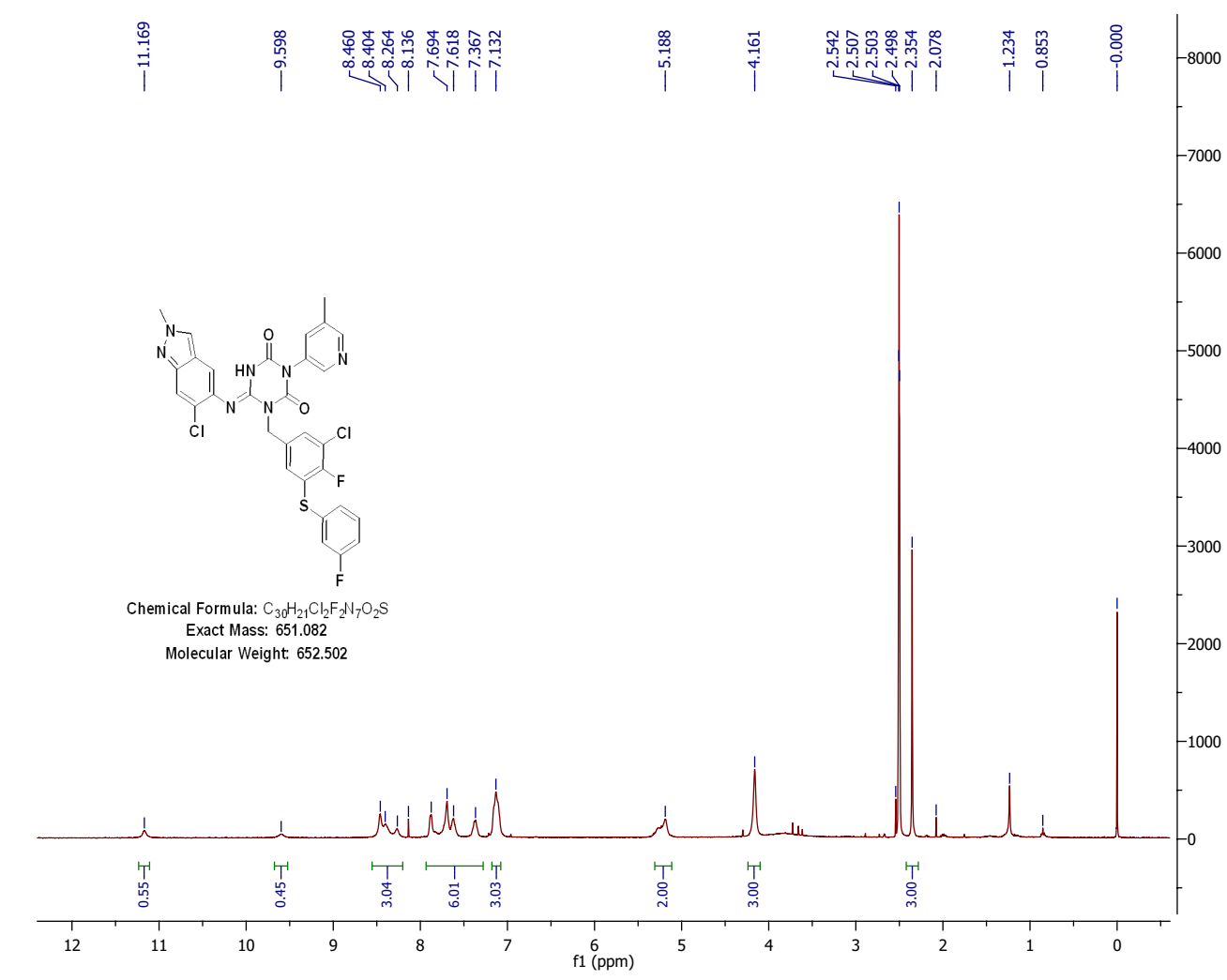


HPLC chart of compound **18**


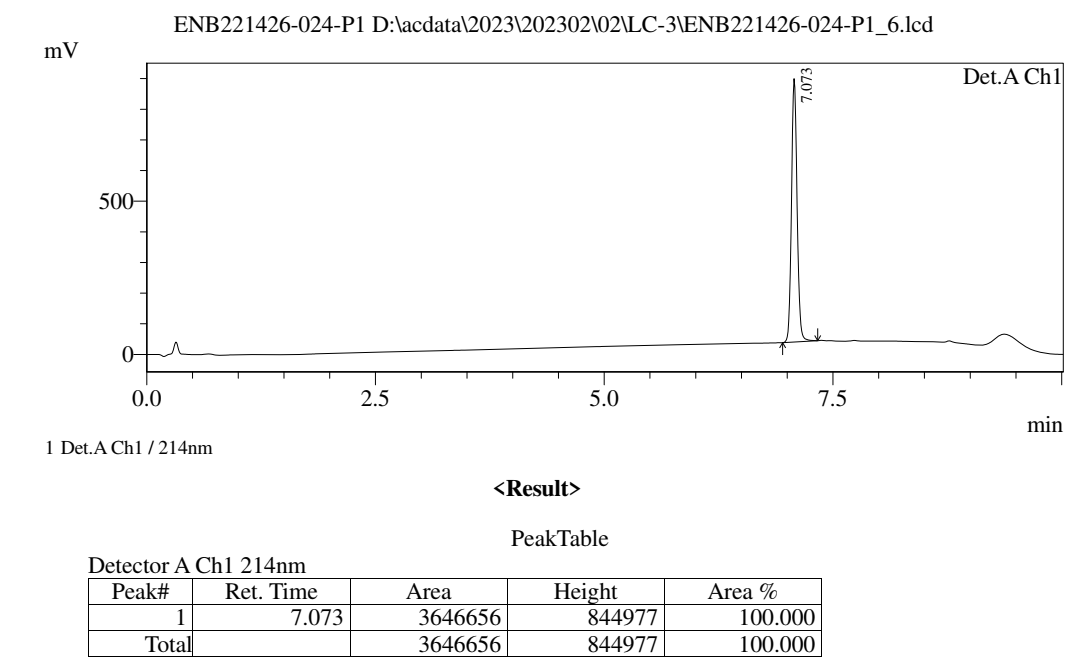


MS chart of compound **18**


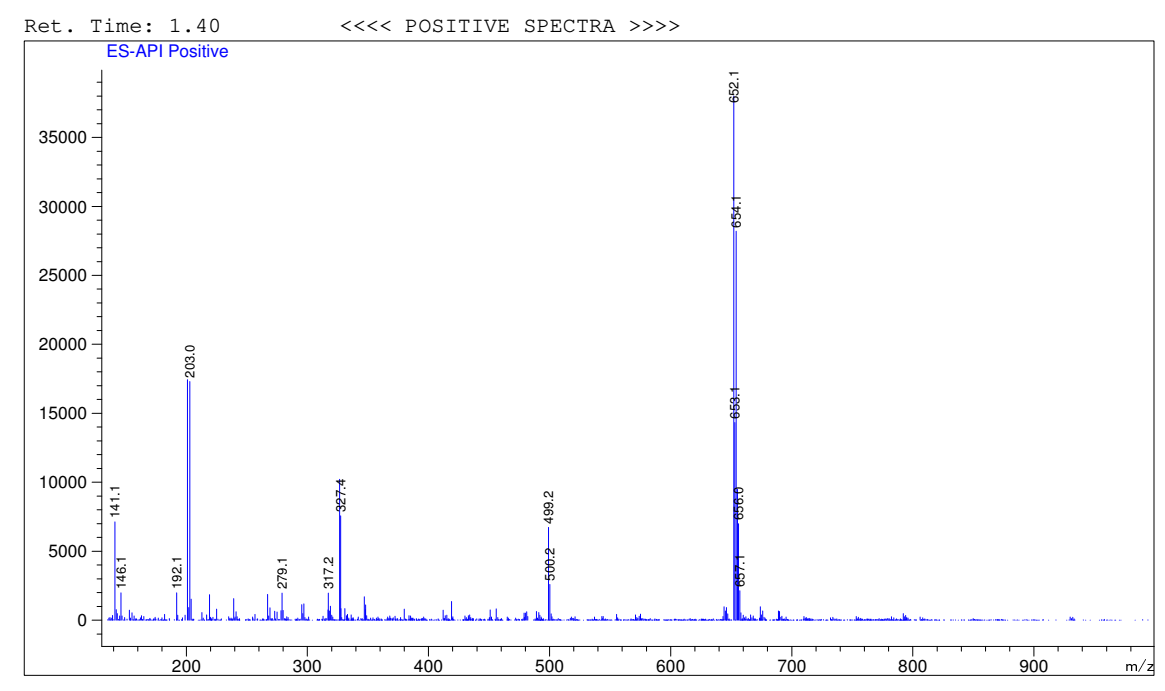


^1^H NMR spectrum of compound **19**


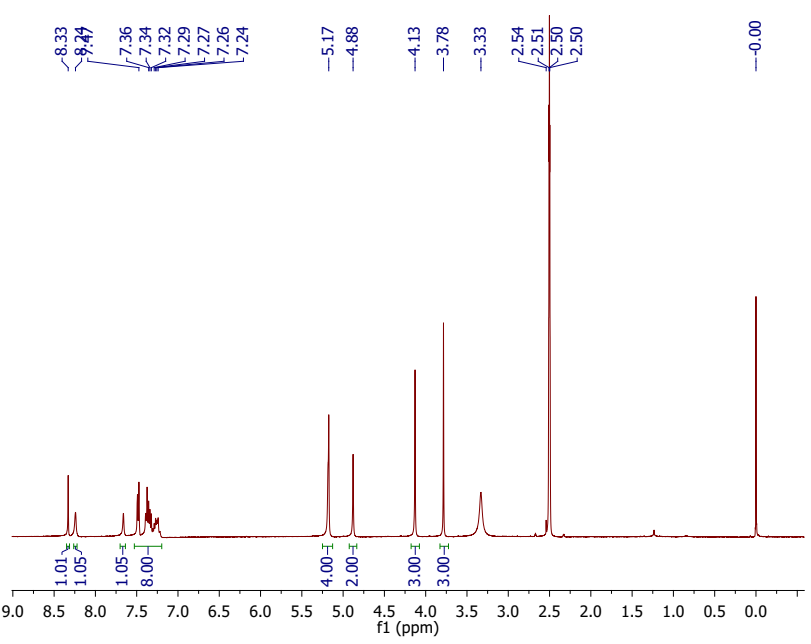


HPLC chart of compound **19**


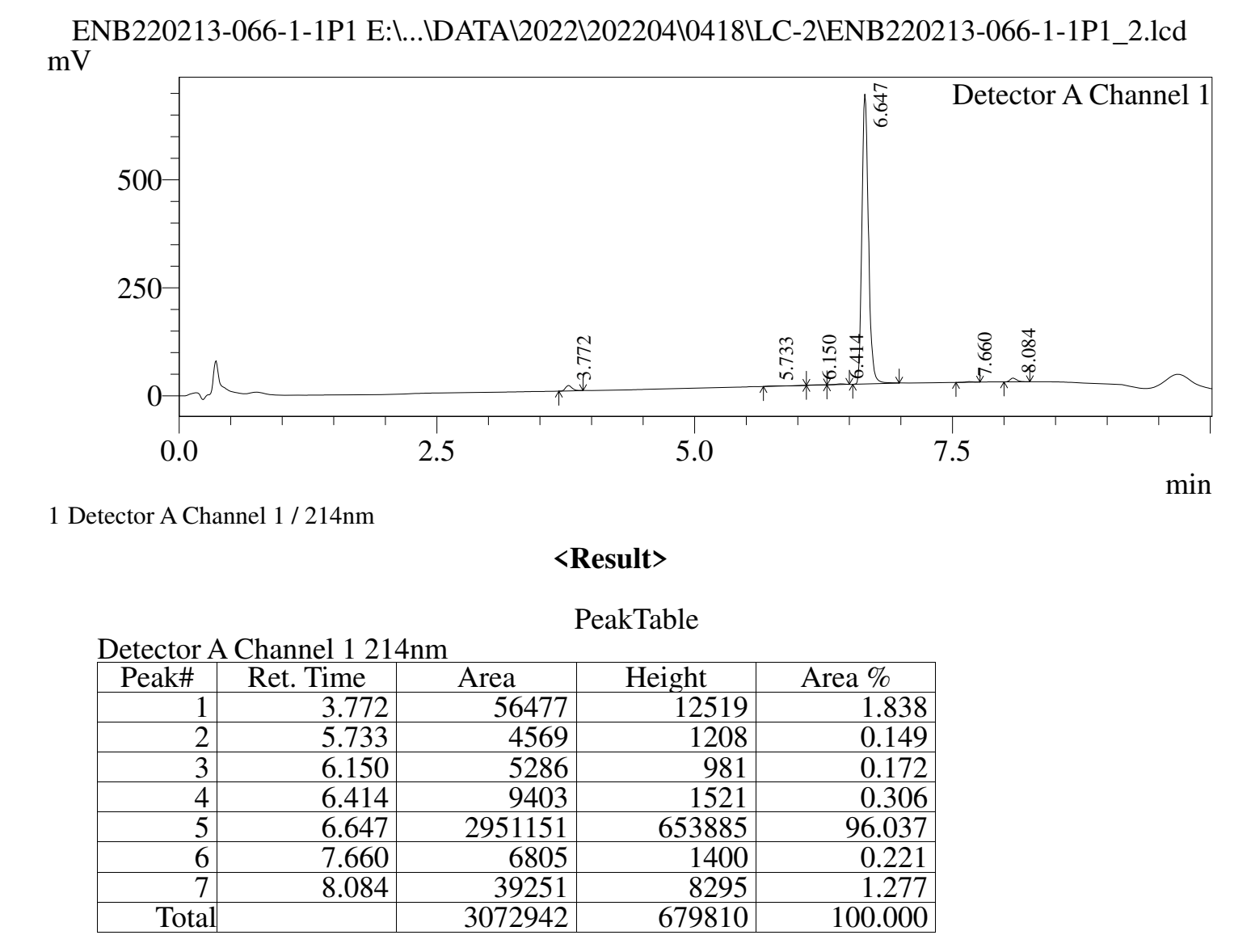


^1^H NMR spectrum of compound **20**


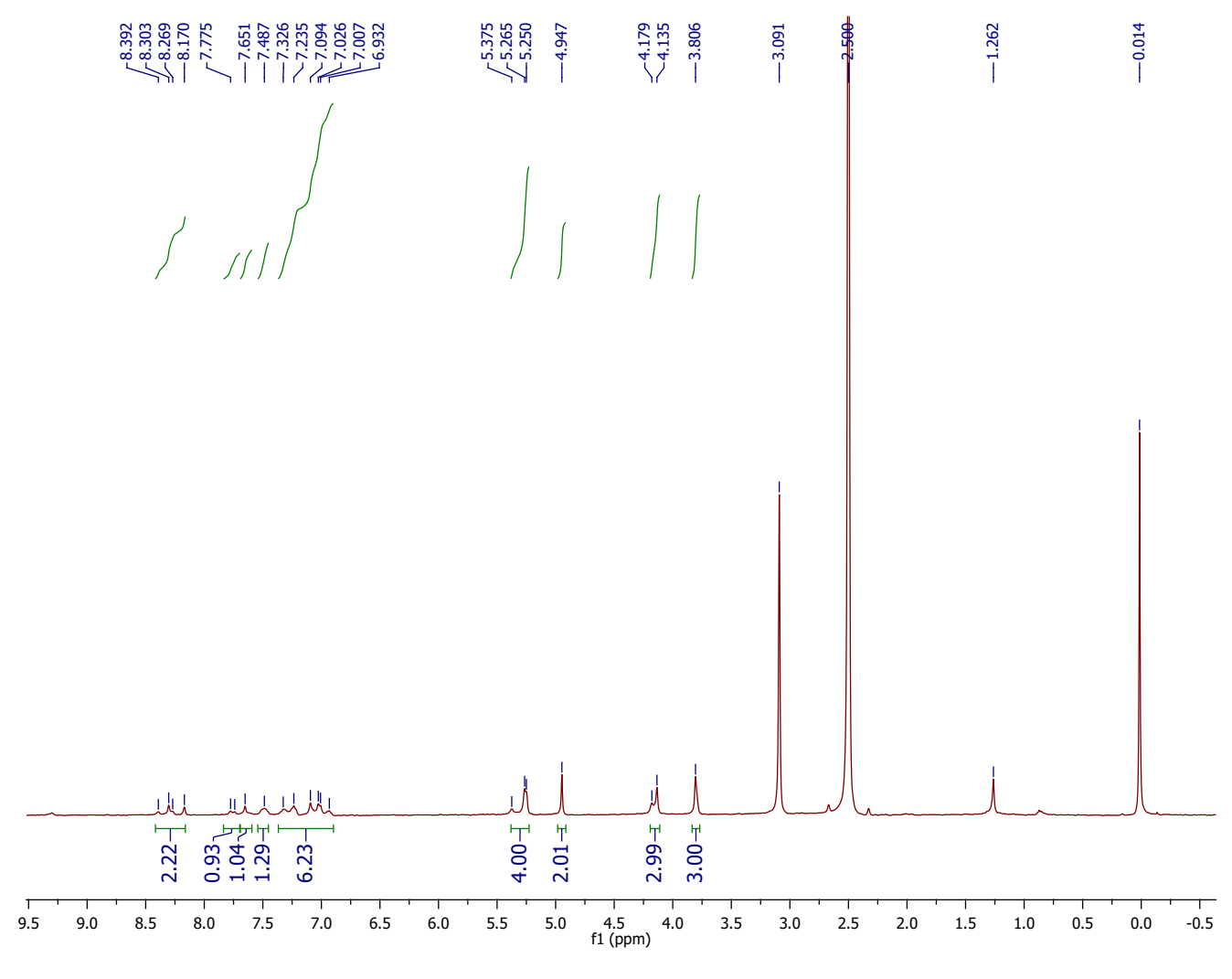


HPLC chart of compound **20**


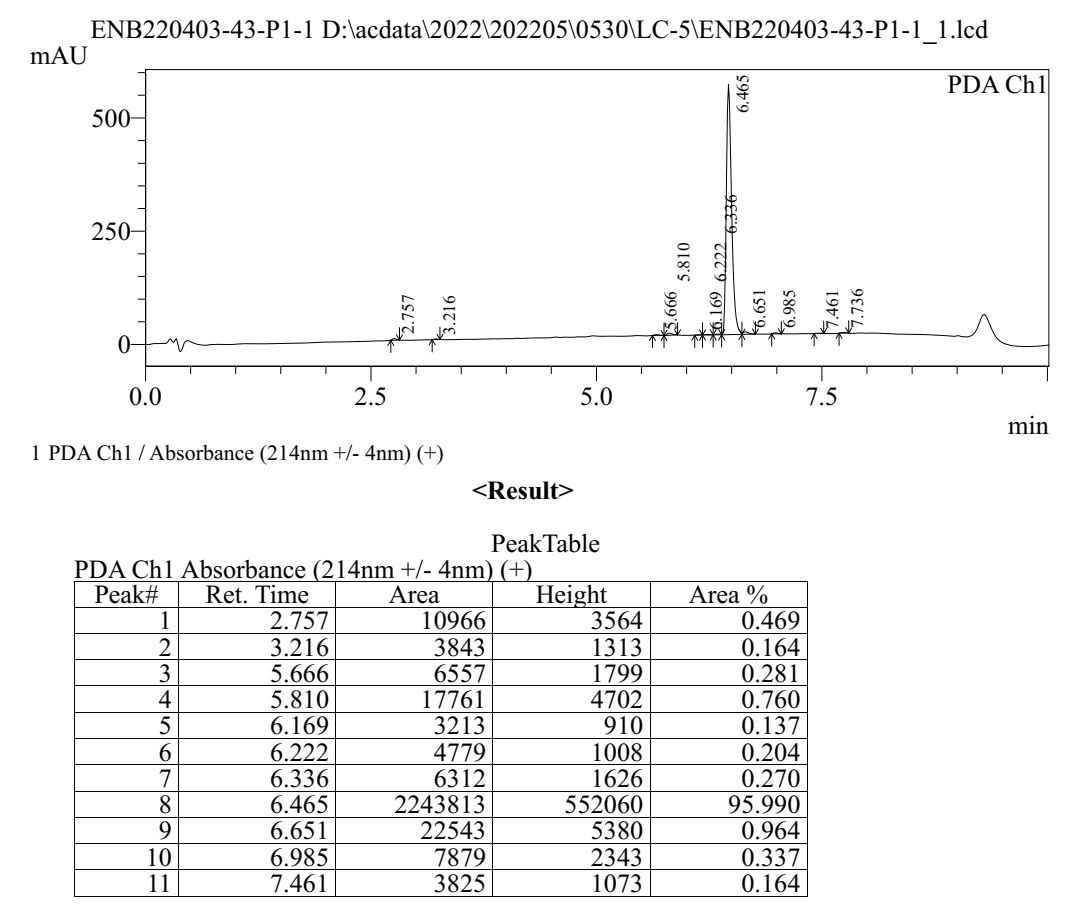


MS chart of compound **20**


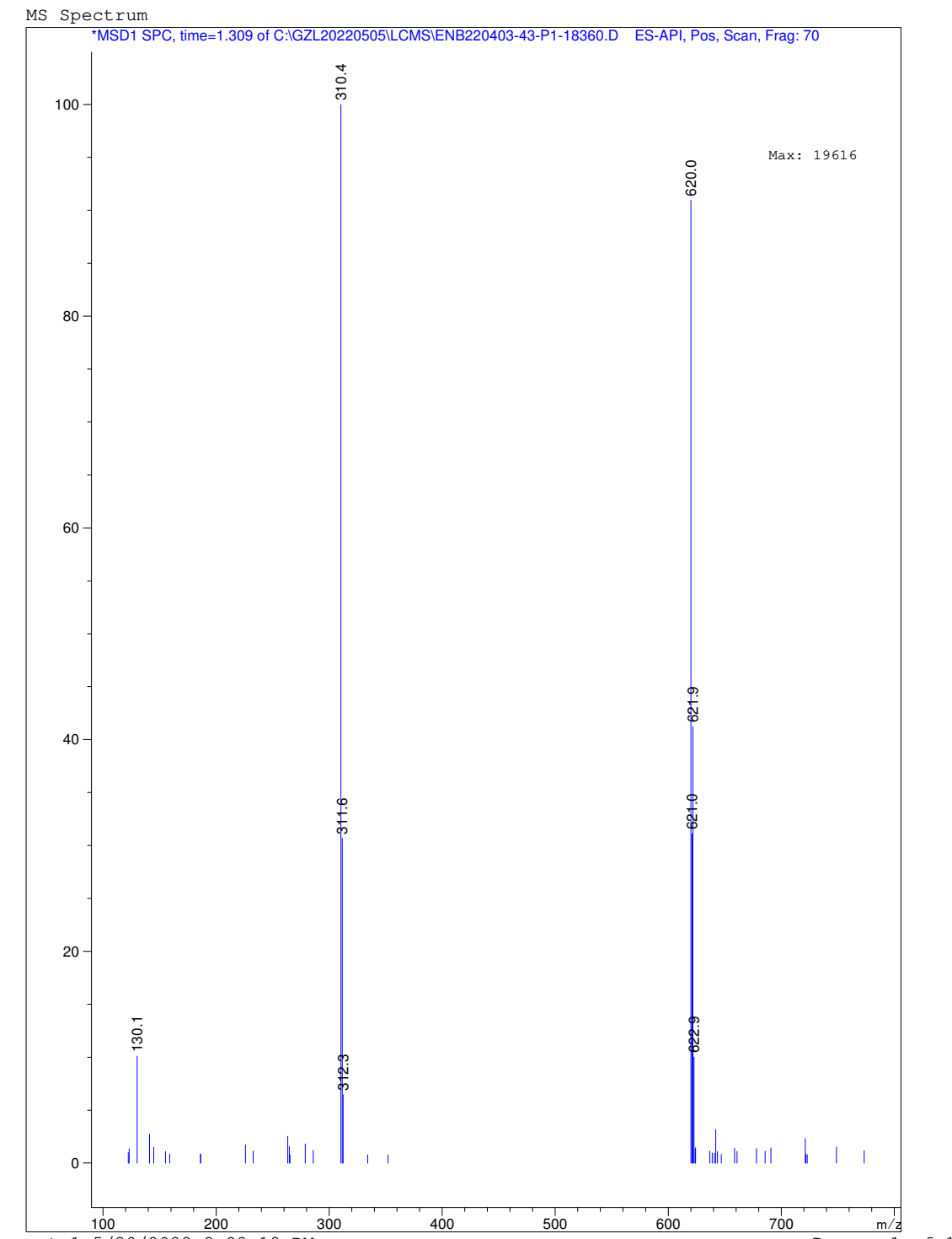


^1^H NMR spectrum of compound **21**

HPLC chart of compound **21**


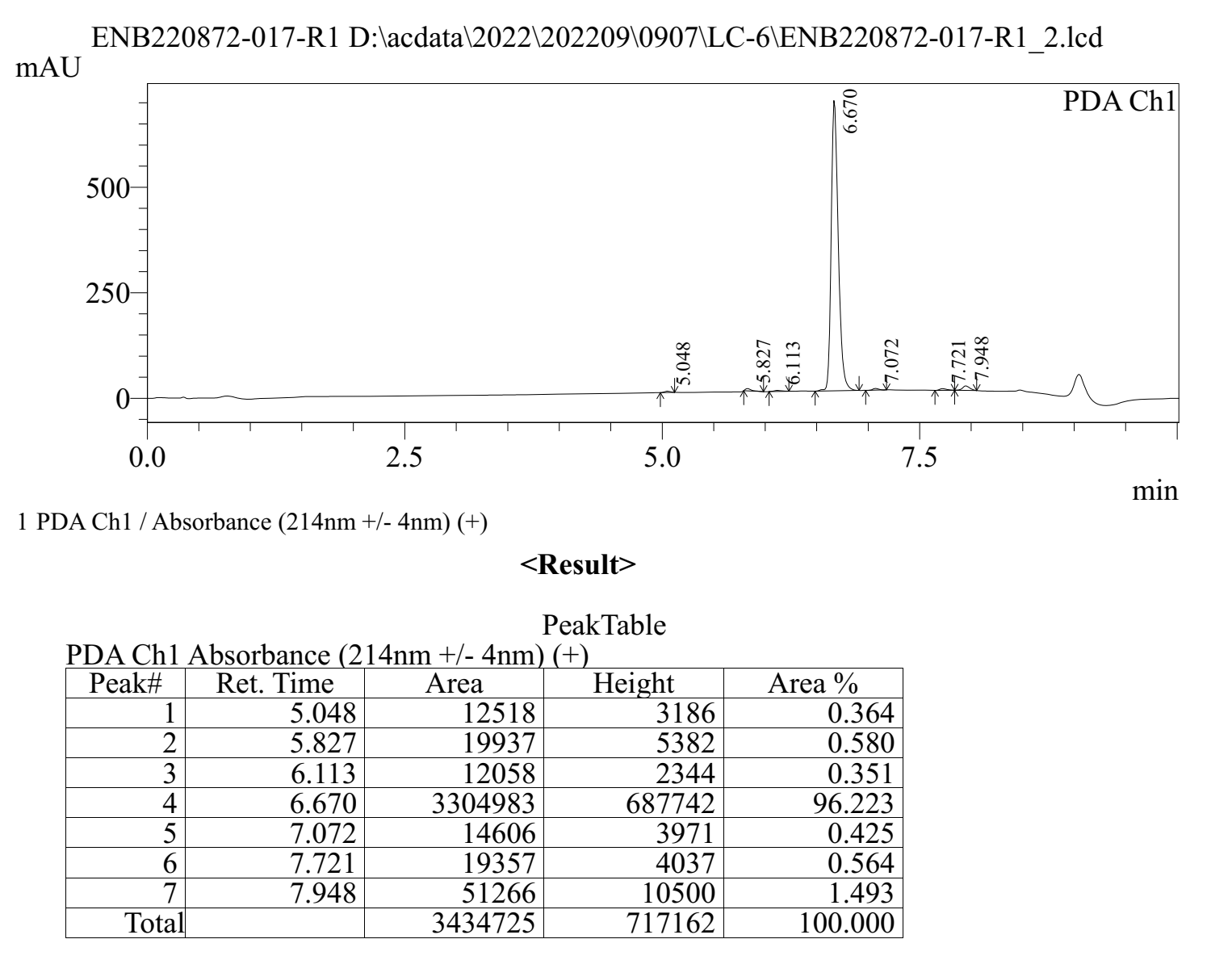


MS chart of compound **21**


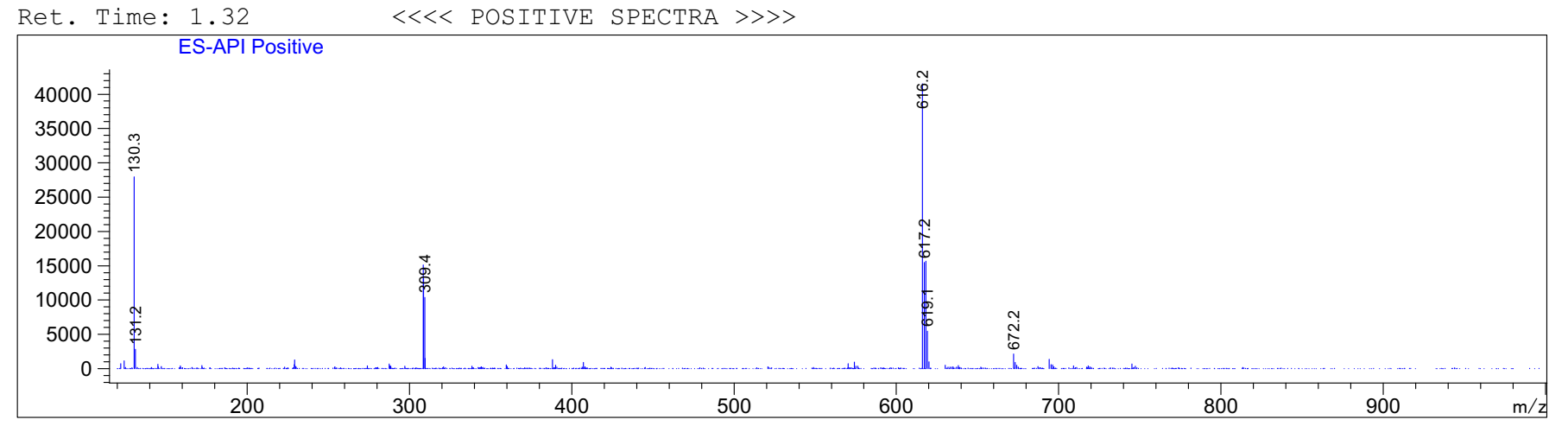


^1^H NMR spectrum of compound **22**

HPLC chart of compound **22**


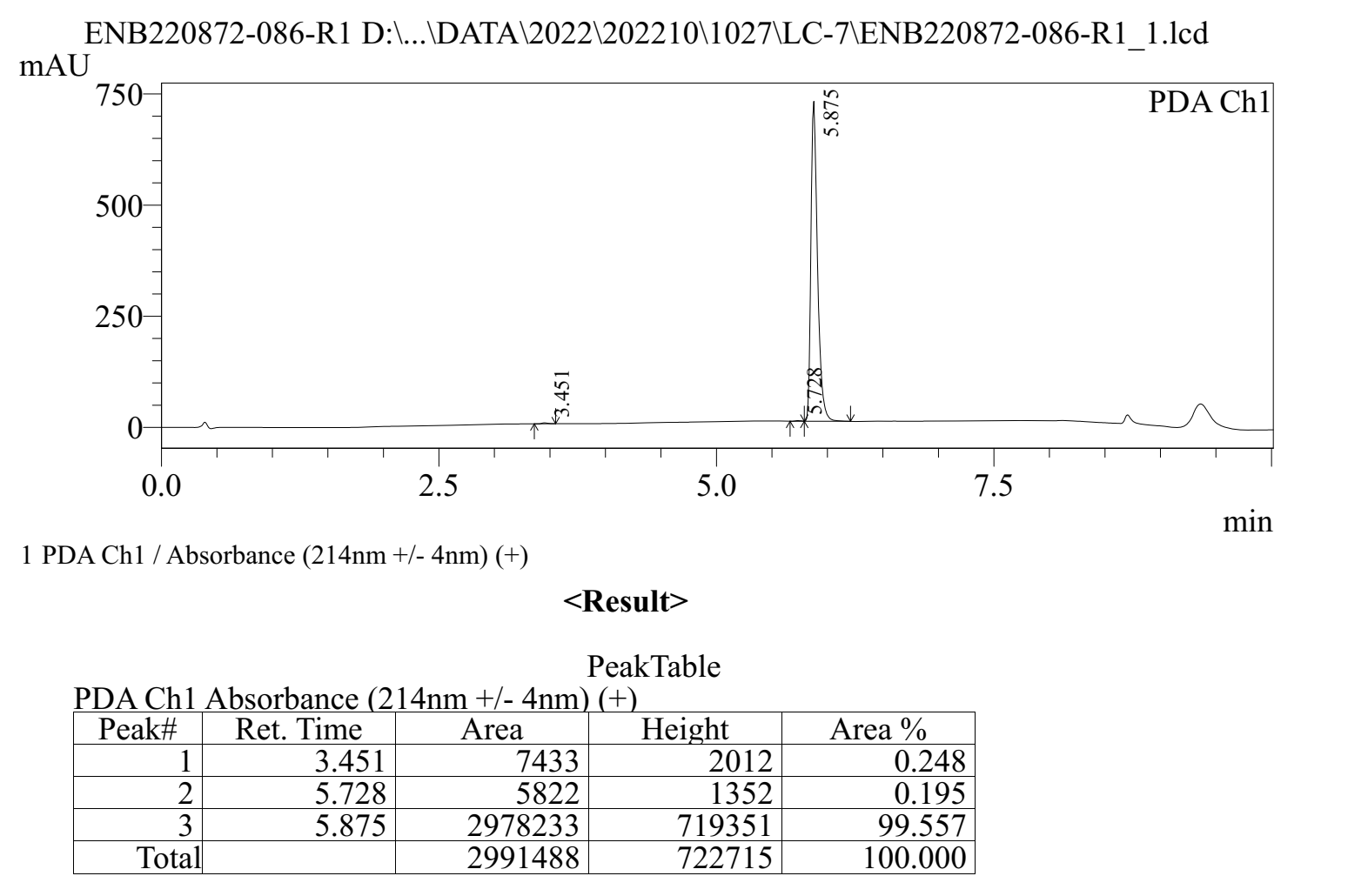


MS chart of compound **22**


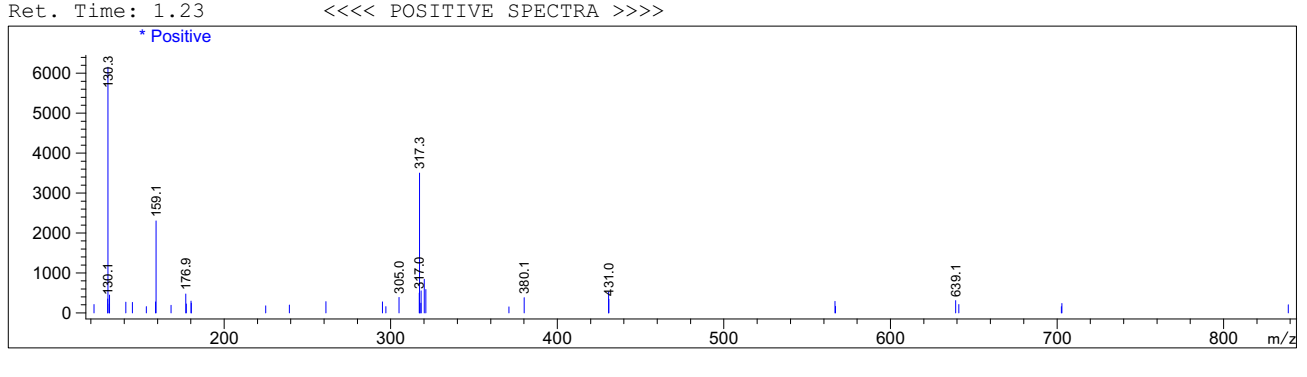


^1^H NMR spectrum of compound **23**


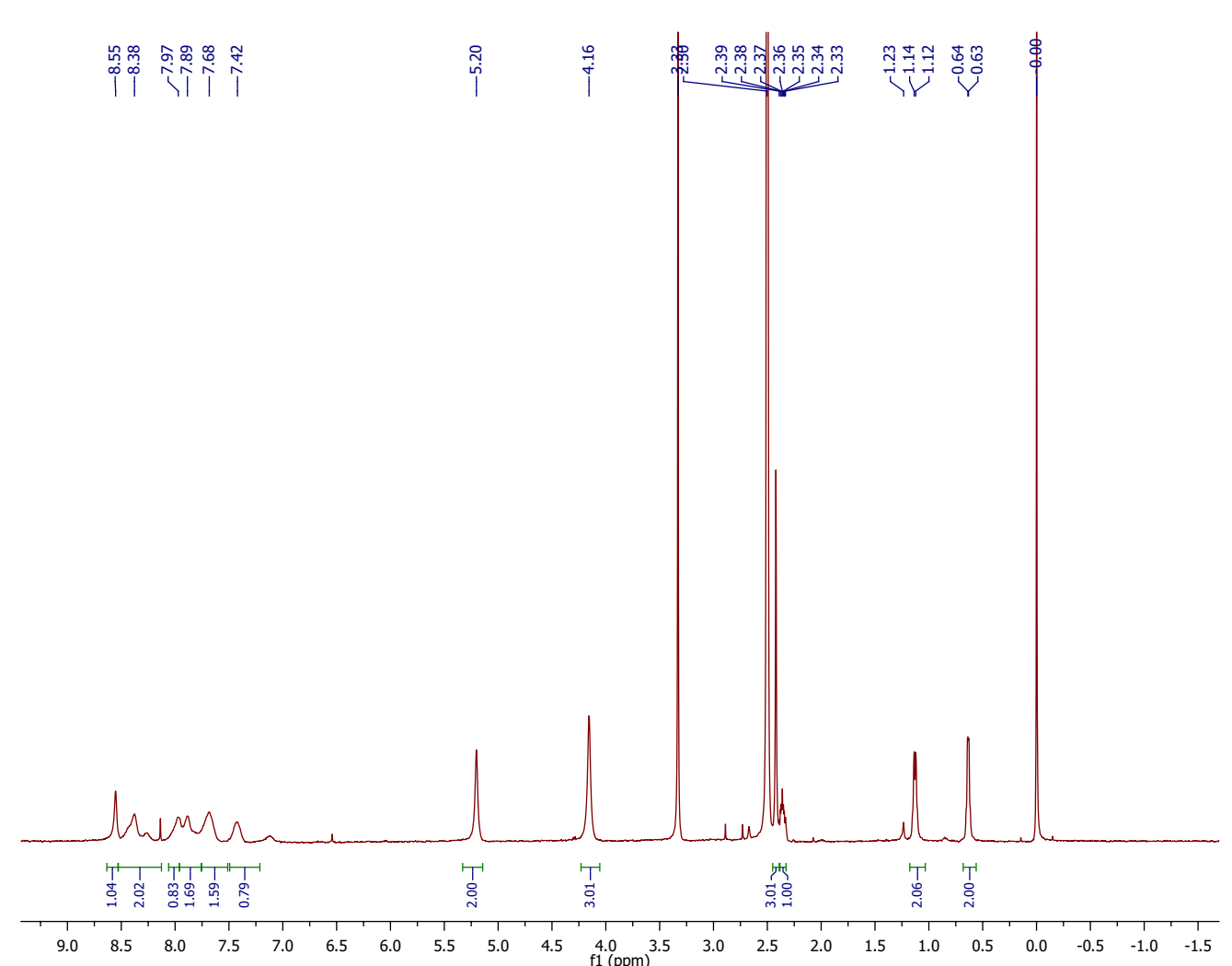


HPLC chart of compound **23**


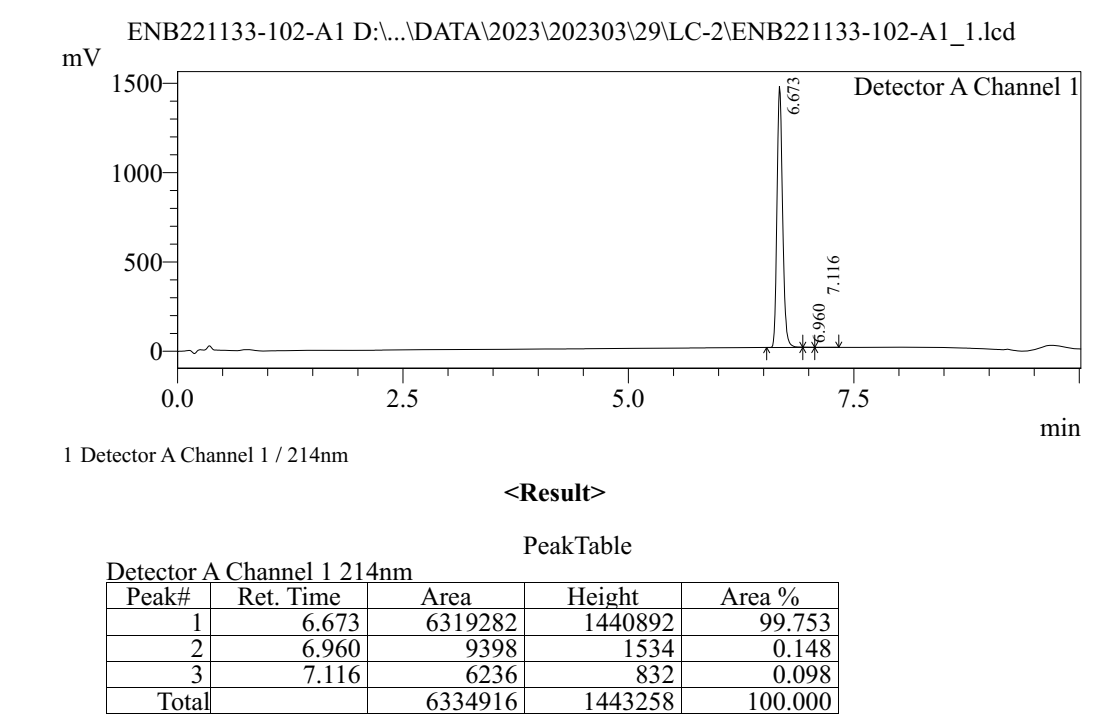


MS chart of compound **23**


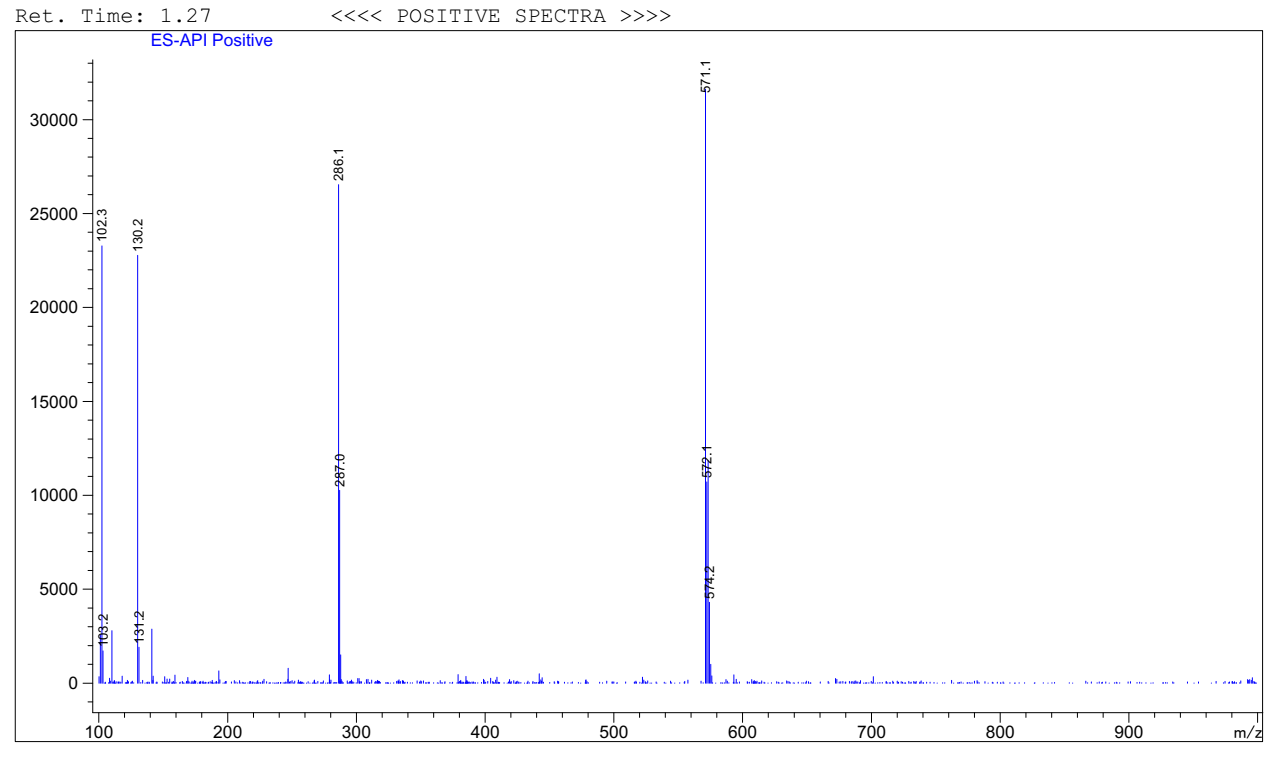


^1^H NMR spectrum of compound **24**


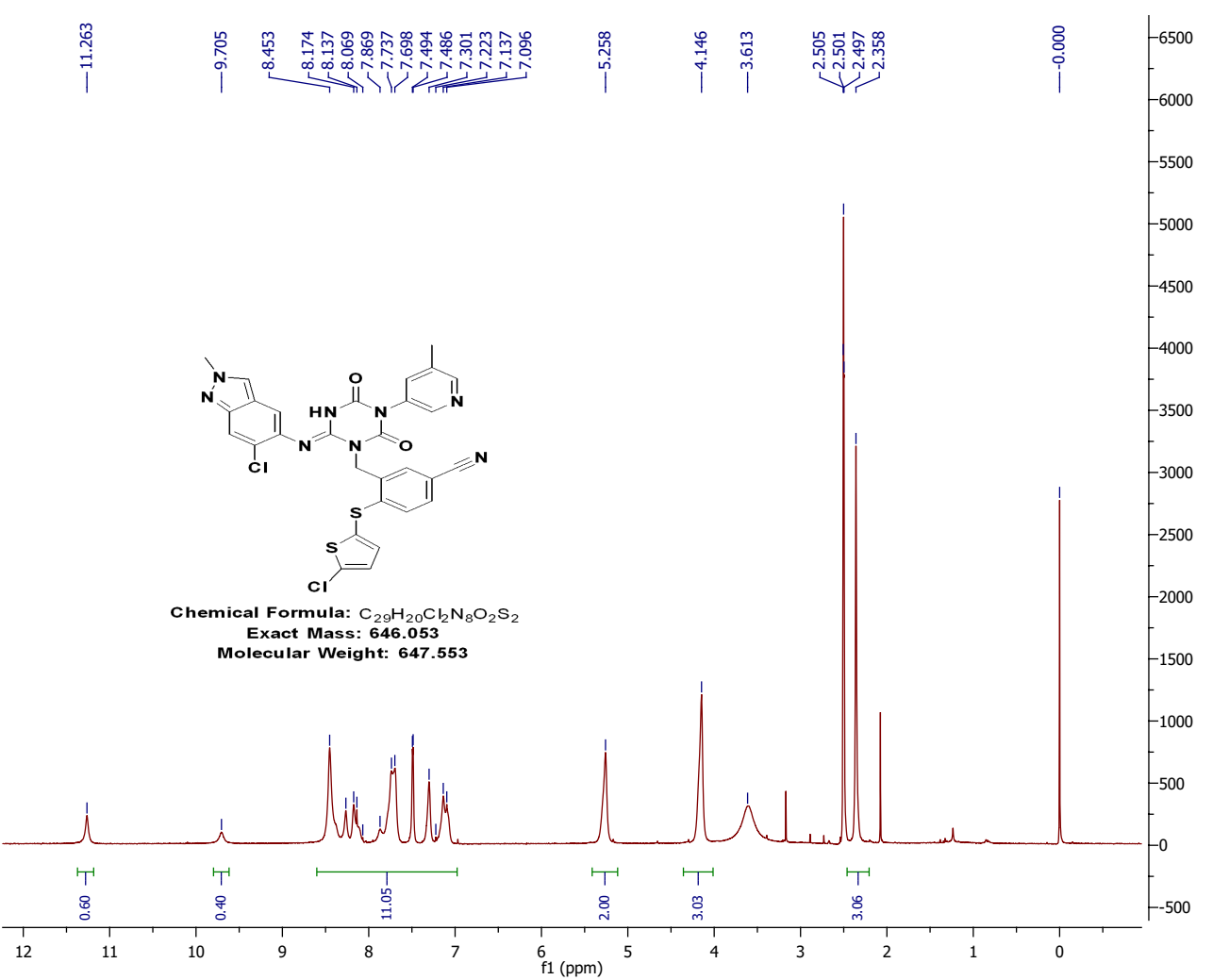


HPLC chart of compound **24**


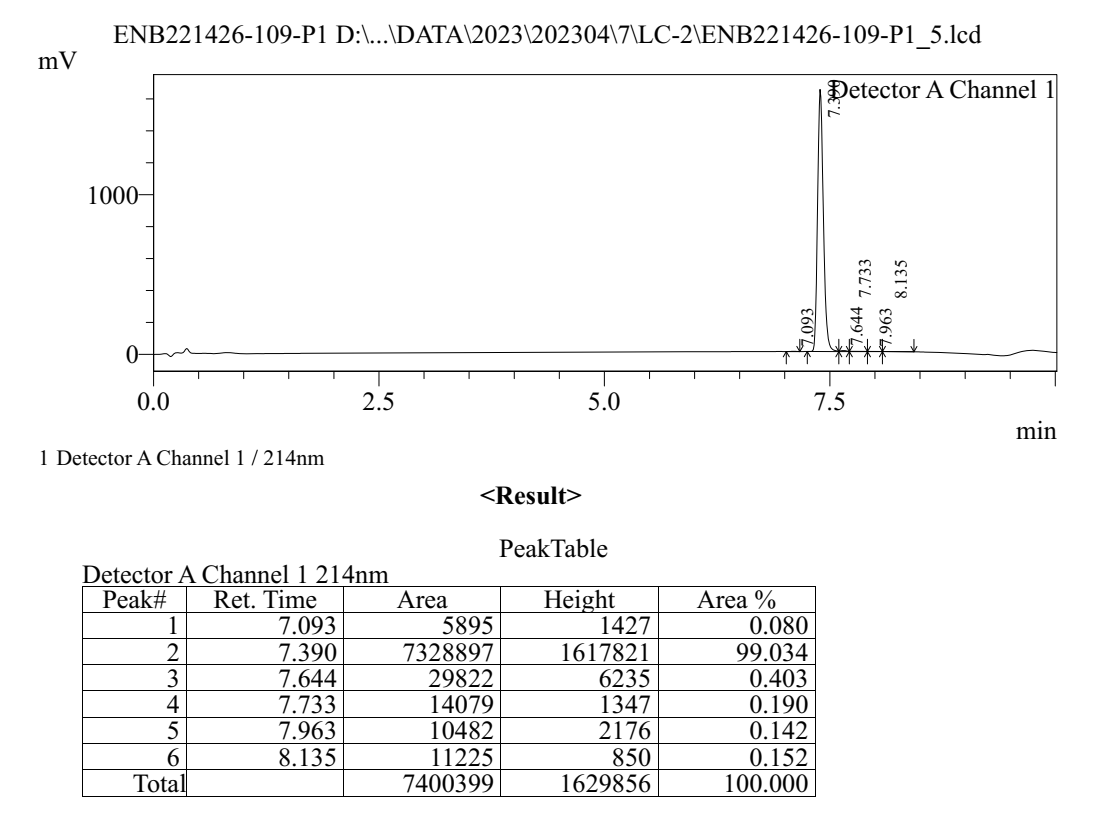
MS chart of compound **24**


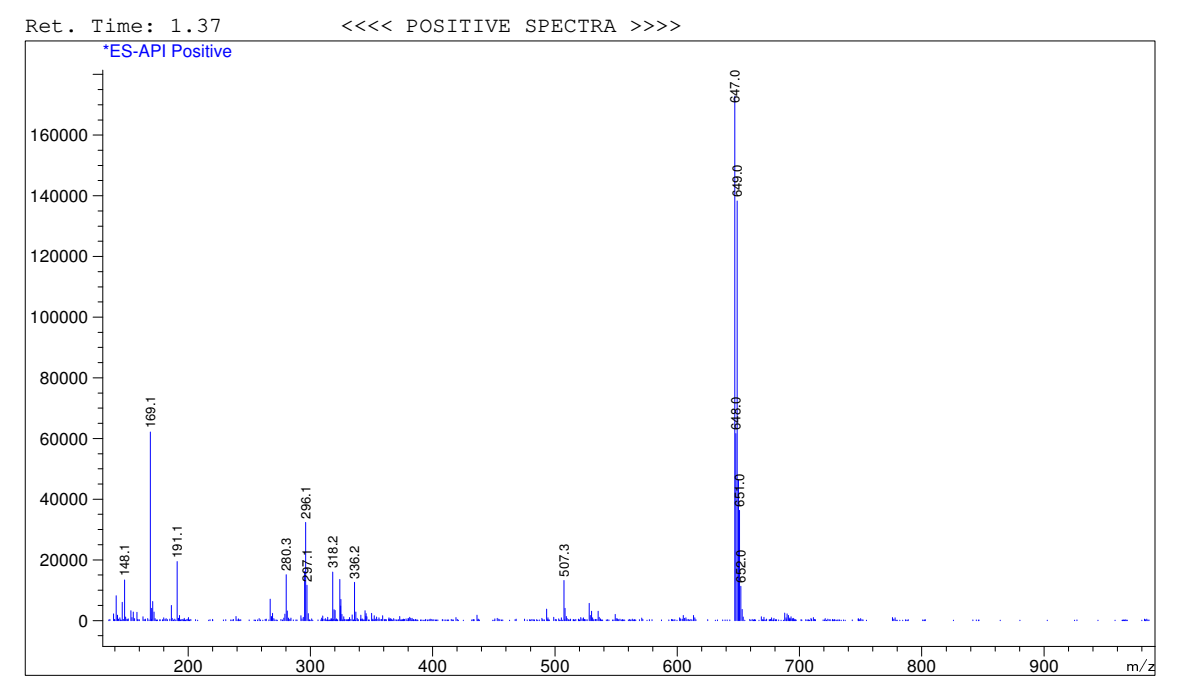


^1^H NMR spectrum of compound **25**


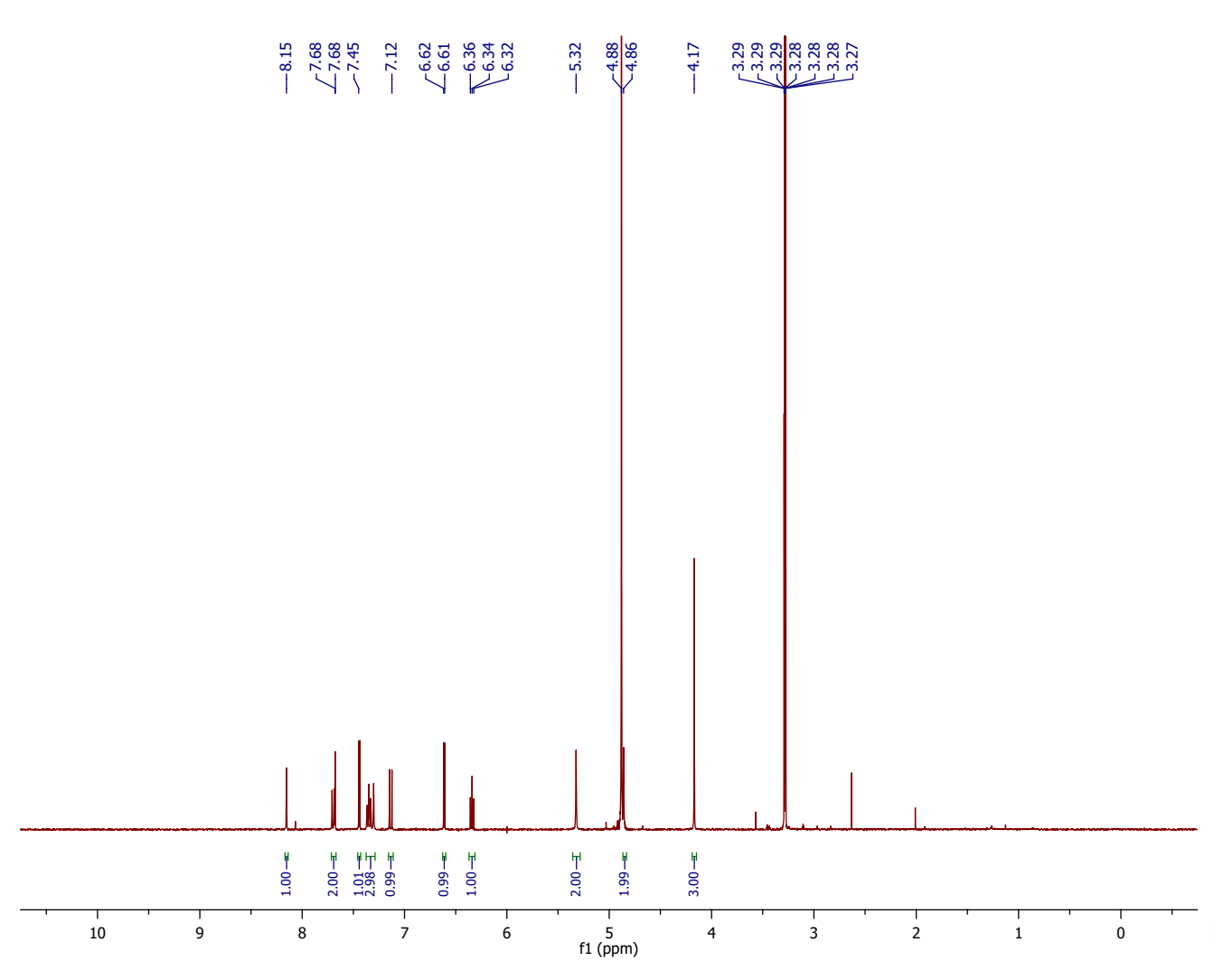


HPLC chart of compound **25**


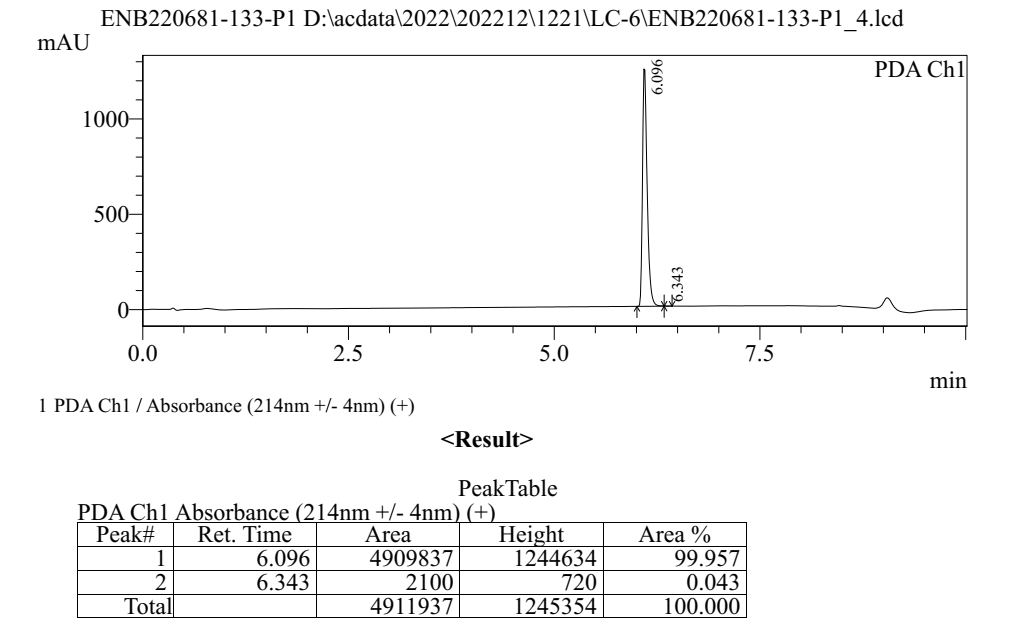


## 6. The source and catalogue number of the chemicals and assays

**Table S2.** Chemicals and assays detailed information

| Chemicals | Source | Catalogue number |
| --- | --- | --- |
| Tetrahydrofuran | Energy chemical | W3100765000 |
| Dichloromethane | Energy chemical | W6101545000 |
| 1,4-dioxane | Adamas-beta | 01375906 |
| N,N-Dimethylformamide | Energy chemical | W6104935000 |
| Acetonitrile | Energy chemical | D0406155000 |
| N-Methyl-2-pyrrolidone | Energy chemical | A0501685000 |
| Triethylamine | Energy chemical | B100065 |
| Trifluoroacetic acid | Macklin | T818778 |
| Tetrabromomethane | damas-beta | 01488136 |
| Triphenylphosphine | Bidepharm | 01094286 |
| Copper(I) chloride | Bidepharm | BD122484 |
| Cesium carbonate | Energy chemical | E060058 |
| 2,2,6,6-Tetramethyl-3,5-heptanedione | Bidepharm | BD64181 |
| N-Bromosuccinimide | Bidepharm | BD40868 |
| 2,2'-Azobis(2-methylpropionitrile) | Bidepharm | 01116476 |
| Bis(pinacolato)diboron | Bidepharm | BD14304 |
| Potassium Acetate | Bidepharm | BD112157 |
| 1,1'-Bis(diphenylphosphino)ferrocene]dichloropalladium(II) | Energy chemical | E060091 |
| Potassium carbonate | Energy chemical | E063596 |
| Lithium bis(trimethylsilyl)amide | Energy chemical | W430005 |
| Copper(II) acetate | Aladdin | C106651 |
| 4-Dimethylaminopyridine | Energy chemical | A050168 |
| Sodium nitrite | 3A | A01374 |
| 1,1'-Carbonyldiimidazole | Bidepharm | BD33959 |
| 1,8-Diazabicyclo[5.4.0]undec-7-ene | Adamas-beta | 01103627 |
| p-Toluenesulfonic acid | Macklin | T890279 |
| N,N-Diisopropylethylamine | Energy chemical | W320014 |
| chlorotrimethylsilane | Energy chemical | W20008 |
| Assays | Source | Catalogue Number |
| PEG 6,000 | Merck | 25322-68-3 |
| MES | Sigma | 4432-31-9 |
| Glycerin | Beyotime | ST1348-1L |
| Imidazole | Merck | 288-32-4 |
| Sodium chloride | Merck | 7647-14-5 |
| Tris | Merck | 77-86-1 |
| EDTA | Biofroxx | 60-00-4 |
| DMSO | Sigma | 67-68-5 |
| BL21(DE3) Competent E. coli | TIANGEN | CB105 |
| MCA-AVLQSGFR-Lys(Dnp)-Lys-NH2 | Beyotime | P9731 |
